# Deviations in effective connectivity explain different hallucination subtypes in Parkinson’s disease psychosis

**DOI:** 10.1038/s44220-026-00669-7

**Published:** 2026-06-10

**Authors:** Miriam Vignando, Dominic Ffytche, Ndabezinhle Mazibuko, Giulio Palma, Anjali Bhat, Marcella Montagnese, Sonali Dave, Yen F. Tai, Lucia Batzu, Valentina Leta, K. Ray Chaudhuri, Caroline H. Williams Gray, Mitul A. Mehta

**Affiliations:** 1 Centre for Neuroimaging Sciences, Institute of Psychiatry, Psychology and Neuroscience, King’s College London, London, UK; 2 Institute of Psychiatry, Psychology and Neuroscience, King’s College London, London, UK; 3 Department of Psychology, University of Southampton, Southampton, UK; 4 Department of Psychology, University of Exeter, Exeter, UK; 5 Department of Clinical Neurosciences, Herchel Smith Building, University of Cambridge, Cambridge, UK; 6 University of London, London, UK; 7 Imperial College London, Faculty of Medicine, Department of Brain Sciences, The Hammersmith Hospital, London, UK; 8 Parkinson Foundation Centre of Excellence, King’s College Hospital NHS Foundation Trust, London, UK; 9 Fondazione IRCCS Istituto Neurologico Carlo Besta, Department of Clinical Neurosciences, Parkinson and Movement Disorders Unit, Milan, Italy; 10 John Van Geest Centre for Brain Repair, Department of Clinical Neurosciences, University of Cambridge/Cambridge University Hospitals NHS Foundation Trust, Cambridge, UK

**Keywords:** Parkinson's disease, Computational neuroscience, Parkinson's disease, Cognitive ageing

## Abstract

Psychosis and visual hallucinations (VH) in Parkinson’s disease (PD) substantially impact patient outcomes, yet the underlying neural mechanisms remain unclear, limiting effective treatments. Here we used dynamic causal modeling to leverage the fast temporal dynamics captured with electroencephalography data during a visual mismatch negativity task in people with PD with (*N* = 20) and without (*N* = 18) VH to examine effective connectivity. We found reduced top-down and enhanced bottom-up connectivity in ventral visual and prefrontal regions during task performance in PD-VH, suggesting deficits in sensory prediction updating and an overreliance on visual input. Connectivity patterns differed with hallucination complexity, with complex VH being associated with altered top-down and bottom-up right-hemisphere connectivity, and multimodal hallucinations to more widespread bilateral disruption. Increased task activity, as computed with source reconstruction, correlated positively with normative serotonergic 5-HT_2A_ receptor distribution. These findings highlight specific neural targets for early therapeutic interventions, supporting a transdiagnostic computational architecture of hallucinations.

## Abstract (web summary)

In this study, Vignando et al. investigate neural alterations in visual processing in people with Parkinson’s disease with visual hallucinations compared with those without visual hallucinations.

## Main

Parkinson’s disease (PD) is characterized by motor and nonmotor symptoms^1^, among which, psychosis and visual hallucinations (VH) can have a substantial impact on patient outcomes and are associated with cognitive decline^2–4^. Although morphological^5–7^ and functional^7–9^ alterations differentiate patients with PD with VH (PD-VH) from those without VH, standard cognitive assessments do not provide mechanistic insights into VH. A recent review of frameworks^10^ converges on the proposal that ‘ascending’ sensory disturbances together with ‘descending’ factors (for example, expectations, specific object features and attention) contribute to VH. Among the different proposals, hierarchical predictive coding, a cognitive model of psychosis, suggests that hallucinations arise from disrupted balance between top-down expectations and bottom-up sensory inputs, with a stronger influence of expectations observed in people with hallucinations^11–13^. Mismatch negativity (MMN) is an event-related potential computed by averaging the time-locked brain activity that can be observed when a rare stimulus is presented within a sequence of frequent stimuli in an electroencephalography (EEG) recording^14^. MMN is widely considered to be a robust marker of psychosis^15^, thought to reflect an attempt to minimize this prediction error and capture its disruption^16–19^. We recently demonstrated reduced visual MMN responses in PD-VH^20^, paralleling auditory MMN impairments observed in psychosis^15^. However, traditional event-related potential (ERP) analyses cannot uncover the directional neural mechanisms involved. Dynamic causal modeling (DCM), leveraging EEG’s temporal precision, enables exploration of these mechanisms^21–23^. DCM has proved instrumental in clarifying the disruption of MMN in people with schizophrenia-related psychosis^24^, and a spectral DCM study (using resting-state functional magnetic resonance imaging (fMRI)) found increased resting-state top-down and reduced bottom-up connectivity in the visual network in PD with VH, a pattern that was predictive of hallucination severity^8^. Here, we employ DCM with ERP neural mass models across dorsal (primary visual area, V1, and inferior parietal lobe (IPL)) and ventral (primary visual, V1 and inferior temporal gyrus (ITG)) visual pathways interacting with the prefrontal cortex (PFC) during a visual MMN task. The main aim of this study is to investigate the possible neural mechanisms and effective connectivity patterns underlying the reduced visual MMN observed in PD-VH, using DCM to test the hierarchical organization proposed by the predictive-coding framework^11–13^. Second, we aim to investigate the relation of changes in effective connectivity to hallucination complexity. We include the PFC to test prediction error impairment, because top-down connectivity is crucial for integrating sensory information^25,26^. We focus on the dorsal and ventral pathways on the basis of relevant literature^20,27–33^ and because changing the line orientation in our MMN task is a process known to involve specific parietal regions^34^. We hypothesize reduced top-down and enhanced bottom-up connectivity at task in PD-VH, reflecting impaired prediction updating and reliance on compromised sensory information, as visual input is known to be defective in PD-VH^35^. In addition, exploratory analyses relate MMN-derived source activity to cortical distributions of receptors, previously implicated in PD-VH.

## Results

**Fig. 1:**
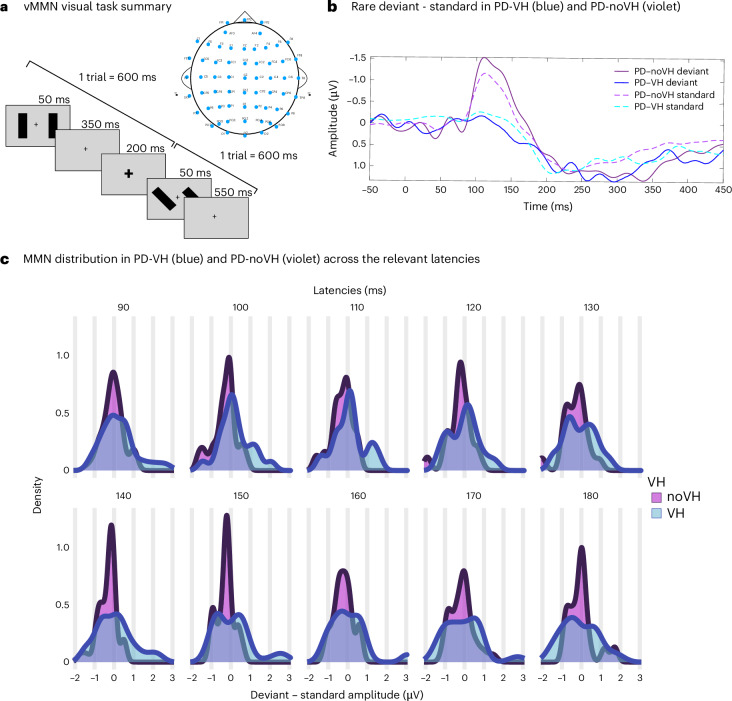
Summary of the vMMN task and findings. The overt task required participants to press a button when the fixation cross became bigger and a different button when the fixation cross became smaller. **a**, Visual summary of a trial (600 ms): the fixation cross remained on the screen for the whole duration of the experiment; changes in cross size lasted 200 ms; bars flashed peripherally every 50 ms, changing in orientation (90°, 130° and 160°). **b**, vMMN ERP for PD with and without VH (POZ shown (*F*(1,36) = 4.32, MS = 289.821, *P* = 0.045); data published^20^). **c**, Density plots showing the distribution of the amplitude of MMN in PD and PD-VH at electrode POZ: PD without VH showed overall greater negativity at POZ compared with PD-VH; PD-VH (left column) showed almost no negativity in this time frame, as confirmed by the analyses reported in our ERP study and summarized in **b**. The density plots highlight how PD-noVH show a consistency in negativity that is lost or variable in PD-VH. Panel **a** adapted with permission from ref. ^20^ courtesy of the authors. Source data

Participants did not differ on sex (*χ*^2^ = 0.01, *P* = 0.91; 6 females in the PD-VH group (*N* = 20), 7 females in the PD group (*N* = 18) and 13 males in each group), age, disease duration, motor symptoms, levodopa equivalent daily dose (LEDD) or Montreal Cognitve Assessment (MoCA) score (Supplementary Fig. 1 and Supplementary Tables 1 and 2). Two participants were taking antipsychotic medication (one patient with PD-VH was taking clozapine and one patient with PD-noVH was taking olanzapine, prescribed for depressive symptoms in this specific case). Two participants with PD-VH and two with PD were on citalopram and sertraline for depressive symptoms. Two participants with PD-VH were taking rivastigmine. The number of participants with PD-VH and PD-noVH taking dopamine agonists did not statistically differ (Supplementary Information 1 and Supplementary Table 3). A one-way analysis of variance (ANOVA) confirms that participants of both groups were paying attention to the screen and executing the task, as participants with PD-VH and PD-noVH did not differ on misses; further, the groups did not differ in reaction times. Participants with PD-VH were less accurate than those with PD-noVH in detecting the type of cross-change, but their performance was above chance level (see Fig. 1, Supplementary Information 2 and Supplementary Table 3 for individual medication information).

### Sensor space and source reconstruction analyses identify occipital and temporal sources

**Fig. 2:**
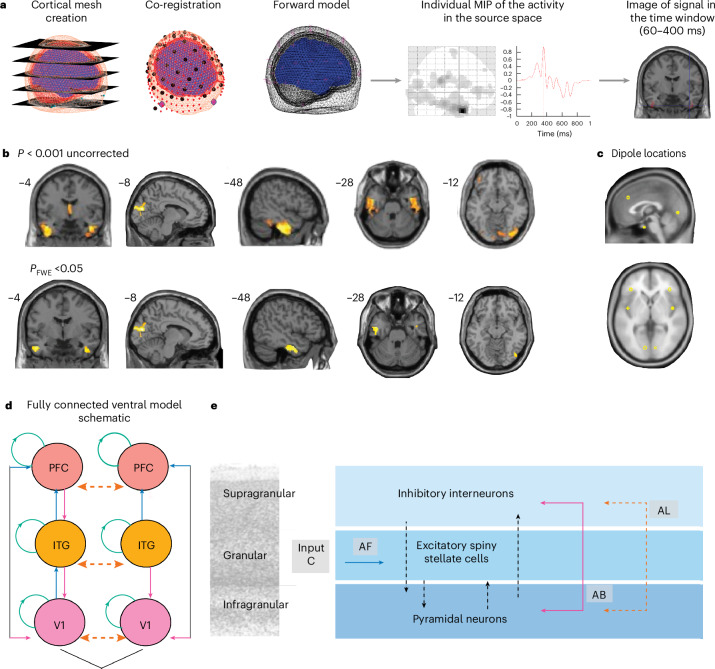
Source reconstruction methods, results and ventral model specification. **a**, The main steps of source reconstruction: cortical mesh creation, projection on structural template, co-registration of EEG signal to the template and maximum intensity projection (MIP) for each participant, allowing us to conduct analyses in sensor space (Supplementary Information 3 for details) and to generate a 3D image for the time window of interest (here, 60–400 ms). **b**, Source reconstruction results for the rare deviant trials are shown (*P* < 0.001 uncorrected in the top row and *P*_FWE_ < 0.05 in the bottom row). The one sample *t*-test for vMMN in the whole sample for signal source reconstruction for the rare deviant, on images generated for the 60–400-ms interval (*P* < 0.001, height threshold *F* = 11.74, d.f. (1.74), *k* > 200) returned clusters in the inferior and anterior temporal gyrus, bilaterally and in lateral occipital regions (the occipital–calcarine cluster remains significant after FWE correction: *P* = 0.026 cluster and *P* = 0.031 peak). We also found a large medial thalamic cluster (*k* = 725, *P* = 0.07). We also conducted paired-samples *t*-tests in each group separately, comparing deviant with standard, which returned a significant peak (*k* = 145, *P* < 0.001 uncorrected and *P*_FWE_ = 0.014) in the bilateral calcarine cortex, mirroring the results of the standard and deviant source reconstruction analysis (Supplementary Fig. 2b; see Supplementary Information 4 for coordinates). For the PD-VH group, a significant peak (*k* = 83, *P* < 0.001 uncorrected and *P*_FWE_ = 0.021) was found in the left ITG (fusiform region) (Supplementary Information 4). All participants were included in the analysis (PD-VH *N* = 20, and PD-noVH *N* = 18). The brain maps presented have been produced with xjview (https://www.alivelearn.net/xjview). **c**, Dipole locations selected on the basis of the results presented in **a**, here presented on a template of the brain. Dipole coordinates: V1 left –10, –82, –0.5, right 10, –82, –0.5; left ITG –44,–4, –34, right 44, –4, –34; and left PFC –38, 33, 35, right 38, 33, 35. **d**, A schematic of model connections: AF, forward connections in blue; AB, backward connections in fuchsia; AL, cross-hemispheric connections in orange; input (C matrix). Connections included in the task modulatory matrix (B matrix) for the winning model v3 specified forward connections from V1 to ITG, V1 to PFC and ITG to PFC, and backward connections from ITG to V1, PFC to ITG and PFC to V1. Lateral (inter-hemispheric) connections between regions were also specified between left and right V1, ITG and PFC. Green circles represent connectivity on the same region, testing for self-connectivity during task conditions. **e**, A visual representation of the ERP neural mass used as described in ref. ^69^. Source data

Analyses in the sensor space, including all EEG electrodes, confirmed that the channels involved in the task returned temporal and frontal locations (*P* < 0.001; S3) and parieto-occipital (*P* value after family-wise error correction for multiple comparisons, *P*_FWE_ < 0.05; Supplementary Information 3). The *t*-test conducted for source reconstruction returned clusters in the inferior temporal gyrus and in lateral occipital regions (*P*_FWE_ < 0.05) and in the thalamus (*P* < 0.001 and *P*_FWE_ = 0.07) (Fig. 2 and Supplementary Information 4).

### vMMN differences correlate with decreased top-down connectivity in PD-VH

**Fig. 3:**
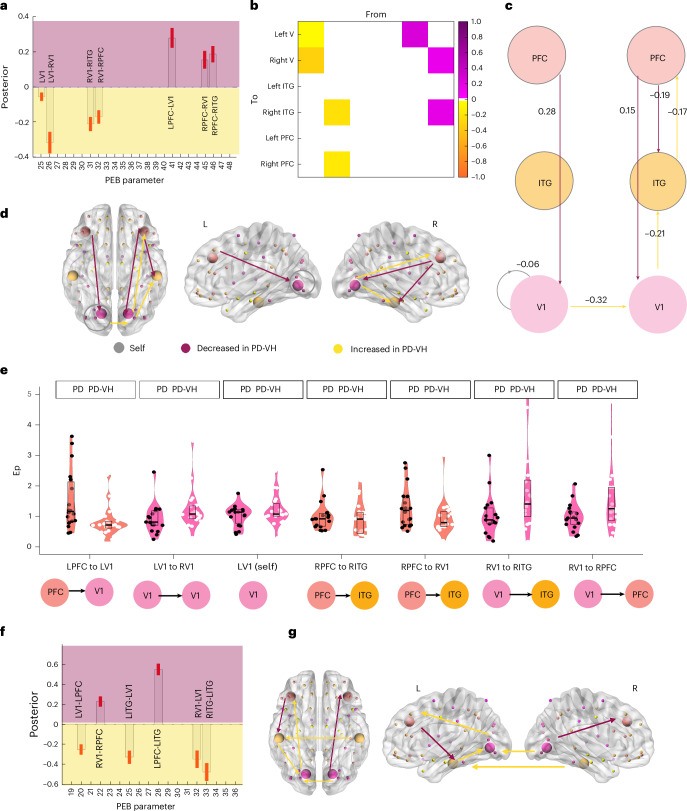
PEB results. **a**, A bar plot of estimated effective connectivity for each connection surviving the pp > 0.99 (free energy-based model evidence) threshold. Each panel shows the posterior estimates (model-derived quantities) from PEB of the DCM parameters: the *x* axis indexes the PEB parameter and the *y* axis shows the posterior estimate for the group effect. The background has been shaded to indicate direction only, with purple indicating a decreased strength in PDVH, and yellow indicating an increased strength in PD-VH (negative values correspond to increased connectivity in PD-VH, as the PD-VH group was classified as −1 in the design matrix, as opposed to 1 for PD). The red bars indicate the standard error. **b**, The connectivity matrix of results presented in **a**. The matrix shows how top-down effective connectivity is reduced versus increased bottom-up connectivity in VH in this task. From, regions sending the influence; To, regions receiving the influence (the first column in the matrix shows connectivity from left V1 to left V1, right V1, the second from right V1 to right ITG and right PFC, the 4th and 5th columns show decreased connectivity from left and right PFC at task). **c**, A schematic of BMA results with PEB values. Yellow represents increased strength in PD-VH and purple indicates decreased strength in PD-VH (negative values represent increased connectivity in PD-VH as the PD-VH group was classified as −1 in the design matrix, as opposed to 1 for PD). The negative value for self-connection in the left V1 (gray) area indicates less negative self-inhibition. This is consistent with the recursive PEB analysis reported in the main text, as well as in Supplementary Information 6 and Supplementary Fig. 7. **d**, A graphical representation of the connections; color coded as in **a**,**b**. The brain and nodes were generated with the BrainNetviewer (https://www.nitrc.org/projects/bnv/). **e**, Plots showing the distribution of Ep in PD with and without VH for each connection found differing in the model: the violin and box and whisker plots are color-coded with the color of the node from which the connection starts. The dots are color-coded by group: noVH participants (18) with black dots and VH participants (20) with white dots. In each box plot, the centerline indicates the median, the box bounds indicate the interquartile range (25th–75th percentiles) and the whiskers extend to the minimum and maximum values. **f**, Connectivity differences in PD-VH and PD-noVH: a bar plot of estimated effective connectivity for each connection surviving the pp > 0.99 (free energy) threshold. Yellow represents increased connectivity in PD-VH and purple represents decreased connectivity in PD-VH. Connectivity is overall increased in PD-VH in all the connections modelled (all with pp >0.99). The brain and nodes were generated with BrainNet viewer. Latent connectivity A matrix: a graphical representation of the connections, color-coded as in **a**,**b**. **g**, Latent connectivity A matrix: a graphical representation of the connections, color-coded as in **a**,**b**. The brain and nodes were generated with BrainNet viewer. Source data

We carried out Bayesian model selection (Supplementary Fig. 4) on the individual DCMs with a fixed-effect design to determine which of our ventral stream models (fully connected model) best fit the data. We then carried out second level analyses with parametrical empirical Bayesian analyses (PEB), finding that the task stimulus condition (rare versus standard) for PD-VH was associated with increased bottom-up effective connectivity and decreased self-inhibition for V1, and with decreased top-down connection strength (posterior probability (pp) >0.99) (Fig. 3). When including age as a covariate, the connections show the same pattern, with additional reduced ITG-to-V1 connectivity in the left hemisphere (Supplementary Fig. 8). Recursive PEB model comparisons revealed the highest evidence for models including self-inhibitory parameters, although the free energy difference relative to the next-best model was modest with Δ*F* = 188 (S6 and Supplementary Fig. 7).

PEB analyses on latent connectivity (matrix A) show that, for PD-VH, there is an overall increase in effective connectivity both top-down and bottom-up, with a reversed pattern for some of the connections if compared to task activity (Fig. 3f,g). Recursive PEB for the A matrix revealed that the highest evidence is for the cross-hemispheric/lateral model, with increased interhemispheric connectivity in the ITG (pp >0.99). Nevertheless, we interpret this cautiously, as interhemispheric connections may be difficult to identify robustly in source-level EEG^36^, with the possibility of increasing model complexity without necessarily improving model evidence^21,22^. When including age as a covariate, the connections show the same pattern with a widespread increased connection strength in both bottom-up and top-down, especially PFC to V1 bilaterally, connections (Supplementary Fig. 8).

### Hallucination severity, subtype and multimodality analysis

**Fig. 4:**
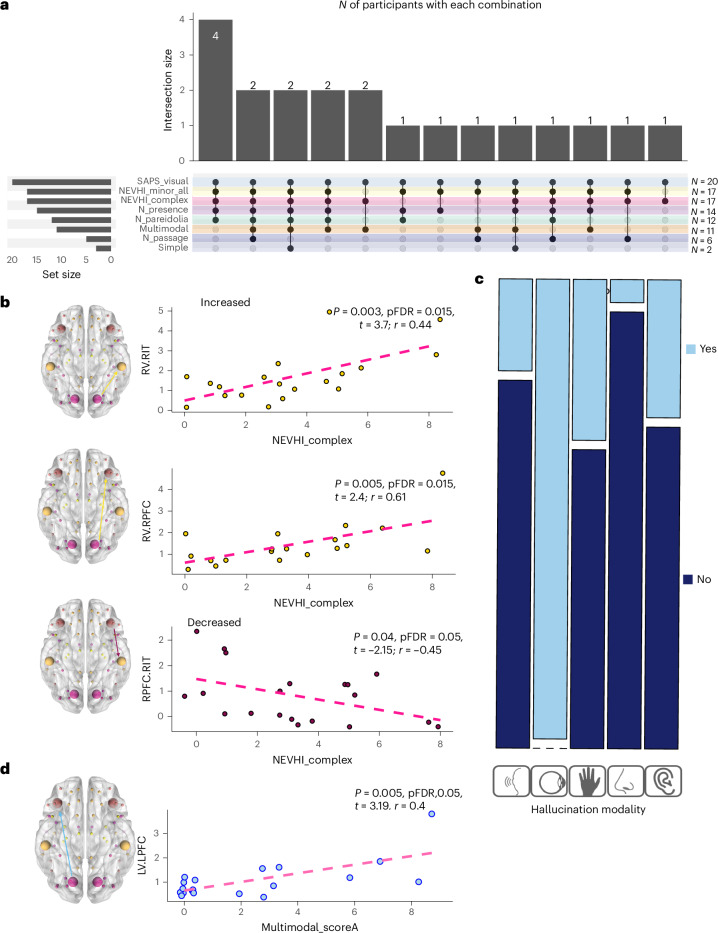
Hallucination phenomenology regression models. **a**, An UpSit plot summarizing the co-occurrence of hallucination subtypes. Bars at the top indicate the number of participants experiencing each specific combination of subtypes (intersection size). Filled dots in the matrix show which subtypes are part of each combination. Bars on the left show the total number of participants with each subtype (set size on the right and actual *N* on the left). Complex, presence, passage, pareidolia and simple VH scores are derived from the NEVHI (described in detail in Supplementary Information 1); multimodal hallucination scores are derived from the SAPS-PD questionnaire described in Supplementary Information 1, and the modality of multimodal hallucination is further subtyped in **c**. All participants with VH were included in the analysis (PD-VH *N* = 20). **b**, Linear regression analyses on CVH for matrix B. Yellow represents increased connectivity in the PEB in PD-VH, and purple represents decreased connectivity. **c**, Multimodal score (derived from SAPS-PD information) regression analyses. The mosaic plot shows the proportion of participants with hallucinations in each specific modality (light blue represents that the symptom is present, dark blue represents that it is not present). Reported in all the plots are the *t* values and the individual regression uncorrected and corrected (FDR) *P* values. Complete details are provided in S7. Yellow is used for regions showing increased connectivity in PD-VH and purple for those showing decreased connectivity in PD-VH. **d**, The matrix A connectivity from left V1 (LV) to left PFC (LPFC) and multimodal hallucinations individual regression. All PD-VH participants were included in the regression analyses (*N* = 20) prior to outlier removal. Source data

The severity of complex VH (CVH) correlated with distinct patterns of connectivity: greater severity was associated with reduced top-down connectivity (right PFC to right ITG) and enhanced bottom-up connectivity (right V1 to right PFC and right ITG). Minor hallucinations (MH) had lower connectivity from left PFC to left V1, although this did not remain significant after correction for multiple comparisons. Multimodality correlates with increased bottom-up connectivity (left V1 to PFC) (see statistics in Fig. 4 and details in Supplementary Information 7).

### Multiple regression models with leave-one-out cross-validation

Both top-down and bottom-up connectivity right-hemisphere changes predicted CVH severity (*R*^2^ = 0.49, adjusted *R*^2^ = 0.4 (3,15) 4.9, *P* value 0.014, root mean square error, 2.26; mean absolute error, 177) (S8). MH were best predicted by left-hemisphere decreases, but these failed to reach significance after further outlier removal in the individual regressions (details reported in Supplementary Information 7). When examining latent activity (matrix A), CVH severity was best predicted by interhemispheric connectivity in V1 and top-down connectivity from PFC; no significant results are found for MH upon outlier removal (S8). Multimodal hallucinations were best predicted by a model using four of the latent matrix A connections found to be altered (details in Supplementary Information 7). Results were stable when introducing MoCA or LEDD as covariates (see details in Supplementary Information 7).

### Neural mass models and simulations

To further explore our results, we extract model-derived activity for each of the three populations’ activity estimated by the ERP neural mass model (spiny stellate cells, inhibitory interneurons and pyramidal neurons) (Supplementary Information 9). We observed that decreased model-derived inhibitory activity estimates across ventral visual and prefrontal regions significantly predicted the severity of CVH (S6); this was also the case when combining inhibitory and excitatory estimates, supporting a network-level deficit and a disruption of excitatory–inhibitory (E–I) balance, with the ITG as a particularly significant contributor (S9).

To probe the network-level implications of the PEB findings, we conducted exploratory simulations of intrinsic inhibitory gain perturbations using the group-level VH effect as a scaling factor (see Methods ‘E–I coupling simulations’ and Supplementary Information 10 for details). This approach allowed us to examine how small changes (0–0.5 and 0–2) in intrinsic coupling would alter cortical population dynamics across the visual hierarchy and to explore whether using the group effect (VH) we could recapitulate our findings, probing possible model-derived estimates of excitatory and inhibitory balance in our groups. State-space area calculations showed divergence between inhibitory interneurons (ii) and pyramidal cells (pyr) in all regions within the first two to six modulation steps, whereas divergence between ii and spiny stellate (ss) cells emerged earlier in the left V1 and right ITG and later (step 7) in the bilateral ITG and left PFC, consistent with hierarchical processing in the ventral visual stream (Supplementary Information 10). Descriptive statistical analyses (Supplementary Information 10) confirmed the robustness of these effects across alternative pipelines: (1) with age as a covariate, (2) when focusing on the entire recording or when focusing on the MMN (100–250 ms) latency and (3) when reducing the perturbation to a smaller interval (0–0.5). We also plot heat maps of simulated peristimulus activity (*x*, time; *y*, inhibitory gain) to visualize deviations around 100–250 ms (MMN latency range) and in the whole task interval, reflecting the simulated increased sensitivity of the ITG circuit to inhibitory perturbations in the VH group. When computing a relative disinhibition index for the MMN latency on relative and comparative (ii versus pyr and ii versus ss) differences, we observed pronounced relative disinhibition in the right ITG and left PFC (all details in Supplementary Information 10; see Supplementary Fig. 9 for a visual summary of the methods and simulation outputs).

#### Dorsal stream model

All details about model specification are reported in Supplementary Information 11. Concerning the task connectivity, the BMA results show that the task effects (rare versus standard) for PD-VH were mostly associated with an altered connectivity between parietal and frontal nodes (pp >0.99). For this model, PD-VH connectivity was increased from left V1 to left IPL, and reduced from IPL to PFC bilaterally; there were no correlations with severity of hallucinations (Supplementary Figs 10–12).

#### Source reconstruction signal and receptor binding atlases exploratory analysis

**Fig. 5:**
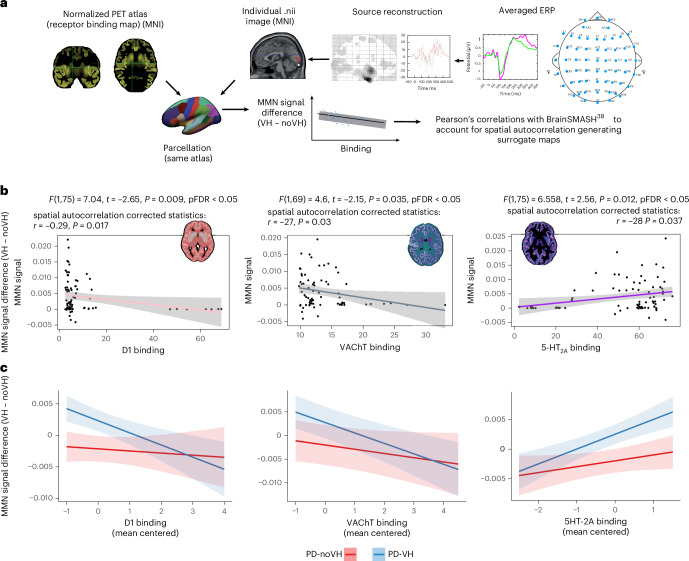
Source-reconstructed signal–receptor binding atlases exploratory analysis. **a**, As described in the Methods and in Supplementary Information 13, we parcellated the normative PET receptor density atlases (maps derived from independent PET datasets, not collected in this cohort) and the source-reconstructed EEG signals with the Freesurfer aparc parcellation inclusive of the Desikan–Killiany atlas (converted to MNI template space); PET atlases and individual participant data were aligned to the brain atlas, and registration was checked examining PET atlas and brain atlas sizes and the transformation matrix, as well as visually with ‘checkreg’, before data extraction. Parcellated data were then used to run linear regression models (one per receptor) on a subset of regions of the Freesurfer aparc parcellation inclusive of the Desikan–Killiany atlas (cerebellar, white matter and ventricular regions were removed) to identify and remove outliers using Cook’s distance. *P* values from the regressions were corrected for multiple comparisons. Then, the BrainSMASH toolbox^38^ was used to generate MNI coordinates for the Desikan–Killiany atlas; we removed the cerebellar and ventricular regions and the regions that were marked as outliers from the regression models before proceeding to compute the Pearson’s actual correlations of the two maps. Only the maps surviving this threshold were finally entered in linear mixed-effects models, which had the purpose of examining individual-level data taking age, LEDD, MoCA score and disease duration into account. **b**, Linear regressions results; the regression lines are color coded to match the color selected for each PET map, for ease of display. Insets: the PET map images were rendered by overlapping the atlas and the relevant PET map; the colors were arbitrarily chosen to highlight the binding distribution in the different maps. The data points in the scatterplot represent the *N* of regions entered in the analysis after nonrelevant regions (cerebellar, white matter and ventricular regions) and outliers were removal. The MMN data presented on the *x* axis represents the mean difference between the MMN signal in PD-VH and PD-noVH. **c**, LMM results. The *x* axis represents mean centered receptor density, and the *y* axis represents the mean centered MMN signal; we mean centered the data as the scale of the different predictors varied greatly. The blue line represents PD-VH, and the red line represents PD-noVH. The shading around the lines represent the s.e.m. Source data

Results for the first region-level linear regression analyses with Cook’s distance^37^ to inspect outliers are reported in Supplementary Information 12 and in Fig. 5. Using BrainSMASH^38^, we confirmed, with a region-level approach, a positive correlation between 5-HT_2A_ regional binding potential (BP_ND_) and MMN source-localized signals (*r* = 0.284, *P* = 0.037), with a higher ‘deviant-standard activity’ in regions of higher BP_ND_. The opposite pattern was observed for dopamine receptor D1 (*r* = −0.29, *P* = 0.017) and vescicular acetilcholine transporter VAChT (*r* = −2.73, *P* = 0.03) maps. D2/D3 was no longer significant after correcting for spatial autocorrelation (*P* > 0.05).

To further investigate the role of covariates, we next fitted a linear mixed-effects model to the region-by-participant dataset, including the main effects of receptor density and VH status as well as their interaction (receptor × VH), with MMN signal as the dependent variable, age, LEDD, sex, disease onset and MoCA entered as covariates, and a random intercept for participant [MMN_signal ~ receptor * VH + age + LEDD + sex + disease onset + MoCA + (1|Participant)]. We found for 5-HT_2A_ a main effect of receptor *t* = 2.13, *P* = 0.034, hallucination status (VH) *t* = 2.52, *P* = 0.017 and a positive receptor × VH interaction between 5-HT_2A_ and VH *t* = 2.06, *P* = 0.040. The same model using D1 receptor availability found a significant VH effect *t* = 2.54, *P* = 0.015, and a negative receptor × VH interaction *t* = –2.13, *P* = 0.033. For VAChT, using the map from *N* = 18 older participants^39^, we found a positive association with receptor (*t* = 1.75, *P* = 0.08) and a negative receptor × VH interaction effect (*t* = −1.58, *P* = 0.1), although neither reached significance; VH status was significant *t* = 2.46, *P* = 0.019. In a sensitivity analysis with the *N* = 5 healthy controls map^40^, we observed the same direction of effects but with a larger effect size and formal significance (partial *r* ≈ –0.037 receptor × VH interaction; Supplementary Information 12). These convergent patterns suggest a negative relationship between VAChT and MMN in the VH group, although not as robust as D1.

No effect of disease duration, LEDD or age was observed (all details and model tables in Supplementary Information 12).

## Discussion

We investigated the neural dynamics underlying sensory processing deficits in people with PD-VH. Using DCM applied to EEG data from a visual MMN task, we showed that people with PD-VH respond differently to the sensory environment and that these differences correlate with their VH.

### Decreased top-down and increased bottom-up activity underlies PD-VH reduction of vMMN

We found that in PD-VH there was a significant reduction of top-down activity and an increase in bottom-up connectivity. Top-down connectivity is crucial for the integration of sensory information^24–26^, and this finding suggests that in PD-VH there is a failure to update predictions based on sensory evidence and an overreliance on or over-response to sensory information during the task. This can contribute to the difficulties in stimulus detection due to retinal degeneration^29,33^ and responding to the changes in the sensory environment. This interpretation resonates with the principles of a recently proposed framework on VH in PD^10^. When taking a hypothesis-free approach, the feedforward model, with a right-hemisphere pattern of increased V1-ITG and V1-PFC connectivity, emerged as the strongest contributor. In line with this result, recent work has shown that people are more likely to hallucinate when sensory information is noisy, especially when combined with strong expectations^41^. Interestingly, this was found with an orientation perception task, consistent with our stimuli, and in the input layers of V2, where orientation-specific activity takes place, lending further support to our interpretation and suggesting that spontaneous feedforward activity in the visual cortex can also lead to hallucinations, rather than feedback activity alone^41^. When further exploring our results using positron emission tomography (PET)-derived normative gradients as biologically informed spatial priors to test whether the pattern of MMN-related alterations aligns with known neurochemical distributions, we found that local source differences were positively correlated with 5-HT_2A_ density. This finding may suggest that this receptor may be facilitating cortical disinhibition, consistent with previous proposals^42,43^ and studies showing 5-HT_2A_ antagonists reducing psychosis-like symptoms^20,42^. Based on our results, we may speculate that this reflects an aberrant or a compensatory excitation. We also find a negative relationship with dopaminergic receptor D1; dopamine is proposed to encode precision weighting^44^. One possible interpretation is that this could bias the system toward assigning undue salience to bottom-up inputs, resulting in illusions and/or hallucinations, as well as disruption of normal mismatch detection.

The analysis of the latent connectivity (A matrix) complements these results, revealing a similar pattern to that seen in the task-modulated model, suggesting that patients with PD-VH not only respond more strongly to sensory information during the visual mismatch negativity (vMMN) task but may do so more generally. The reduced connectivity from the left PFC to the ITG, with increased PFC-V1 connectivity, appears to support this interpretation, as well as previous models of VH in Lewy Body Diseases suggesting a disruption on the object-related semantic network^45^.

### Decreased top-down and increased bottom-up connectivity for vMMN in PD-VH are related to hallucination severity

**Fig. 6:**
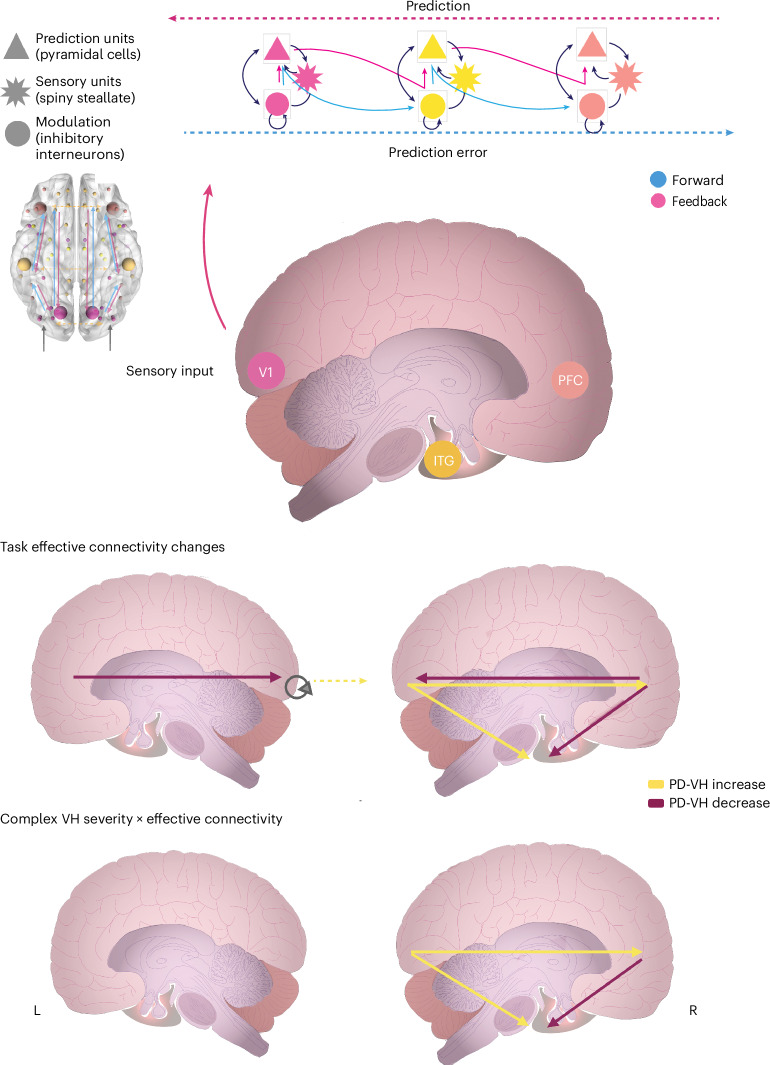
Summary of the neural mass model used in the study and of main results. Top: each node in our network was fitted with the relevant (ERP) neural mass model^69^ in which feedforward connections (blue) are thought to encode prediction errors, whereas feedback connections (pink) encode predictions. Middle: summary of the results of the PEB group analysis for the oddball task eliciting our MMN. Purple arrows are connections where estimated connection strength was decreased in PD-VH, and yellow arrows represent connections where such strength was increased in PD-VH. The gray circle describes the weaker self-inhibition in left V1 in PD-VH. Bottom: summary of the results of multiple regression LOOCV machine learning models relating VH severity to effective connectivity in PD-VH. CVH were best predicted by right-hemisphere connections found related to CVH severity (rV1-rPFC(increased), rV1-rITG (increased) and rPFC-rITG (decreased)), supporting the view that these regions operate as a network. Figure created with Adobe Illustrator for visualization purposes.

Hallucinations severity directly correlated with these connectivity patterns, with pathways of increased connectivity showing a positive relationship and those with decreased connectivity showing a negative relationship. CVH correlated with decreased top-down connectivity from right PFC to ITG, and increased bottom-up connectivity from early visual cortex (V1) to higher-order regions (ITG and PFC) (see Fig. 6 for a visual summary of the model and main results). These results underscore a robust right-hemisphere network impairment associated with complex hallucinations. MH showed a similar but weaker trend predominantly involving left-hemisphere regions, although this did not survive multiple-comparison correction.

A recent DCM fMRI study found that the pattern of altered connectivity observed at rest was predictive of hallucinations severity in PD^8^. When addressing the relationship between multiple connections and hallucinations severity with a similar purpose, we find that CVH were best predicted by right-hemisphere bottom-up and top-down connections, supporting the hypothesis that these regions operate as a network. This right-hemisphere pattern of connections associated with CVH is in line with a recent fMRI study exploring VH severity with resting-state fMRI in PD and DLB with hallucinations, where CVH duration was associated with right hemisphere activity in ventral visual and parieto-occipital regions, and minor visual phenomena to decreased connectivity only in the left hemisphere^30^, as we also found for MH. The result of increased activity from rV1 being associated to VH and VH severity aligns with the finding in schizophrenia of disinhibition in sensory areas (A1) predicting abnormal auditory perception in the aMMN and positive symptoms^24^.

### VH severity may be related to disinhibition

Our PEB analysis revealed reduced self-inhibition in left V1 in PD-VH. Within the DCM framework, decreased self-inhibition represents an increase in excitability and an overweighting of sensory prediction errors^24,46^. This interpretation is in line with accounts of psychosis associated with abnormal gain control in sensory areas of the brain^10,24,45–47^, which is thought to lead to internally generated predictions. In this view, alterations in synaptic gain may represent a transdiagnostic mechanism underlying hallucinatory experiences across different sensory modalities. This aligns also with our result showing a negative correlation between task connectivity and dopaminergic D1 distribution, as dopamine is proposed to encode precision weighting^44^. In our case, in the visual domain, reduced inhibitory activity in early visual cortex may have the effect to render ascending prediction errors less precise, which in turn may bias perception toward top-down priors and facilitate hallucinatory phenomena in PD.

Consistent with this result, our simulations of the effect of VH in response to perturbation of network-level excitatory/inhibitory dynamics recapitulate our results illustrating increased sensitivity in the PD-VH network, particularly in ITG regions, extensively involved in object recognition, particularly of human and animal stimuli^48,49^. Interestingly, human and animal stimuli are often the content of VH in these patients^32,50^. One possible interpretation is that hallucinations may arise from disrupted cortical inhibition and heightened neural sensitivity to perturbations in these object-recognition regions, particularly within the ventral visual stream. Our results lend themselves to more than one potential explanation. First, that of an E–I imbalance similar to what has been proposed in schizophrenia^51^. We can interpret the pattern observed as a reduced adaptability and stability in the network dynamics and in the intrinsic coupling within the regions in our model. This is in line with a recent aMMN study where the electrophysiological patterns observed under ketamine administration and in the relative simulations appear compatible with those observed in psychosis^52^. In this view, reduced activity through glutamate receptors is proposed to induce a decrease in the inhibitory activity of the inhibitory interneurons. Lower GABA+/creatine in the ITG in PD-VH^33^ supports this view. Disinhibition may lead to excessive excitatory signaling, contributing to positive symptoms in schizophrenia, and may also underlie hallucinations in PD-VH. During the task, we observe an excess of excitatory activity in lower visual regions, and in the latent connectivity we observe an overall increase in excitatory activity, consistent with this hypothesis.

Further supporting the interpretation that hallucinations may arise from disrupted cortical inhibition and heightened neural sensitivity to perturbations, exploratory analyses using PET receptor atlases revealed a positive correlation between cortical 5-HT_2A_ receptor distribution and abnormal MMN source activity in PD-VH, consistent with the known role of serotonin in cortical disinhibition^53^ and psychosis^42,43^.

Another possible and complimentary interpretation is that our results may be related to thalamic dysfunction. We did not investigate the thalamus here, as with EEG recordings the signal captures postsynaptic potentials in the cortex, making the choice of using the thalamus as a dipole a challenging one. Nevertheless, a large thalamic cluster was found associated to the task in our source reconstruction analysis. Previous research has shown that the thalamus plays a role in VH in PD^54,55^, and a recent proposal^56^ views the thalamus as a driver of unbalanced network recruitment, suggesting that it may induce decoupling between the default mode network and task-positive networks. This decoupling could disrupt the comparison between priors and sensory percepts, leading to greater reliance on internally generated content.^56^. A recent spectral DCM fMRI study found an involvement of the LGN of the thalamus, together with ventral visual regions, in PD-VH^8^. Despite not being directly comparable to our results owing to the different (resting-state fMRI and task EEG) modalities, nevertheless, when examining the latent activity (between trials) we also have increased top-down connectivity, including from left PFC to V1 as reported in the aforementioned study, and both increased and reduced bottom-up activity.

Our exploratory analysis with PET atlases and source-reconstructed MMN signal also shows a negative relationship between cholinergic receptor distribution and MMN signal in PD-VH. A recent PET study in PD-VH showed a marked cholinergic deficiency in the left ventral visual stream^57^. We may speculate that a dysregulation in the cholinergic system might affect not only cortical areas but also thalamic processing, thereby contributing to abnormal sensory processing.

### Global cognitive decline may modulate the presence of multimodal hallucinations

Our results suggest that multimodal hallucinations may reflect more extensive neural network disruptions, with possible modulation by a subclinical global cognitive decline. When accounting for global cognition, the variance associated with top-down connectivity was largely absorbed, leaving primarily bottom-up sensory pathways significantly linked to multimodal hallucinations. This is consistent with reports of PD-VH declining cognitively more rapidly^2^ and showing possible subclinical cognitive variations early on in their VH^4^.

### Limitations

While our study primarily focused on cortical regions, exploratory analyses hint at the possible involvement of subcortical structures such as the thalamus and cholinergic systems, consistent with previous research, which may offer promising directions for future studies. Limitations include the inability of EEG to robustly assess subcortical sources. In addition, our receptor-binding analyses are entirely exploratory, as we acknowledge that both source reconstruction of EEG signal and receptor density maps have a low spatial resolution. The vAChT map we used was developed on data from both healthy participants and participants with AD; thus, it might partly reflect AD-related patterns. We validated our finding with a sensitivity analysis using another VAChT map (*N* = 5 healthy controls), that by itself would have been a less realistic fit for our study. The value of using these normative receptor maps here lies in leveraging normative gradients as a biologically informed spatial prior, allowing us to test whether the pattern of MMN alterations aligns with known neurochemical distributions. We acknowledge that using individual PET data would be the ideal approach; however, we did not collect PET data, and collecting this type of data from multiple tracers per participant is a highly invasive procedure. We also acknowledge that disease-specific PET maps would add valuable insights, but these are not available in our cohort and rarely available in the field.

The lack of a healthy control sample does not allow us to fully determine whether some of the observed results are specific to PD, and future studies should aim to clarify this issue and disentangle aging-related effects. The main aim of this study was to understand VH in those with PD, and therefore those with PD without VH were deemed the most appropriate comparison group. However, the lack of a sample of healthy controls does not allow us to fully confirm whether results are or are not specifically PD-related. Indeed, healthy controls may also experience MH, even if in a smaller fraction of the cases compared with PD^58^, and disentangling our findings from age-related effects should be addressed in future studies. In addition, a longitudinal approach would allow us to better chart the individual fluctuations, such as daily fluctuations related to medication (off and on phenomenon), or even mood, as it has been observed in Lewy body disease^59^.

## Conclusion

Overall, our findings clarify the top-down and bottom-up neural abnormalities underlying PD-VH, strongly supporting the predictive coding account, and pointing to an alteration in updating predictions based on sensory evidence and a deficit in tracking changes in the sensory environment. Our results highlight potential receptor targets mediating this effect and reinforce the critical role of the ventral visual stream in the generation of VH.

The hyperconnectivity of visual regions strongly contributes to explaining vMMN differences and correlates with hallucinations severity and complexity, supporting targeted therapeutic approaches focused on normalizing sensory processing dynamics. Finally, we show an increasingly extensive network being related to VH complexity and modality and a possible relationship with subclinical cognitive decline. Having provided a mechanistic model of predictive coding deficits in PD-VH, we suggest that future longitudinal studies will be essential to confirm whether observed connectivity changes directly underpin hallucinations alone or reflect a broader underlying cognitive decline.

## Methods

### Participants

The study received ethics approval from London Camberwell St Giles REC (18/LO/2144). We enrolled 18 people (6 female) with PD without hallucinations (PD-noVH) and 20 people (7 female) meeting criteria for Parkinson’s psychosis (PD-VH in this Article)^60^. The study was registered on clinicaltrials.gov (ID NCT03661125). Informed consent was obtained by participants before beginning the screening visit. Participants received £75 in compensation for their time and were offered lunch and a taxi for travel to and from the facility. Parkinson’s clinical severity was assessed by specialist motor disorders neurologists (see Supplementary Information 1 for details and tables), and motor impairments were assessed using the SCOPA-motor scale^61^. Participant recruitment and screening details are provided in detail in Supplementary Information 1 and in our ERP study^20^.

VH and psychosis in PD-VH were further assessed using the Scale for the Assessment of Positive Symptoms-PD (SAPS-PD)^62^, and an expanded version of the North-East Visual Hallucination Interview (NEVHI)^63^ to examine the phenomenology of VH and their subtypes in PD-VH (Supplementary Information 1). In brief, the NEVHI is a semi-structured interview that allows one to compute severity scores for each hallucination subtype, focusing on the most recent experiences (that is, asking the date when the latest experience occurred, how many times it occurred in the previous two weeks and how often this happens in a typical month). In our study, we computed a continuous severity score by multiplying the time (number of minutes) spent hallucinating by the number of hallucinatory experiences, for each subtype, in a month^20^. While this approach does not allow one to fully account for the individual fluctuations of the symptoms, by asking the number of a specific type experience in the past month, in the past 2 weeks and the relative duration of each experience and whether it happens repeatedly throughout the day, it allows one to factor in individual differences more efficiently than with other questionnaires.

### Task and EEG recording

We used a visual MMN task whereby the stimulus frequency variation was the orientation of peripheral bars (S2). Figure 1a provides a visual summary of the vMMN task; the task design was inspired by vMMN paradigms proposed in Qian et al.^64^; additional details are provided in S2, and the task pilot and specific ERP analysis is described in detail by Vignando et al.^20^. The visual MMN task (programmed with Presentation v.17.2) presented pseudo-randomized stimuli. The peripheral stimuli presented to elicit the vMMN task comprised symmetrical black bars on a light-gray background. The ‘oddball’ variation designed to elicit the MMN was in orientation: bars are presented with a 90 (deviant), 130 (frequent deviant) and 160 (standard) degree orientation. The bars for each orientation were presented bilaterally and simultaneously in the visual field (offset 400/−400) centered on the horizontal meridian and remained on the screen for 50 ms. The cross-changes (*N*_trials_ = 22) had a duration of 200 ms. The stimulus-onset asynchrony was 600 ms. The task consisted of 1,000 trials, with 640 standard trials, 240 frequent deviant trials and 120 rare deviant trials. Here, we focus on the rare deviant and the standard trials. The explicit task required participants to press a button when the cross at the center of the screen became bigger and another button when the cross became smaller. The cross-change appeared in 22 trials. When performing the task, participants were seated at approximately 70 cm from the cathod ray tube screen where the visual stimuli were being presented. Before each recording, impedance was kept ≤11 kΩ.

### EEG data preprocessing

EEG data were preprocessed in SPM12 using the standard ERP pipeline for MMN data^14^ (Supplementary Information 2). Continuous data were converted and re-referenced using a custom average-reference montage that excluded horizontal and vertical electro-oculogram (HEO/VEG) channels. Data were high-pass filtered at 0.3 Hz (fifth-order Butterworth, two-pass), downsampled to 500 Hz and low-pass filtered at 30 Hz (fifth-order Butterworth, two-pass). Epochs were defined from –100 to 500 ms around stimulus onset for standard and rare deviant conditions (shift triggers = 0) using a trial event file. Baseline correction was applied using the –100 to 0 ms prestimulus interval. Artifactual trials and channels were identified by visual inspection using the FieldTrip toolbox^65^ and automatically rejected using an 80 µV threshold. The *N* = 22 trials where participants were asked to press a button to respond to the change in size of the fixation cross were removed to avoid movement artifacts. The remaining trials were averaged separately for standard and deviant conditions before source reconstruction (Supplementary Information 2–4).

### Sensor space and source reconstruction analysis

We conducted analyses in sensor space to identify the electrodes where the ERP amplitude differed across conditions during the task and to validate the results from our ERP study^20^ (Supplementary Information 3). To gain more detailed spatial information regarding the source of our signal, we perform source reconstruction (S4) for the 60–400 ms interval of interest to be investigated with the DCM analyses, and we entered them in second-level analyses (one-sample *t*-test; paired *t*-test) to investigate the neural correlates of task-related activity. The scope of this step was to guide, in a principled way, the choice of the dipoles and specifically to confirm our original choice to focus on ventral visual regions. DCM inferences were made at the level of macrosources and effective connectivity; precise centimeter-scale localization was not the goal.

We focus on a wide interval (60–400 ms) rather than a narrower one where MMN usually peaks (for example, 100–180 ms and 100–250 ms) for several reasons. First, this approach avoids restricting the analysis to a single level of the hierarchy^66^ and allows us to capture both early MMN activity and later components such as the P300 (300–400 ms), which are thought to be modulated by probability as well at a higher level (involuntary attention) of the hierarchy and which in our ERP study were observed in both groups (even if not surviving multiple-comparisons correction in PD-VH)^20^. Second, this approach provides sufficient data for source reconstruction to guide the specification of dipoles for the generative models. Third, this approach leverages DCM’s ability to provide an explanation of the complex neural dynamics generating the EEG signals of interest. As DCM relies on biologically grounded models to investigate how EEG signals are generated rather than summarizing and averaging evoked responses of images^12,67^, choosing a wider interval was in the interest of capturing the earlier and later dynamics associated to our task. The 60-ms lower bound reflects previous findings that visual information can reach the primary visual cortex as early as ~60 ms after stimulus onset^68^.

### Individual DCM model specification

Based on the results of the ERP study and of the source reconstruction analyses, pointing to the involvement of occipital, occipito-parietal and frontal regions, we decided to investigate the ventral and the dorsal pathways separately. For the ventral pathway, we designed three models; all three share the same dipoles to allow for Bayesian model selection, but differ in their neuronal models (Supplementary Fig. 3). As we did not have strong results for the dorsal pathway in the source reconstruction analysis we focused on one dorsal model, using source reconstruction results, and the literature on the Benton line judgment task and the neural anatomy of the dorsal pathway^34^ to define the dipoles (Supplementary Information 11). We used an ERP neural mass model^69,70^, with eight spatial modes, focusing on the 0–400 ms interval, rare deviant versus standard. An equivalent current dipole was used for each of the six sources, with anterior occipital, inferior temporal and dorsolateral prefrontal regions, bilaterally (Fig. 2c). All model specification details are provided in S5, Supplementary Figs. 3 and 11, and Bayesian model comparisons are detailed in Supplementary Fig. 4.

### E–I coupling simulations

We conducted this exploratory analysis to probe the network-level mechanisms and to understand more clearly the role of intrinsic coupling in the results pertaining to the previous sections and on the PEB analysis where specific parameter changes were found to be associated with intrinsic coupling. The aim was to modulate the intrinsic coupling parameter and examine how connectivity changes with VH. We did so by adapting and tailoring the methods and procedures described by Rosch et al.^52^ to our group contrast and our neural mass model (ERP^69^; see all details in Supplementary Information 10). In brief, we modulated the intrinsic coupling parameter to simulate the graded effects of VH on our cortical neural mass models. We started from the grand mean model and individual participants inversions and applied VH-related perturbations estimated from the PEB analysis. We extracted the VH contrast from the design matrix, and scaled the G parameter across ten steps, using two ranges: (i) 0–2 (ref. ^52^) and (ii) 0–0.5. The latter was chosen as a sensitivity analysis to probe smaller perturbations, as our aim was not to simulate pharmacological modulation^52^ but to explore more subtle effects. Both analyses were repeated with and without age as a covariate. These manipulations were applied to all six cortical regions in the model (bilateral V1, ITG and PFC), with each step incrementally increasing the strength of intrinsic coupling of each cortical source proportionally to the magnitude of the PEB-derived group difference. The purpose was to simulate biologically and physiologically plausible perturbations to explore how network dynamics evolve under increasing VH load.

### Exploratory receptor binding atlases and MMN source-reconstructed signal analysis

The aim of this analysis was to test, in an exploratory and hypothesis-generating way, whether EEG activity during the vMMN task relates to the distribution of neurotransmitter receptors implicated in PD with VH, namely the serotonergic^42,43^, dopaminergic^71^ and cholinergic^57^ systems. No PET data were collected in this study. Instead, we used normative receptor density maps (receptor atlases)^39,40,72–78^, which are constructed from independent PET datasets to provide population-level distributions of receptor and transporter density across the cortex^73,75,78^. These maps can be applied analogously to the Allen Brain Atlas for gene expression and have been shown to align with structural connectivity and neurophysiological dynamics^73,75^. The analysis was not intended to provide precise spatial localization (which EEG does not afford), but to probe whether variance in task-related EEG activity showed systematic associations with the known distribution of neurotransmitter systems, thereby informing hypotheses for future studies using individual PET or higher-resolution modalities. For each participant, we computed vMMN difference images (rare deviant – standard) from the source-reconstructed EEG signal. The Freesrufer aparc parcellation was derived from Desikan–Killiany^79^ and aseg atlas^80^ and supplemented by large subcortical structures (hippocampus, thalamus, amygdala and basal ganglia; all other subcortical structures such as vessels, ventricles and cerebellar structures were removed). The atlas was applied to each source-localized MMN NIfTI image using the imcalc function in SPM with the expression i1.* (i2 > 0) expression to ensure only values inside the atlas space were retained. The same atlas was applied to both the source-localized MMN maps and the PET maps after alignment into a common space. Figures showing PET maps and atlas overlap are provided in Supplementary Information 12. A custom script was created to parcellate the source-reconstructed difference images and the PET maps of interest (11c-lsn3172176^76^ for muscarinic M1, [^18^F]altanserin for 5-HT_2A_^74^, [^18^F]FEOBV^39,40^ for VAChT, [^18^F]fallypride^72^ for dopamine D2 receptors and [^11^C]SCH23390^77^ for dopamine D1 receptors), to ensure correct alignment of PET maps and EEG data. This enabled the extraction of regional binding potential (BP_ND_) for each PET map and the corresponding reconstructed EEG signal within the regions defined by the atlas. See further details in S12. The statistical analysis performed on these data is described in ‘Statistical analysis’.

### Statistical analysis

#### Participant demographics

We carried out a one-way ANOVA to test whether our participants differed in age, disease duration, motor symptoms, LEDD and MoCA score and a chi-square test to ensure that PD-noVH and PD-VH had an equal number of male and female participants. We also ran a Pearson’s product moment correlational analysis between the clinical variables to test whether hallucination severity correlated with any of the clinical variables (Supplementary Tables 1–4 and Supplementary Fig. 1).

#### Cross detection task performance

We previously^20^ investigated task performance using a one-way ANOVA, confirming that participants of both were paying attention to the screen and executing the task (S2). We also compute reaction times using a two-tailed Mann–Whitney *U* test (Supplementary Information 2).

#### Second-level analysis with PEB

After specifying individual DCMs for each participant, group-level inference was performed using PEB^81,82^. PEB uses a Bayesian linear regression approach, which allows group-level comparisons of modulatory parameters estimated at the individual level, without losing information about the precision of those estimations. We fitted a general linear model to the connection strengths estimated with the first level analyses and with covariates: mean, group (PD-VH (-1) or PD-noVH (1)); we repeated the same analysis including also age to explore possible ageing-related effects. spm_dcm_bmr and spm_dcm_peb were used to perform Bayesian model reduction (BMR) and to generate model posteriors, respectively. BMR allows a hypothesis-free approach to finding the winning model for effective connectivity within a network. We used Bayesian model averaging using spm_dcm_bma to investigate group-level differences between participants with and without VH. We analyzed the B (task modulatory) matrix and A (latent connectivity) matrix (feedforward, feedback and interhemispheric connectivity). As PD-VH were coded as −1 and PD-noVH as 1, Ep <0 for extrinsic connectivity describes stronger estimates for PD-VH, and Ep >0 stronger estimates for PD-noVH. Self-inhibition is an intrinsic parameter, and it is a negative parameter. As these are intrinsic values, the parameters are log-scaled measures of inhibition; thus, Ep <0 in this case describes less negative self-inhibition/disinhibition.

This analysis was followed by recursive model comparison (recursive PEB) to formally identify the network parameters more strongly contributing to group differences^52^. We defined a model space including forward, backward, self-inhibitory, intrinsic coupling and time constraints parameter sets. For each model, the log model evidence (free energy) was computed and compared using Bayesian model comparison at each set of parameters level (pp >0.99). This approach allows us to switch the effect of group on and off for specific parameters (see further details in Supplementary Information 6). This procedure balances model accuracy and complexity, identifying the most parsimonious explanation of between-group effects while controlling for overfitting. The parametrized winning second-level model was further examined with Bayesian model reduction.

We also explored task connectivity in PD-noVH to isolate the effect of task, as we previously showed that this group had a stronger MMN if compared with PD-VH (Supplementary Fig. 6). We also explored potential group differences in intrinsic coupling as a robustness and mechanistic check for our simulation framework (described below). Results are reported in Supplementary Fig. 9.

#### Linear regression analyses of individual connections by hallucination subtype

We conducted linear regression analyses between NEVHI scores for visual complex (CVH) and minor (MH) hallucinations (for the PD-VH group) and the estimated connection strength (Ep) for each of the connections found (pp >0.99) with the PEB analysis. As we also had collected information on multimodality of hallucinations in our participants from the SAPS-PD (Supplementary Information 1), we explored the relationship between connectivity in PD-VH and multimodality of hallucinations. Outliers were identified using Cook’s distance^37^ and removed. We computed multiple-comparisons correction using the false discovery rate (FDR) across all individual models run. R packages Tidyverse^83^ and dplyr^84^ were used for these analyses (Supplementary Information 7).

#### Leave one out multiple regression models by hallucination subtype

As we found more than one connection correlating with the severity of VH, we performed post-hoc backward regression analyses with leave one out cross-validation (using R package caret^85^). We also ran models including only the individual connections found with the individual regression analyses described in the previous paragraph to explore whether the model with the multiple connections was improved for predicting simple and complex VH (severity scores) (separately). We used the Bayesian information criterion to select the best model and avoid overfitting. We also computed the same models with MoCA and then LEDD as covariates to accommodate the potential role of general cognition and medication (Supplementary Information 8).

#### Analysis of neural sources

We explored the model-derived hidden-state trajectories of the three neural populations described in the ERP neural mass model (spiny stellate cells, inhibitory interneurons and pyramidal neurons) by retrieving the model-derived values from the fitted ERP neural mass model, obtained from the DCM generative model using the posterior parameter estimates. All details in Supplementary Information 9.

#### Linear regressions and LMM for exploratory receptor binding atlases and MMN source-reconstructed signal analysis

To explore whether the spatial pattern of the EEG signal associated to our MMN task aligned with dopamine, serotonin and acetylcholine receptor density in participants with VH, we carried out regression and correlational analyses between individual source localized data for deviant-standard EEG signal maps and receptor density maps, as detailed in the Methods (‘Exploratory receptor binding atlases and MMN source reconstructed signal analysis’) and Supplementary Information 12. First, we ran individual regression models using regional receptor binding as a predictor and regional MMN signal difference (VH – noVH) as the dependent variable. Outliers were identified with Cook’s distance^37^ and removed (see details and results in Supplementary Information 12). *P* values for these models were corrected for multiple comparisons. We proceeded with further analysis including only the maps surviving multiple comparisons correction. We accounted for spatial autocorrelation using the BrainSMASH toolbox^38^ to generate the MNI (Montreal Neurological Institute) coordinates for our atlas. We excluded the outlier regions previously identified in the regression models to ensure that the correlational analyses were performed on the same regions used in the regressions. By creating centroids from the NIfTI (.nii) atlas and running multiple permutations, we obtained a correlation coefficient that is proposed, by Burt and colleagues^38^, to reflect the relationship between the two maps free from spatial autocorrelation. For the maps surviving this ‘actual’ correlational analysis, we explore the possible role of relevant demographics and clinical covariates fitting a linear mixed-effects model (LMM) to the dataset reshaped in a region-by-subject format. We used lme4^86^ and lmerTest^87^ to fit the LMM including the main effects of receptor density and VH status as well as their interaction (receptor × VH), and including age, LEDD, sex, disease onset and MoCA as covariates, and a random intercept for participant [MMN_signal~_receptor * VH + age + LEDD + sex + disease onset + MoCA + (1 | Participant)]. The term receptor × VH expands to both interaction and main effects (receptor + VH + receptor:VH); thus, the model includes both main effects (receptor and VH) and their interaction, with additional methodological and model details provided in Supplementary Information 12.

### Reporting summary

Further information on research design is available in the Nature Portfolio Reporting Summary linked to this article.

## Supplementary information

Supplementary Information Supplementary Information Supplementary Figs. 1–15 and Tables 1–7.

Reporting Summary

Peer Review File

## Source data

Source Data Fig. 1 Source data.

Source Data Fig. 2 Source data.

Source Data Fig. 3 Source data.

Source Data Fig. 4 Source data.

Source Data Fig. 5 Source data.

## Data Availability

The data supporting the analyses presented in the main text is openly available from the King’s College London research data repository, KORDS, at 10.18742/32205198, including clinical and demographic data and connection strengths for all the connections in the models. Raw EEG files will be shared upon completing a data sharing agreement. All PET maps are available via GitHub at https://github.com/netneurolab/neuromaps and https://github.com/juryxy/JuSpace. To request the raw data, contact the corresponding author.

## Code availability

All the code used in the study is available via GitHub at https://github.com/VMiri/visual-MMN-DCM- and on the study OSF page (https://osf.io/umd89).

## References

[1] Schapira, A. H., Chaudhuri, K. R. & Jenner, P. Non-motor features of Parkinson disease. *Nat. Rev. Neurosci.* **18**, 435–450 (2017). 28592904 10.1038/nrn.2017.62

[2] Ffytche, D. H. et al. The psychosis spectrum in Parkinson disease. *Nat. Rev. Neurol.* **13**, 81–95 (2017). 28106066 10.1038/nrneurol.2016.200 PMC5656278

[3] Pisani, S. et al. Parkinson’s disease psychosis associated with accelerated multidomain cognitive decline. *BMJ Ment. Health* **27**, 1–10 (2024). 39043465 10.1136/bmjment-2024-301062 PMC11268075

[4] Stampacchia, S. et al. Connectome-based brain fingerprints predict early cognitive decline in Parkinson’s patients with minor hallucinations. Preprint at *bioRxiv*10.1101/2025.04.17.649310 (2025).

[5] Vignando, M. et al. Mapping brain structural differences and neuroreceptor correlates in Parkinson’s disease visual hallucinations. *Nat. Commun.* **13**, 519 (2022). 35082285 10.1038/s41467-022-28087-0 PMC8791961

[6] Weil, R. S., Hsu, J. K., Darby, R. R., Soussand, L. & Fox, M. D. Neuroimaging in Parkinson’s disease dementia: connecting the dots. *Brain Commun.* **1**, fcz006 (2019). 31608325 10.1093/braincomms/fcz006 PMC6777517

[7] Lenka, A., Jhunjhunwala, K. R., Saini, J. & Pal, P. K. Structural and functional neuroimaging in patients with Parkinson’s disease and visual hallucinations: a critical review. *Parkinsonism Relat. Disord.* **21**, 683–691 (2015). 25920541 10.1016/j.parkreldis.2015.04.005

[8] Thomas, G. E. et al. Changes in both top-down and bottom-up effective connectivity drive visual hallucinations in Parkinson’s disease. *Brain Commun.* **5**, fcac329 (2023). 36601626 10.1093/braincomms/fcac329 PMC9798302

[9] Shine, J. M. et al. Abnormal connectivity between the default mode and the visual system underlies the manifestation of visual hallucinations in Parkinson’s disease: a task-based fMRI study. *npj Parkinsons Dis.* **1**, 15003 (2015). 28725679 10.1038/npjparkd.2015.3 PMC5516559

[10] Collerton, D. et al. Understanding visual hallucinations: a new synthesis. *Neurosci. Biobehav. Rev.* **150**, 105208 (2023). 37141962 10.1016/j.neubiorev.2023.105208

[11] Sterzer, P. et al. The predictive coding account of psychosis. *Biol. Psychiatry* **84**, 634–643 (2018). 30007575 10.1016/j.biopsych.2018.05.015 PMC6169400

[12] Bastos, A. M. et al. Canonical microcircuits for predictive coding. *Neuron* **76**, 695–711 (2012). 23177956 10.1016/j.neuron.2012.10.038 PMC3777738

[13] Haarsma, J. et al. Influence of prior beliefs on perception in early psychosis: effects of illness stage and hierarchical level of belief. *J. Abnorm. Psychol.* **129**, 581 (2020). 32757602 10.1037/abn0000494 PMC7409392

[14] Garrido, M. I., Kilner, J. M., Stephan, K. E. & Friston, K. J. The mismatch negativity: a review of underlying mechanisms. *Clin. Neurophysiol.* **120**, 453–463 (2009). 19181570 10.1016/j.clinph.2008.11.029 PMC2671031

[15] Bhat, A. et al. Transcriptome-wide association study reveals two genes that influence mismatch negativity. *Cell Rep.* **34**, 108868 (2021). 33730571 10.1016/j.celrep.2021.108868 PMC7972991

[16] Friston, K. J. & Dolan, R. J. Computational and dynamic models in neuroimaging. *NeuroImage* **52**, 752–765 (2010). 20036335 10.1016/j.neuroimage.2009.12.068 PMC2910283

[17] Feldman, H. & Friston, K. J. Attention, uncertainty, and free-energy. *Front. Hum. Neurosci.* **4**, 215 (2010). 21160551 10.3389/fnhum.2010.00215 PMC3001758

[18] Friston, K. J. Hallucinations and perceptual inference. *Behav. Brain Sci.* **28**, 764–766 (2005).

[19] Rao, R. P. & Ballard, D. H. Predictive coding in the visual cortex: a functional interpretation of some extra-classical receptive-field effects. *Nat. Neurosci.* **2**, 79–87 (1999). 10195184 10.1038/4580

[20] Vignando, M. et al. Visual mismatch negativity in Parkinson’s psychosis and potential for testing treatment mechanisms. *Brain Commun.* **6**, fcae291 (2024). 39355002 10.1093/braincomms/fcae291 PMC11443450

[21] Kiebel, S. J., Garrido, M. I., Moran, R. J. & Friston, K. J. Dynamic causal modelling for EEG and MEG. *Cogn. Neurodyn.* **2**, 121–136 (2008). 19003479 10.1007/s11571-008-9038-0 PMC2427062

[22] Kiebel, S. J., David, O. & Friston, K. J. Dynamic causal modelling of evoked responses in EEG/MEG with lead field parameterization. *NeuroImage* **30**, 1273–1284 (2006). 16490364 10.1016/j.neuroimage.2005.12.055

[23] Friston, K. A theory of cortical responses. *Philos. Trans. R. Soc. B* **360**, 815–836 (2005). 10.1098/rstb.2005.1622 PMC1569488 15937014

[24] Adams, R. A. et al. Computational modeling of electroencephalography and functional magnetic resonance imaging paradigms indicates a consistent loss of pyramidal cell synaptic gain in schizophrenia. *Biol. Psychiatry* **91**, 202–215 (2022). 34598786 10.1016/j.biopsych.2021.07.024 PMC8654393

[25] Summerfield, C. & Egner, T. Expectation (and attention) in visual cognition. *Trends Cogn. Sci.* **13**, 403–409 (2009). 19716752 10.1016/j.tics.2009.06.003

[26] Kveraga, K. & Bar, M. Top-down predictions in the cognitive brain. *Brain Cognit.* **94**, 65–67 (2014). 10.1016/j.bandc.2007.06.007 PMC2099308 17923222

[27] Shine, J. M., O’Callaghan, C., Halliday, G. M. & Lewis, S. J. Tricks of the mind: visual hallucinations as disorders of attention. *Prog. Neurobiol.* **116**, 58–65 (2014). 24525149 10.1016/j.pneurobio.2014.01.004

[28] Pagonabarraga, J. et al. Neural correlates of minor hallucinations in non-demented patients with Parkinson’s disease. *Parkinsonism Relat. Disord.* **20**, 290–296 (2014). 24373690 10.1016/j.parkreldis.2013.11.017

[29] Diederich, N. J., Fénelon, G., Stebbins, G. & Goetz, C. G. Hallucinations in Parkinson disease. *Nat. Rev. Neurol.* **5**, 331–342 (2009). 19498436 10.1038/nrneurol.2009.62

[30] D’Antonio, F. et al. Visual hallucinations in Lewy body disease: pathophysiological insights from phenomenology. *J. Neurol.* **269**, 3636–3652 (2022). 35099586 10.1007/s00415-022-10983-6 PMC9217885

[31] Montagnese, M., Vignando, M. & Mehta, M. A. Cognitive and visual processing performance in Parkinson’s disease patients with vs without visual hallucinations: a meta-analysis. *Cortex* **146**, 161–172 (2022). 34864505 10.1016/j.cortex.2021.11.001

[32] Pagonabarraga, J., Bejr-Kasem, H., Martinez-Horta, S. & Kulisevsky, J. Parkinson disease psychosis: from phenomenology to neurobiological mechanisms. *Nat. Rev. Neurol.* **20**, 135–150 (2024). 38225264 10.1038/s41582-023-00918-8

[33] Firbank, M. J. et al. Reduced occipital GABA in Parkinson disease with visual hallucinations. *Neurology* **91**, e675–e685 (2018). 30021920 10.1212/WNL.0000000000006007 PMC6105043

[34] Garcia-Diaz, A. I. et al. Structural brain correlations of visuospatial and visuoperceptual tests in Parkinson’s disease. *J. Int. Neuropsychol. Soc.* **24**, 33–44 (2018). 28714429 10.1017/S1355617717000583 PMC5851059

[35] Weil, R. S. et al. Visual dysfunction in Parkinson’s disease. *Brain* **139**, 2827–2843 (2016). 27412389 10.1093/brain/aww175 PMC5091042

[36] Bönstrup, M., Schulz, R., Feldheim, J., Hummel, F. C. & Gerloff, C. Dynamic causal modelling of EEG and fMRI to characterize network architectures in a simple motor task. *NeuroImage* **124**, 498–508 (2016). 26334836 10.1016/j.neuroimage.2015.08.052

[37] Venables, W. N. & Ripley, B. D. *Modern Applied Statistics with S* 4th edn (Springer, 2002).

[38] Burt, J. B., Helmer, M., Shinn, M. W., Anticevic, A. & Murray, J. D. Generative modeling of brain maps with spatial autocorrelation. *NeuroImage* **220**, 117038 (2020). 32585343 10.1016/j.neuroimage.2020.117038

[39] Aghourian, M. et al. Quantification of brain cholinergic denervation in alzheimer’s disease using PET imaging with [18 f]-feobv. *Mol. Psychiatry* **22**, 1531–1538 (2017). 28894304 10.1038/mp.2017.183

[40] Bedard, M.-A. et al. Brain cholinergic alterations in idiopathic rem sleep behaviour disorder: a PET imaging study with ^18^F-FEOBV. *Sleep Med.* **58**, 35–41 (2019). 31078078 10.1016/j.sleep.2018.12.020

[41] Haarsma, J., Deveci, N., Corbin, N., Callaghan, M. F. & Kok, P. Expectation cues and false percepts generate stimulus-specific activity in distinct layers of the early visual cortex. *J. Neurosci.* **43**, 7946–7957 (2023). 37739797 10.1523/JNEUROSCI.0998-23.2023 PMC10669763

[42] Huot, P. et al. Increased 5-HT2A receptors in the temporal cortex of parkinsonian patients with visual hallucinations. *Mov. Disord.* **25**, 1399–1408 (2010). 20629135 10.1002/mds.23083

[43] Ballanger, B. et al. Serotonin 2A receptors and visual hallucinations in Parkinson disease. *Arch. Neurol.* **67**, 416–421 (2010). 20385906 10.1001/archneurol.2010.35

[44] Parr, T., Benrimoh, D. A., Vincent, P. & Friston, K. J. Precision and false perceptual inference. *Front. Integr. Neurosci.* **12**, 39 (2018). 30294264 10.3389/fnint.2018.00039 PMC6158318

[45] Tsukada, H., Fujii, H., Aihara, K. & Tsuda, I. Computational model of visual hallucination in dementia with Lewy bodies. *Neural Netw.* **62**, 73–82 (2015). 25282547 10.1016/j.neunet.2014.09.001

[46] Ranlund, S. et al. Impaired prefrontal synaptic gain in people with psychosis and their relatives during the mismatch negativity. *Hum. Brain Mapp.* **37**, 351–365 (2016). 26503033 10.1002/hbm.23035 PMC4843949

[47] Friston, K. J., Parr, T. & de Vries, B. The graphical brain: belief propagation and active inference. *Netw. Neurosci.* **1**, 381–414 (2017). 29417960 10.1162/NETN_a_00018 PMC5798592

[48] Kanwisher, N., McDermott, J. & Chun, M. M. The fusiform face area: a module in human extrastriate cortex specialized for face perception. *J. Neurosci.* **17**, 4302–4311 (1997). 9151747 10.1523/JNEUROSCI.17-11-04302.1997 PMC6573547

[49] Giussani, C. et al. Anatomical correlates for category-specific naming of living and non-living things. *NeuroImage* **56**, 323–329 (2011). 21296167 10.1016/j.neuroimage.2011.01.080

[50] Mosimann, U. P. et al. Characteristics of visual hallucinations in Parkinson disease dementia and dementia with Lewy bodies. *Am. J. Geriatr. Psychiatry* **14**, 153–160 (2006). 16473980 10.1097/01.JGP.0000192480.89813.80

[51] Liu, Y. et al. A selective review of the excitatory–inhibitory imbalance in schizophrenia: underlying biology, genetics, microcircuits, and symptoms. *Front. Cell Dev. Biol.* **9**, 664535 (2021). 34746116 10.3389/fcell.2021.664535 PMC8567014

[52] Rosch, R. E., Auksztulewicz, R., Leung, P. D., Friston, K. J. & Baldeweg, T. Selective prefrontal disinhibition in a roving auditory oddball paradigm under *N*-methyl-D-aspartate receptor blockade. *Biol. Psychiatry Cogn. Neurosci. Neuroimaging* **4**, 140–150 (2019). 30115499 10.1016/j.bpsc.2018.07.003 PMC6374982

[53] Carhart-Harris, R. L. & Nutt, D. J. Serotonin and brain function: a tale of two receptors. *J. Psychopharmacol.* **31**, 1091–1120 (2017). 28858536 10.1177/0269881117725915 PMC5606297

[54] Ignatavicius, A., Matar, E. & Lewis, S. J. Visual hallucinations in Parkinson’s disease: spotlight on central cholinergic dysfunction. *Brain* **148**, 376–393 (2025). 39252645 10.1093/brain/awae289 PMC11788216

[55] Zarkali, A., McColgan, P., Leyland, L. A., Lees, A. J. & Weil, R. S. Longitudinal thalamic white and grey matter changes associated with visual hallucinations in Parkinson’s disease. *J. Neurol. Neurosurg. Psychiatry* **93**, 169–179 (2022). 34583941 10.1136/jnnp-2021-326630 PMC8785065

[56] Onofrj, M. et al. The central role of the Thalamus in psychosis, lessons from neurodegenerative diseases and psychedelics. *Transl. Psychiatry* **13**, 384 (2023). 38092757 10.1038/s41398-023-02691-0 PMC10719401

[57] d’Angremont, E. et al. Cholinergic deficiency in Parkinson’s disease patients with visual hallucinations. *Brain* **147**, 3370–3378 (2024). 38864492 10.1093/brain/awae186 PMC11449127

[58] Pagonabarraga, J. et al. Minor hallucinations occur in drug-naive Parkinson’s disease patients, even from the premotor phase. *Mov. Disord.* **31**, 45–52 (2016). 26408291 10.1002/mds.26432

[59] Watanabe, H. et al. Negative mood invites psychotic false perception in dementia. *PLoS ONE* **13**, e0197968 (2018). 29856844 10.1371/journal.pone.0197968 PMC5983458

[60] Ravina, B. et al. Diagnostic criteria for psychosis in Parkinson’s disease: report of an NINDS, NIMH work group. *Mov. Disord.* **22**, 1061–106 (2007). 17266092 10.1002/mds.21382

[61] Marinus, J. et al. A short scale for the assessment of motor impairments and disabilities in Parkinson’s disease: the SPES/SCOPA. *J. Neurol. Neurosurg. Psychiatry* **75**, 388–395 (2004). 14966153 10.1136/jnnp.2003.017509 PMC1738938

[62] Voss, T. et al. Performance of a shortened Scale for Assessment of Positive Symptoms for Parkinson’s disease psychosis. *Parkinsonism Relat. Disord.* **19**, 295-9 (2013). 23211417 10.1016/j.parkreldis.2012.10.022

[63] Mosimann, U. P. et al. A semi-structured interview to assess visual hallucinations in older people. *Int. J. Geriatr. Psychiatry* **23**, 712–718 (2008). 18181237 10.1002/gps.1965

[64] Qian, X. et al. The visual mismatch negativity (vMMN): toward the optimal paradigm. *Int. J. Psychophysiol.* **93**, 311–315 (2014). 24933413 10.1016/j.ijpsycho.2014.06.004

[65] Oostenveld, R., Fries, P., Maris, E. & Schoffelen, J.-M. FieldTrip: Open source software for advanced analysis of MEG, EEG, and invasive electrophysiological data. *Comput. Intell. Neurosci.* **1**, 156869 (2011). 10.1155/2011/156869 PMC3021840 21253357

[66] Mancini, V. & Nour, M. M. If mismatch negativity is the answer, what is the question? On the nature of predictive coding abnormalities in psychosis. *Biol. Psychiatry Glob. Open Sci.* **5**, 100412 (2025). 40018661 10.1016/j.bpsgos.2024.100412 PMC11867118

[67] Boly, M. et al. Connectivity changes underlying spectral EEG changes during propofol-induced loss of consciousness. *J. Neurosci.* **32**, 7082–7090 (2012). 22593076 10.1523/JNEUROSCI.3769-11.2012 PMC3366913

[68] Moran, R. J., Symmonds, M., Dolan, R. J. & Friston, K. J. The brain ages optimally to model its environment: evidence from sensory learning over the adult lifespan. *PLoS Comput. Biol.* **10**, e1003422 (2014). 24465195 10.1371/journal.pcbi.1003422 PMC3900375

[69] Moran, R., Pinotsis, D. A. & Friston, K. Neural masses and fields in dynamic causal modelling. *Front. Comput. Neurosci.* **7**, 57 (2013). 23755005 10.3389/fncom.2013.00057 PMC3664834

[70] Friston, K. J., Li, B., Daunizeau, J. & Stephan, K. E. Network discovery with DCM. *NeuroImage* **56**, 1202–1221 (2011). 21182971 10.1016/j.neuroimage.2010.12.039 PMC3094760

[71] Ramesh, S. & Arachchige, A. S. P. M. Depletion of dopamine in Parkinson’s disease and relevant therapeutic options: a review of the literature. *AIMS Neurosci.* **10**, 200 (2023). 37841347 10.3934/Neuroscience.2023017 PMC10567584

[72] Jaworska, N. et al. Extra-striatal d 2/3 receptor availability in youth at risk for addiction. *Neuropsychopharmacology* **45**, 1498–1505 (2020). 32259831 10.1038/s41386-020-0662-7 PMC7360619

[73] Markello, R. D. et al. Neuromaps: structural and functional interpretation of brain maps. *Nat. Methods* **19**, 1472–1479 (2022). 36203018 10.1038/s41592-022-01625-w PMC9636018

[74] Savli, M., Bauer, A., Mitterhauser, M. & Ding, Y. Normative database of the serotonergic system in healthy subjects using multi-tracer PET. *NeuroImage* **63**, 447–459 (2012). 22789740 10.1016/j.neuroimage.2012.07.001

[75] Hansen, J. Y. et al. Mapping neurotransmitter systems to the structural and functional organization of the human neocortex. *Nat. Neurosci.* **25**, 1569–1581 (2022). 36303070 10.1038/s41593-022-01186-3 PMC9630096

[76] Naganawa, M. et al. First-in-human assessment of 11c-lsn3172176, an m1 muscarinic acetylcholine receptor pet radiotracer. *J. Nucl. Med.* **62**, 553–560 (2021). 32859711 10.2967/jnumed.120.246967 PMC8049371

[77] Kaller, S. et al. Test–retest measurements of dopamine d_1_-type receptors using simultaneous PET/MRI imaging. *Eur. J. Nucl. Med. Mol. Imaging* **44**, 1025–1032 (2017). 28197685 10.1007/s00259-017-3645-0

[78] Dukart, J. et al. JuSpace: a tool for spatial correlation analyses of magnetic resonance imaging data with nuclear imaging derived neurotransmitter maps. *Hum. Brain Mapp.* **42**, 555–566 (2021). 33079453 10.1002/hbm.25244 PMC7814756

[79] Desikan, R. S. et al. An automated labeling system for subdividing the human cerebral cortex on MRI scans into gyral based regions of interest. *NeuroImage* **31**, 968–980 (2006). 16530430 10.1016/j.neuroimage.2006.01.021

[80] Fischl, B. et al. Whole brain segmentation: automated labeling of neuroanatomical structures in the human brain. *Neuron* **33**, 341–355 (2002). 11832223 10.1016/s0896-6273(02)00569-x

[81] Friston, K., FitzGerald, T., Rigoli, F., Schwartenbeck, P. & Pezzulo, G. Active inference and learning. *Neurosci. Biobehav. Rev.* **68**, 862–879 (2016). 27375276 10.1016/j.neubiorev.2016.06.022 PMC5167251

[82] Zeidman, P. et al. A guide to group effective connectivity analysis, part 2: second level analysis with PEB. *NeuroImage* **200**, 12–25 (2019). 31226492 10.1016/j.neuroimage.2019.06.032 PMC6711451

[83] Wickham et al. Welcome to the tidyverse. *J. Open Source Softw.* **4**, 1686 (2019).

[84] Wickham, H., François, R., Henry, L., Müller, K. & Vaughan, D. dplyr: A grammar of data manipulation. R package version 1.1.4 *Github* https://github.com/tidyverse/dplyr (2023).

[85] Kuhn, M. Building predictive models in R using the caret package. *J. Stat. Softw.* **28**, 1–26 (2008).27774042

[86] Bates, D., Mächler, M., Bolker, B. & Walker, S. Fitting linear mixed-effects models using lme4. *J. Stat. Softw.* **67**, 1–48 (2015).

[87] Kuznetsova, A., Brockhoff, P. B. & Christensen, R. H. B. lmerTest package: tests in linear mixed effects models. *J. Stat. Softw.* **82**, 1–26 (2017).

