# Supplementary material for: Deviations in effective connectivity explain different hallucination subtypes in Parkinson’s disease psychosis
Source: Nat Ment Health. 2026 Jun 10;4(6):994–1009. doi: 10.1038/s44220-026-00669-7 (PMC13259922; doi:10.1038/s44220-026-00669-7)
Supplement: Supplementary file 1 — Supplementary Information Supplementary Figs. 1–15 and Tables 1–7. [file 44220_2026_669_MOESM1_ESM.pdf]

nature mental health

Supplementary information https://doi.org/10.1038/s44220-026-00669-7

Deviations in effective connectivity explain different hallucination subtypes in Parkinson's disease psychosis

In the format provided by the authors and unedited

Deviations in effective connectivity explain different hallucination subtypes in Parkinson's disease psychosis

. . .

Supplementary Information

S1. Study design details, hallucinations questionnaires and patients' clinical information and screening procedures …………………………………………………………………………………………………….. 2

S2. EEG task and preprocessing details …………………………………………………………………......... 7

S3. Analysis in sensor space ………………………………………………………………………………………. 8

S4. Additional details on source reconstruction ………………………………………………………............ 9

S5. Individual DCMs specification details ……………………………………………………………................ 12

S6. PEB analysis specification and recursive PEB details ……………………………………………………......... 15

S7. Individual regression analyses ……………………………………………………................................ 20

S8. Details on multiple regression LOOCV models ……………………………………….................................. 21

S9. Additional details on neural sources analyses ……………………………………….................................. 21

S10. Simulations details …………………………………………………………….. 23

S11. Dorsal model analyses and details ……………………………………………………………....................... 29

S12. EEG source reconstructed signal and PET binding atlases analysis …………………………………… 31

Supplementary References ………………………………………………………………………………………. 35

Supplementary Information 1. Study design details, hallucinations questionnaires and patients' clinical information and screening procedures. The study was pre-registered (Open Science Framework registration: https://osf.io/q9x7v). At the time of the pre-registration, we envisioned only ERP analyses of vMMN and DCM was not included in the original analysis plan. All the procedures here described are detailed in Vignando et al., 2024. Participants were recruited through the Parkinson's centre of excellence at King's College Hospital, Imperial College Charing Cross Hospital and Cambridge University Hospitals NHS Trust. We also recruited from a previous PD-VH study carried out at KCL (Renouf et al., 2018). We enrolled 19 PD-noVH and 21 PD-VH. Two participants were excluded due to noise affecting the quality of the data. The final sample comprises 18 PD-noVH and 20 PD-VH. For the EEG study, power analyses were conducted using G*Power to estimate the minimum sample size required to detect between-group differences in MMN amplitude. As no prior data were available for visual MMN in Parkinson's disease, we based the expected effect size (Cohen's d = 0.75) on studies of vMMN alterations in psychiatric disorders. Assuming $\alpha = 0.05$ and 80 % power, the required total sample size was a total $N = 54$ for a one-tailed test (noncentrality parameter = 2.57, df = 52, critical $t = 1.68$) and a total of $N = 60$ for a two-tailed test (noncentrality parameter = 2.89, df = 58, critical $t = 2.00$). We opted to report both tests, as no prior literature exists for vMMN in PD, though the main analyses followed the directional hypothesis of reduced vMMN in PD-VH compared to PD-noVH.

Screening. Participants had to meet internationally accepted UK Brain Bank criteria for idiopathic Parkinson's disease. Eligible participants were right-handed men or women aged 40 years or older, with a Montreal Cognitive Assessment (MoCA) score ≥ 22, and judged to be in good general health based on medical history, clinical examination, ECG, and routine laboratory tests. We pre-screened participants by phone to check for main inclusion and exclusion criteria; eligible participants underwent a study day at the clinical research facility (CRF) of King's College Hospital. After taking consent, first we collected participants' medical history, information medication, cognitive testing and psychiatric questionnaires. The study physician conducted the physical and non-motor symptoms examinations. Finally, participants completed the EEG procedure. 13 participants had a phone screening during the pandemic rather than the standard in-person screening; in these cases we administered the blind MoCA and converted the score to the full MoCA (Melikyan et al., 2021) in order to confirm eligibility (MoCA >22). One of the participants did not undergo the SCOPA-motor scale, which was the motor symptom scale chosen for this study; however, for this patient we had a recent full UPDRS (< 2 weeks), and we converted the score as recommended (Verbaan et al., 2011).

	Group	N	Mean	SD	SE
Age	PD	18	65.96	10.24	2.41
	PDP	20	68.75	8.52	1.91
PD duration	PD	18	4.49	2.89	0.68
	PDP	19	6.24	3.82	0.88
CISI-motor	PD	17	2.59	0.87	0.21
	PDP	20	2.40	0.82	0.18

SCOPA-motor	PD	17	19.35	7.74	1.88
	PDP	20	20.02	8.84	1.98
MoCA	PD	18	27.39	2.12	0.50
	PDP	20	27.15	2.76	0.62
LEDD	PD	18	302.28	226.36	53.35
	PDP	20	475.63	320.27	71.62

Supplementary Table 1. *We report participants' demographics and relevant clinical information; disease duration is reported in years, we also report CISI-PD (Clinical Impression of Severity Index for PD) for motor severity, SCOPA-motor* total score, Montreal Cognitive assessment (MoCA) scores (participants were not eligible for the study if they presented a score lower to 22) LEDD (levodopa equivalent dose*), CISI-PD was completed by a Parkinson's neurologist.*
**One PD-noVH participant did not undergo the SCOPA-motor and was excluded from this comparison.*
***LEDD: this information was not available for one PD-noVH participant, but we verified they were on both Sinemet and Madopar and thus not drug naive– based on this we used the group average of PD-noVH for this analysis.*

	F	df	p
Age	0.822	1,33	0.371
PD duration	2.476	1,33	0.125
CISI-motor	0.453	1,33	0.506
SCOPA-motor	0.059	1,35	0.809
MoCA	0.091	1,35	0.765
LEDD	3.768	1,34	0.061

Supplementary Table 2. *One-way ANOVA results (Welch's) comparing PD and PDP patients on the relevant clinical variables. The df column reflects the presence/absence of the participants who did not have SCOPA-motor or LEDD scores as mentioned in the description of Supplementary Table 1.*

We ran a Pearson's product moment correlational analysis between the clinical variables, finding that age correlated with motor severity (SCOPA) scores in the whole sample (r = .33, p=.044), and that LEDD correlated with disease duration (r=.38, p=.02) consistent with the literature. LEDD also correlated with the SCOPA-motor score (r=.50, p=.002) and the total non-motor symptom score (r=.43, p=.01).
We repeated the analysis in the PDP sample only to ascertain whether any of the psychiatric measures correlated with any clinical measures, finding that this was not the case (see Supplementary Figure 1 below). In the PDP sample, LEDD correlated with motor severity (r=.58, p=.01) as in the main sample, and motor severity with disease duration (r=.59, p=.01).

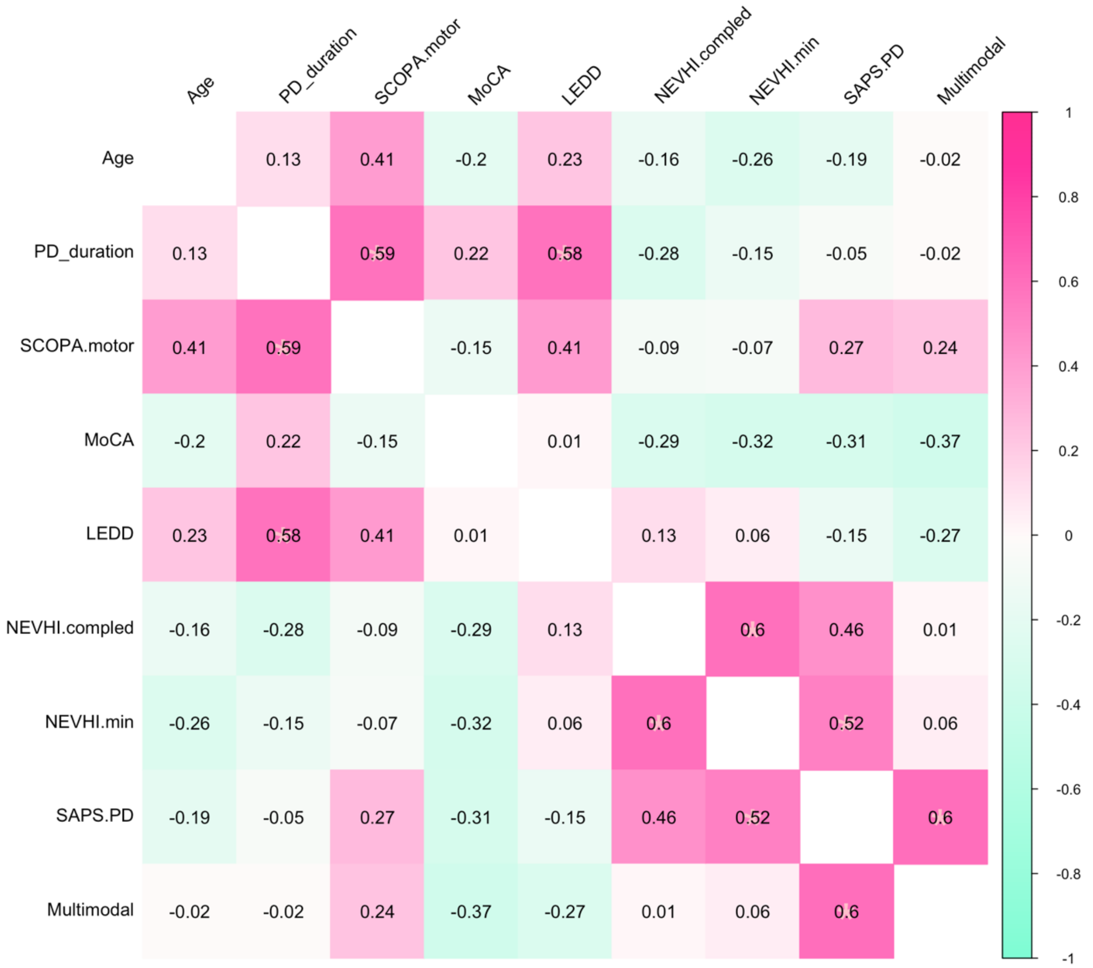

Supplementary Figure 1. *Correlation plot presenting the results described in S1; NEVHI and SAPS-PD scores were correlated with each other but not with any other clinical variable. The pink * indicates correlations that were significant with p <.05. All significant correlations were p<.01.*

Group	Age	PD-Duration	Sex	LEDD	Dopamine agonist	SSRI	Medication type
PD+VH	53	3.0	m	408.0	yes	no	rasagiline, ropirinole, Sinement, propanolol
PD+VH	55	4.0	m	500.0	no	no	Madopar, opicapone, rivastigmine
PD+VH	56	4.0	m	325.0	missing	no	no specific info
PD+VH	57	12.0	f	400.0	yes	no	pramipexole, Madopar, opicapone
PD+VH	61	5.0	m	575.0	yes	no	Sinemet, ropirinole, atorvastatin, vitamins
PD+VH	65	8.0	f	150.0	yes	yes	ropirinole, cobenacareldopa, simvastatine, diltazem, sertraline
PD+VH	67	3.0	m	445.0	no	no	opicapone, Madopar, Sinemet, rasagiline
PD+VH	68	5.0	f	100.0	no	no	co-careldopa, rivastigmine, lorazepam, clozapine
PD+VH	70	14.0	m	605.0	yes	no	amantadine, co-benaldopa, metformine, pramipexole, co-careldopa, alogliotin, amlodipine
PD+VH	70	10.0	m	1080.0	yes	yes	citalopram, co-careldopa, colecalciferol, ropirinole

PD+VH	71	4.0	f	350.0	no	no	Sinemet, amantadine, rasagiline, ramipril, cannabidiol
PD+VH	72	1.0	f	87.5	no	no	cocodamol, co-benaldopa, Madopar, tamoxifen
PD+VH	74	5.0	f	780.0	yes	no	rotigotine, Sinement, clonazepam, aziled
PD+VH	74	4.0	m	175.0	missing	no	no specific info
PD+VH	74	6.0	m	800.0	no	no	no specific info
PD+VH	75	2.5	m	0.0	no	no	drug naïve
PD+VH	75	10.0	m	635.0	yes	no	Sinemet, pramipexole, ropirinole
PD+VH	76	4.0	f	200.0	no	no	Sinemet, Madopar
PD+VH	77	13.0	m	1100.0	no	no	rsagiline, entacampone, Sinemet, co-careldopa, simvastatine, thyroxine, carcadin
PD+VH	85	7.0	m	797.0	no	no	Sinement, Madopar, stalevo
PD-noVH	74	2.0	m	625.0	no	no	Sinemet, rivaroxpane
PD-noVH	73	6.0	m	310.0	yes	no	Sinement, roprinole
PD-noVH	52	3.0	f	765.0	no	no	stalevo
PD-noVH	49	2.5	f	0.0	no	no	drug naïve, vitamins
PD-noVH	77	3.0	m	250.0	no	no	Sinemet,rasagline, entacapone, olanzapine
PD-noVH	52	1.0	f	403.0	no	no	citalopram, Siniement, rasagiline, lamotrigine
PD-noVH	73	10.0	m	150.0	no	no	Sinemet
PD-noVH	64	4.3	m	150.0	no	no	amlodipine
PD-noVH	81	9.0	m	500.0	no	no	rasagiline, Sinement, atrial fibrillation medication
PD-noVH	56	3.5	f	920.0	yes	no	co-careldopa, amantadine, ropirinol, vitamins
PD-noVH	77	3.5	f	100.0	no	no	rampiril, atorvastatin, rasagiline
PD-noVH	72	4.3	m	500.0	no	no	co-careldopa, rasagiline, atorva, lasnopraz, naproxen
PD-noVH	65	8.0	m	0.0	no	no	cocodamol
PD-noVH	49	2.0	m	560.0	yes	no	Sinemet, ropirinole, ramipril
PD-noVH	75	4.0	m	400.0	no	no	atorva, ramipril, co-careldopa, solifenacin
PD-noVH	61	2.0	m	281.0	no	no	co-careldopa
PD-noVH	66	4.2	f	250.0	no	no	Sinemet, Madopar
PD-noVH	67	10.0	f	175.0	yes	no	Sinemet, rasagiline, rotigotine, vitamins, propanolol, naproxen

***Supplementary Table 3**. Individual medication information for each participant enrolled in the study. LEDD is provided in mg.*

We conducted a chi-squared test to ensure that PD-VH and PD-noVH did not differ in terms of dopamine agonist treatment, finding that the two groups did not differ statistically (p =.2, X^2 = 2; missing information

for 2 participants). Two of our PD-VH participants were on rivastigmine. While rivastigmine has been reported to have potential benefits for psychotic and non-motor symptoms in some PD cohorts (Reading et al., 2001; Sobow, 2007), other controlled studies show limited or no effect (van Mierlo et al., 2021). Its prescription in our cohort was determined by their consultant on a case-by-case basis (NICE, 2017).

To assess whether participants had visual hallucinations we used the Neuropsychiatric Inventory (Cummings et al., 1994) at the screening visit. However, to assess the presence and the extent of hallucinations we used the SAPS-PD and the NEVHI. The Scale for the Assessment of Positive Symptoms-PD (SAPS-PD) was first adapted for PD from schizophrenia (Voss et al., 2013) and is composed of 9 questions on the symptoms that are the most frequently reported in PDP, including: auditory hallucinations, voices conversing, visual hallucinations, somatic/tactile hallucinations, global rating of hallucinations. The SAPS-PD also investigates the presence of delusions. Only one patient presented delusions in our sample, and they retained insight about it. The maximum score for each item is five, for a maximum total score of 25. Participants with >10 had also auditory or somatic hallucinations. Thus, we also computed a multimodal hallucinations score by summing together all the items that were not related to visual hallucinations and delusions, in order to explore also this aspect.

We also used an adaptation of the NEVHI to assess the phenomenology of visual hallucinations (D'Antonio et al., 2022). The NEVHI is composed of 6 main items assessing different types of VH: complex hallucinations, pareidolia (a specific type of visual illusion), presence, passage, simple hallucinations and any other visual experience of note that the researcher can classify under the relevant section. For each item, there are questions regarding the frequency and duration of the experience, questions on distress, insight and delusions specific to each type of VH. Participants' scores at the different VH types are also reported in Vignando et al., 2024 in detail.

Since this adapted version of the NEVHI has no scoring rules, we decided to compute temporal severity by multiplying duration and the frequency of the VH. In order to make the results as comparable as possible with those from other studies using the same scale, we used an ordinal scale following what D'Antonio et al., 2022 have done. We multiply the number of minutes spent hallucinating by the number of visual experiences in a month, thus obtaining temporal severity as a continuous measure. The full procedure used to create this score is reported in detail in Vignando et al., 2024, together with the % of patients experiencing the different VH subtypes. Here we report the N of hallucinations subtype per individual, together with their total SAPS-PD scores (inclusive of visual, auditory and somatosensory hallucinations and delusions, if present), and the SAPS-PD VH subscale separately

	Presence	Passage	Illusions	Multimodal	CVH	General MH	SAPS-PD tot	SAPS-PD Vis
PD+VH	3	0	5	0	1	8	10	5
PD+VH	3	0	2	0	3	5	5	5
PD+VH	5	0	4	0	3	9	5	5
PD+VH	4	0	0	0	0	4	4	2
PD+VH	0	1	0	9	0	1	12	3
PD+VH	1	6	1	7	5	8	16	5
PD+VH	8	0	0	3	2	8	15	5
PD+VH	1	0	0	3	1	1	8	3
PD+VH	0	0	0	2	5	0	14	5

PD+VH	0	5	0	0	5	5	1	1
PD+VH	4	0	4	0	0	8	5	3
PD+VH	0	0	0	8	1	0	13	2
PD+VH	4	2	3	0	4	9	14	4
PD+VH	4	0	4	6	3	8	13	3
PD+VH	4	2	2	3	5	8	5	2
PD+VH	1	0	2	0	3	3	7	2
PD+VH	8	0	16	6	8	24	23	2
PD+VH	4	0	8	3	8	12	18	5
PD+VH	0	0	0	0	3	0	5	4
PD+VH	0	3	6	4	6	9	10	4
**Total N**	**14**	**6**	**12**	**11**	**17**	**17**	**20**	**20**
**% on VH group**	**70**	**30**	**60**	**55**	**85**	**85**	**100**	**100**

Supplementary Table 4. *Individual NEVHI scores for each subscale; total SAPS-PD scores and SAPS-PD scores on the visual hallucinations scale, showing each participant had a form of visual hallucinations (question 'Do you even see things that are not there?').*

We found a difference in the MNSQ as expected, with mood, attention and perception (including hallucinations) being more affected in PD-VH (discussed in Vignando et al. 2024).

Supplementary Information 2. EEG task and pre-processing details.

The task consisted of 1000 trials, with 640 standard trials, 240 frequent deviant trials and 120 rare deviant trials. Based on results of exploratory analyses conducted during our previous ERP study we decided to focus on the standard and rare deviant difference, as the frequent deviant was perceived as not significantly different from the standard. The ERP analysis for the mismatch negativity in this sample, together with the pilot study for the task, has been reported in a previous publication where we show that PD-VH have a reduced or absent vMMN if compared to PD without hallucinations (Vignando et al., 2024).
The overt task required participants to press a button when the cross at the centre of the screen became bigger and another button when the cross became smaller. We investigated task performance to make sure participants were paying attention to the screen. We carried out a one-way ANOVA finding that PD-VH and PD-noVH did not differ in the amount of misses [$F(1,34) = 2.86$, $p = .100$], supporting the notion that they were paying attention to the task. PD-VH were significantly less accurate than PD-noVH [$F(1,34) = 10.64$, $p = .003$]. PD-noVH performed the task accurately with 91.3% of trials successfully identifying the

correct answer (mean =40.17 sd = 4.63) whereas for PD-VH the % accuracy was 70% (mean = 31.06 sd 10.90).
Overall, PD-noVH and PD-VH did not differ on RTs with PD-VH (mean 4893.64, median 5480.5, SD 2362.07, min 11, max 10149) and PD-noVH (mean 4832.23, median 5393.5, SD 1933.77, min 3, max 8670) based on a Mann-Whitney U (U = 3951.5, p = 0.435, cliff's delta = -.069). False alarms were not included.

Data was pre-processed with spm12, whereas the ERP analyses reported in the Vignando et al., 2024 publication has been preprocessed with a conceptually similar pipeline but with a different toolbox (EEGlab, Delorme et al., 2004).

Supplementary Information 3. Analysis in sensor space.

Based on our previous ERP study on this dataset (Vignando et al., 2024), we had strong hypotheses regarding involvement of parieto-occipital and frontal electrodes (POZ, PZ, P2, F2, F4, F6) around 100–180 ms, where the vMMN is typically maximal. Nevertheless, we performed an additional sensor-space analysis to guide the choice of sources and time window for DCM. Pre-processed data were converted into scalp × time images and analysed with random field theory (RFT) correction to identify spatiotemporal clusters of ERP differences between conditions. This approach, implemented in SPM, takes into account spatial and temporal correlations and is less common than mean-amplitude averaging but reduces false positives when defining data features for source analysis.

For the DCM analyses, and thus source reconstruction, we focussed on the 60–400 ms interval. This broader window was chosen for three reasons: (i) to encompass both early vMMN responses (~100–200 ms) and later activity such as the P300 (~300–400 ms), which are thought to index prediction updating; (ii) to provide sufficient temporal information for source reconstruction to guide dipole specification; and (iii) following expert methodological advice (Prof. Rosalyn Moran, co-developer of DCM neural mass models) that a wider interval is appropriate for capturing hierarchical network dynamics. The 60 ms lower bound reflects the earliest reliable latency at which deviant-related effects were observed in our data, consistent with published vMMN work. Importantly, the results of source reconstruction and model comparison converged with those obtained in the narrower 100–180 ms range, providing confidence that the broader interval did not obscure the canonical vMMN effect. Source priors were restricted to large cortical fields (V1, ITG, PFC) that are each sampled by multiple electrodes in a 64-channel montage. This macro-source strategy is appropriate for DCM-ERPs, which tests mechanistic network hypotheses rather than precise dipole localisation (see SI4).

Supplementary Information 4. Additional details on the source reconstruction analysis and coordinate tables. We used the SPM template, as we did not have T1-weighted MRI scans for all participants to co-register the EEG to. We used SPM's canonical BEM head model and template mesh. For EEG, where spatial precision is limited, template head models are widely adopted and provide stable forward models for DCM. Priors for dipole locations were centred on peaks from our sensor-space and source-space analyses, with broad covariance to reflect anatomical uncertainty. In fact, nodes were intentionally defined as large cortical regions (V1, ITG, PFC) rather than small gyri/opercular subdivisions.

For the source reconstruction process, the normal cortical mesh option was selected, and we specified the forward model using a boundary element method (BEM), then the inversion was completed. We obtained individual MIPs (maiximum intensity projection) of the activity in the source space and a time series of the activity for each condition. We used this data to generate NIfTI (.nii) images for the 60-400ms interval of interest to be investigated with the DCM analyses.

The images were used for second level analyses: we specified a one-samples t-test model to investigate the neural correlates of standard and rare deviant images, to further investigate the regions associated to the vMMN task and inform our decision as to the coordinates of the DCM dipoles (coordinates are reported in the Supplementary Table 2 below). For this analysis, we used all participants' scans to identify all possible sources, but we also repeated the source reconstruction for PD only as a sanity check, finding consistent results (results significant for rare (ITG region), no suprathreshold for standard which is expected given the smaller sample size of N=18). We also conducted paired t-tests in PD and PDP separately (rare-standard) to investigate if and where there are specific locations related to standard and deviant differences in each group that needed taking into account (Supplementary Figure 2). We explored possible differences between the frequent deviant and the standard with the same method finding no difference (no suprathreshold clusters p >.05), supporting what we found in our ERP study where no difference between the two was observed in any of the groups.

**Region**	**p(FWE-corr)**	**p(unc)**	**F**	**Z**	**x**	**y**	**z**
Left ITG	**0.003**	<.001	30.28	4.89	-48	-18	-32
Right ITG	**0.005**	<.001	28.17	4.73	54	-18	-34
Calcarine	**0.047**	<.001	21.44	4.17	2	-86	14
Left PFC	0.085	<.001	19.69	4	-40	44	-8

Supplementary Table 5*. Peak coordinates for significant clusters found in the source reconstruction analysis. The coordinates here identified helped us determine the dipole coordinates for the DCMs. We also identified a small cluster in the right precuneus (not shown), but after multiple comparisons correction the p value was >.1. Nevertheless, this was taken into account when building the dorsal model.*

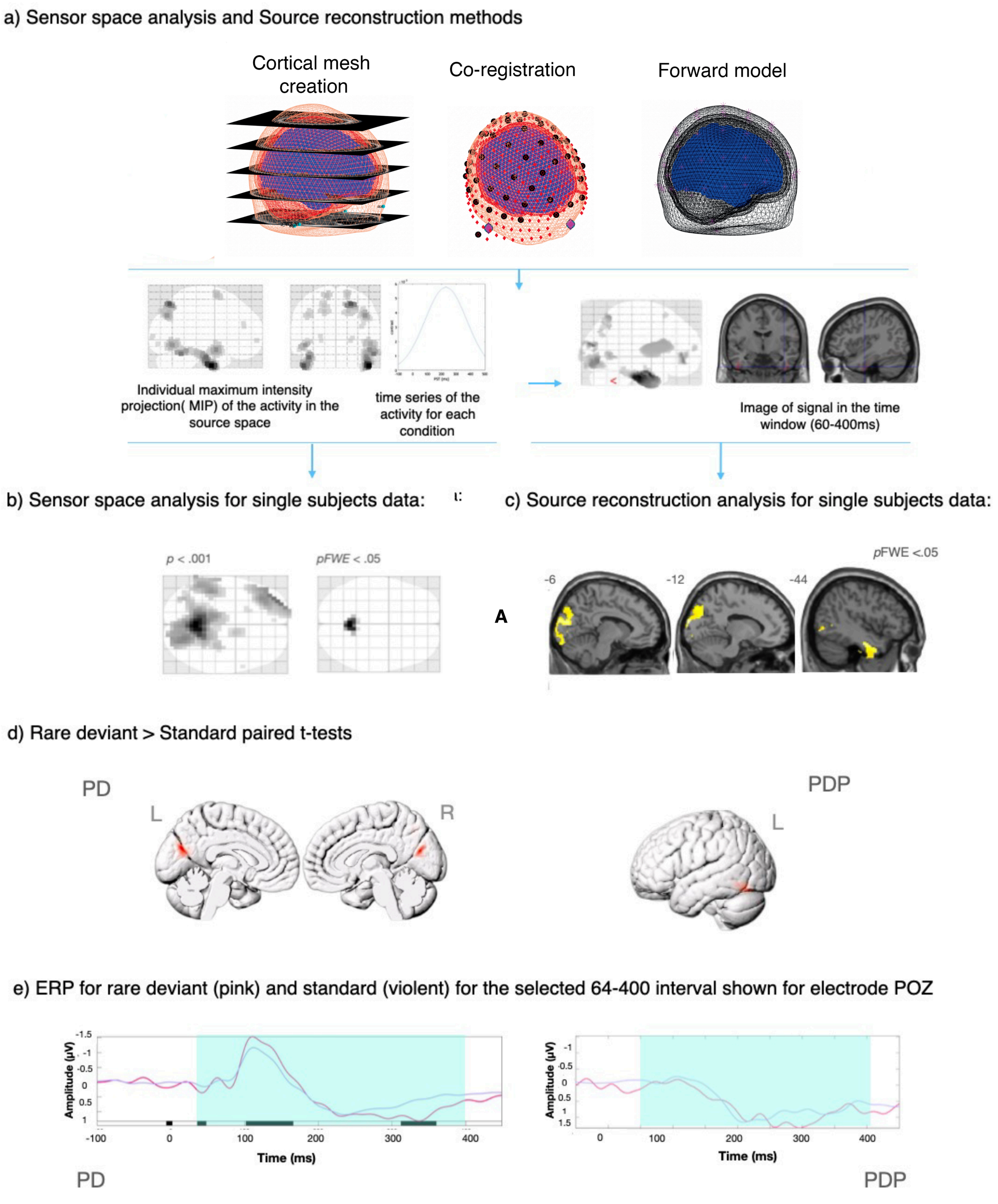

Supplementary Figure 2. *Sensor space analysis and source reconstruction analysis summary and results. a) Source reconstruction procedure. The top row shows the registration to the template and the model inversion using BEM; the * on the mesh represent the electrodes once registered on the template. The second row shows the MIP of the activity in the source space and the image generated for the signal in the time window of interest (actual data from a participant shown). b) Sensor space analysis results for the task are shown at p<.001 uncorrected and pFWE corrected <.05. c) Main results of the source*

reconstruction (entire sample) for the rare deviant trials are shown (pFWE corrected <.05). The p<.001 MIP and the MIP with the channel locations and FWE <.05.

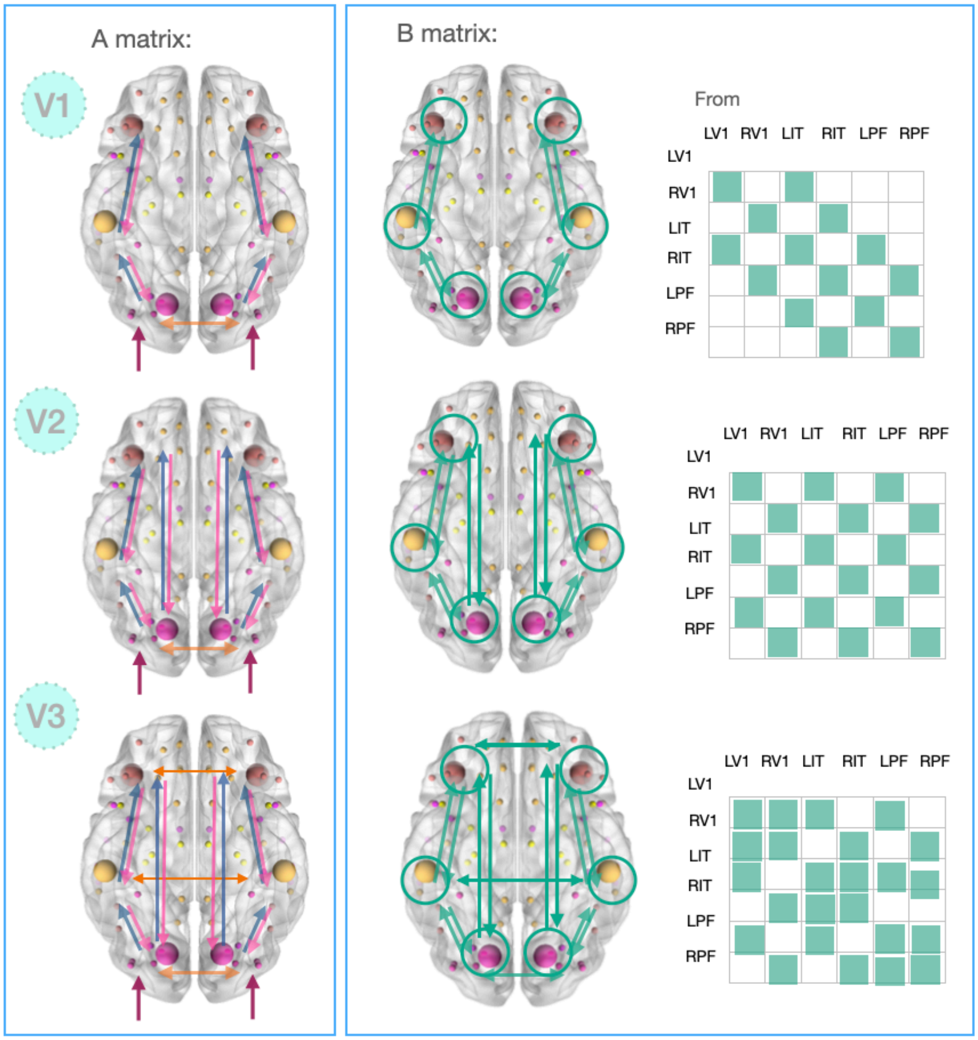

Supplementary Figure 3. Ventral models specification. *Model v1: Forward connections from V1 to ITG, V1 to PFC, ITG to PFC; backward connections from ITG to V1, PFC to ATL were specified. Lateral (intra-hemispheric) connections between regions were also specified between L and R V1 only. The differential connectivity effects matrix (B) was specified with connections from V1 to ITG, V1 to PFC, ITG to PFC, PFC to ITG, ITG to V1, and for each region as well. Model v2: Forward connections from V1 to ITG, V1 to PFC, ITG to PFC; backward connections from ITG to V, PFC to ATL, PFC to V were specified. Lateral (intra-hemispheric) connections between regions were also specified between L and R V only. The*

differential connectivity effects matrix (B) was specified with connections from V1 to ITG, V1 to PFC, ITG to PFC, PFC to ITG, ITG to V1, PFC to V, and for each region as well. model v3: forward connections from V1 to ITG, V1 to PFC, ITG to PFC; backward connections from ITG to V1, PFC to ITG, PFC-V1 were specified. Lateral (intra-hemispheric) connections between regions were also specified between left and right V1, ITG and PFC. Green circles represent connectivity on the same region, testing for self-inhibition or self-excitation during task condition.

Supplementary Information 5. Individual DCMs specification details. We used an *ERP* neuronal mass model, focussing on the 0 to 400ms interval, rare deviant vs. standard. An equivalent current dipole (ECD) was used for each source, with anterior occipital, inferior temporal and dorsolateral prefrontal regions, bilaterally (Figure 2c). For onset parameter we kept the standard 64ms onset as the propagation of the stimulus impulse through the input nodes causes a delay and large responses are usually not observed before 100ms. Modes was set at 8. Receiving areas (input) were V (L and R) specified. Details about all the models are presented in Supplementary Figure 3. Model v3 (fully connected ventral model) was used in the second-level analyses after Bayesian model selection and comprises: forward connections from V1 to ITG, V1 to PFC, ITG to PFC; backward connections from ITG to V1, PFC to ITG, PFC-V1 were specified. Lateral (intra-hemispheric) connections between regions were also specified between left and right V1, ITG and PFC. The differential connectivity effects matrix (B) was specified with connections from V1 to ITG, V1 to PFC, ITG to PFC, PFC to ITG, ITG to V1, and for each region as well. The dorsal model has the same visual and prefrontal dipoles, but with IPL replacing the ITG and no direct V1 to PFC connections. This choice was determined by results in the source reconstruction analysis (Figure 2 and Supplementary Figure 2) and by the neural correlates typically associated to this task.

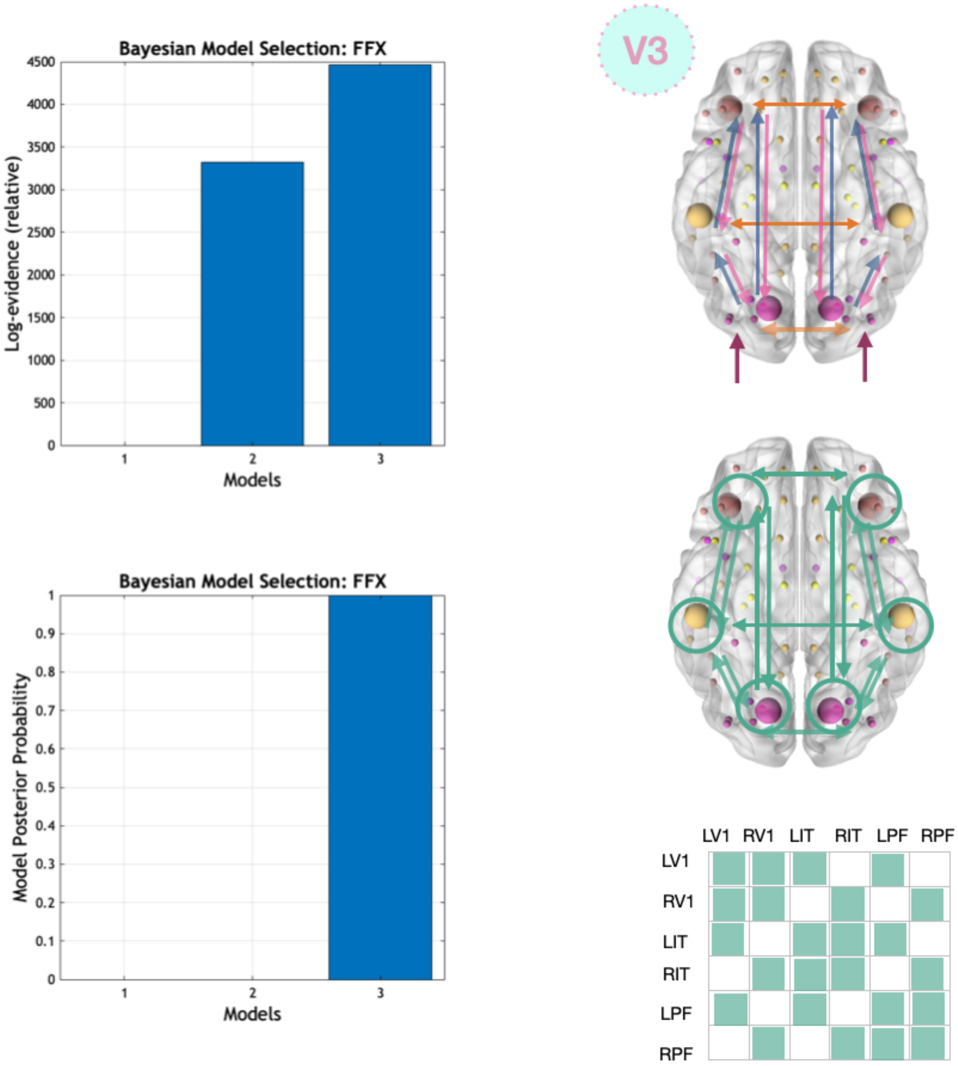

Supplementary Figure 4. BMS for ventral models. *We used Bayesian model selection to decide which of the three ventral models we specified was the winning one to the scope of focussing second-level analyses on a specific DCM. Within each pathway model, the models were compared using Bayesian model selection. BMS allows to compare different models of the same data, provided that the electromagnetic model is the same. We used a Fixed Effects (FFX) design as inference method. Model 3 was the winning model, thus we proceeded with second-level analyses with model 3, featuring also cross-hemispheric connections.*

When inspecting individual DCM fits we verified that (i) the winning model achieved good variance explained and plausible scalp fits (example in figure S5); (ii) small (±10–15 mm) perturbations of source priors did not change second-level inferences; and (iii) results were consistent with canonical MMN effects (100–180 ms) reported previously in the same dataset, which is exemplified by figure S5b, whereby we inspected scalp maps at different latencies for consistent oddball task effects.

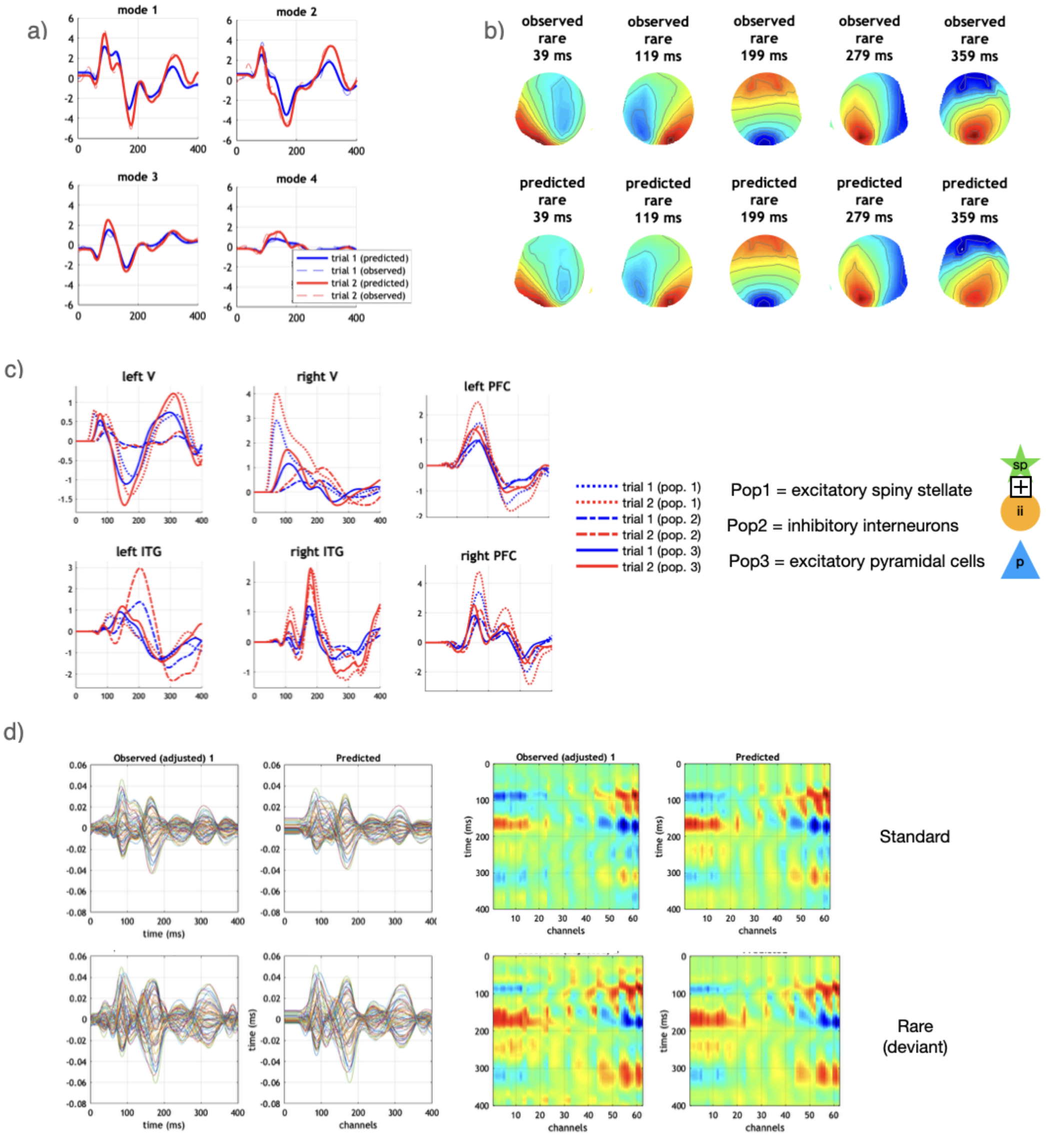

Supplementary Figure 5. Individual DCM model fits. a) *modes: example of our visual MMN model fits of the first four modes, which are the ones that captured most of the variance (out a total of eight); modes represent the predicted covariance (prior) in the sensor space. Blue = standard; red = deviant; dotted line = original data; full line = model fits. b) Scalp maps: example model fits from a single participant (c_005) in sensor space. The top row represents scalp maps the EEG data in the rare (deviant) condition; the bottom row represents the EEG data at the same latencies, generated by the model parameters. c)Sources: Dotted line (population 1) = excitatory spiny stellates; dashed lined (population 2) = inhibitory interneurons; soldi line (population 3) = excitatory pyramidal cells. Blue = standard; red = deviant. d) Response: left: representation of the data selected by the spatial modes back-projected into sensor space. Each line represents the activity of a single channel. First column = observed data; second column = model predictions. Right: The same data plotted as time as a function of channels; top= standard; bottom = rare (deviant).*

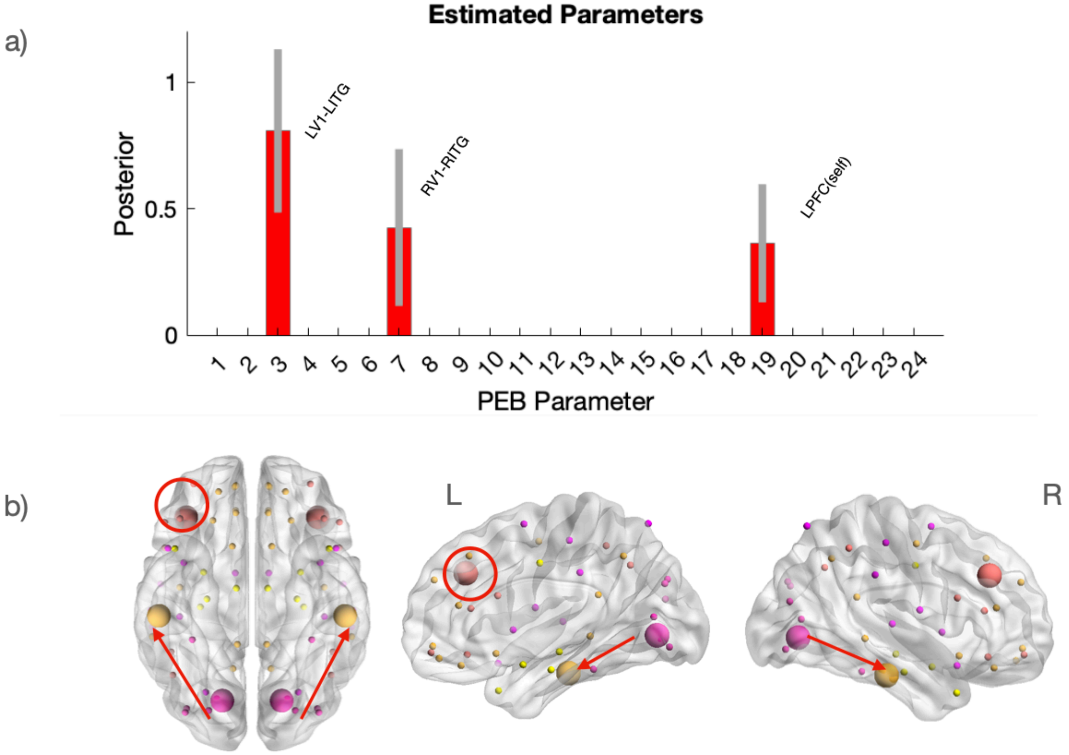

Supplementary Figure 6. *Ventral model – task connectivity in PD-noVH only. We decided to explore the effect of the task itself without including patients with hallucinations since our ERP study has shown that PD patients with VH did not have a MMN or had a very reduced one. When focussing on the difference between the standard and the deviant in PD, we found that the task was associated increased connectivity (self) in left PFC and increased V1 to ITG bilateral activity.*

Supplementary Information 6. PEB analysis specification and recursive PEB additional details.

PEB was carried out (Friston et al., 2016; Zeidman et al, 2019) at this stage. We took the modulatory parameters of the individual DCMs to the group level and used them to fit a PEB model (a type of Bayesian linear regression.) We fitted a general linear model (GLM) to the connection strengths estimated with the first level analyses and with covariates: mean, group (PD-VH (-1) or PD (1)) and age. The GLM has one regression parameter per covariate per connection. Model inversion was carried out with *spm_dcm_fit*; and *spm_dcm_bmr* and *spm_dcm_peb* were used to perform Bayesian model reduction and to generate model posteriors, respectively. Finally, we used Bayesian model averaging *spm_dcm_bma* to investigate group-level differences between patients with and without psychosis. We analysed the different B "task modulatory" matrix separately and A "baseline" matrix (forward, backward, inter-hemispheric connectivity). To understand which of the parameters contributed best to explaining variance between DCMs, we also created a model space to carry out PEB analyses recursively with specific parameters specified. By switching on and off some specific parameters and then comparing the models we can explore the effect of given connectivity features in a more direct manner. The model specified were both extrinsic

(fully connected, forward, backward, forward and self-connections, backward and self), intrinsic (coupling parameter G, timing parameter T) and a combination of extrinsic and intrinsic. Bayesian model comparison at second level was then explored and the paratamised winning 2nd level model was further examined with Bayesian model reduction (RMA). This allowed us to identify parameter changes associated with the main effect of vMMN in the two groups.

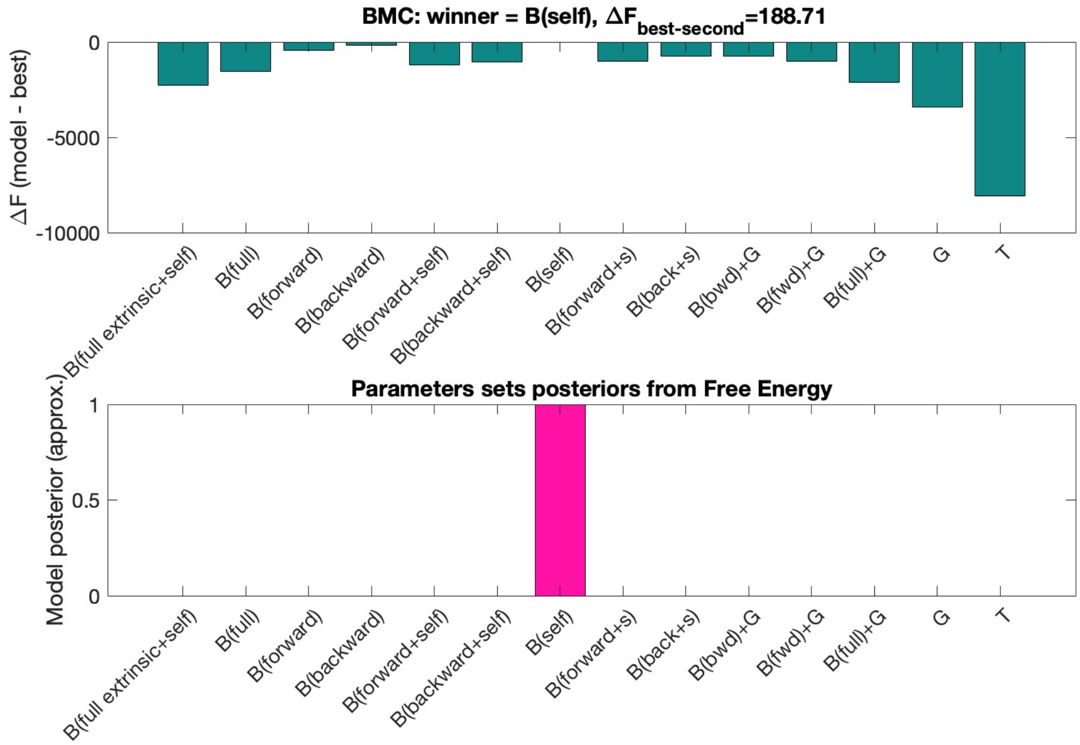

Supplementary Figure 7. Recursive PEB results. *Top row: free energy ranking and posterior probability for the different models specified (task connectivity matrix); bottom row: approximate model posterior confirming that the model with intrinsic self-connectivity parameters has the highest model evidence. Inspection of posterior probabilities revealed consistent group differences (pp > 0.99) in left V1 connectivity, as reported in the traditional PEB on the fully connected model. The next best models were very close to the self-connectivity model, and were the backward and forward connectivity models.*

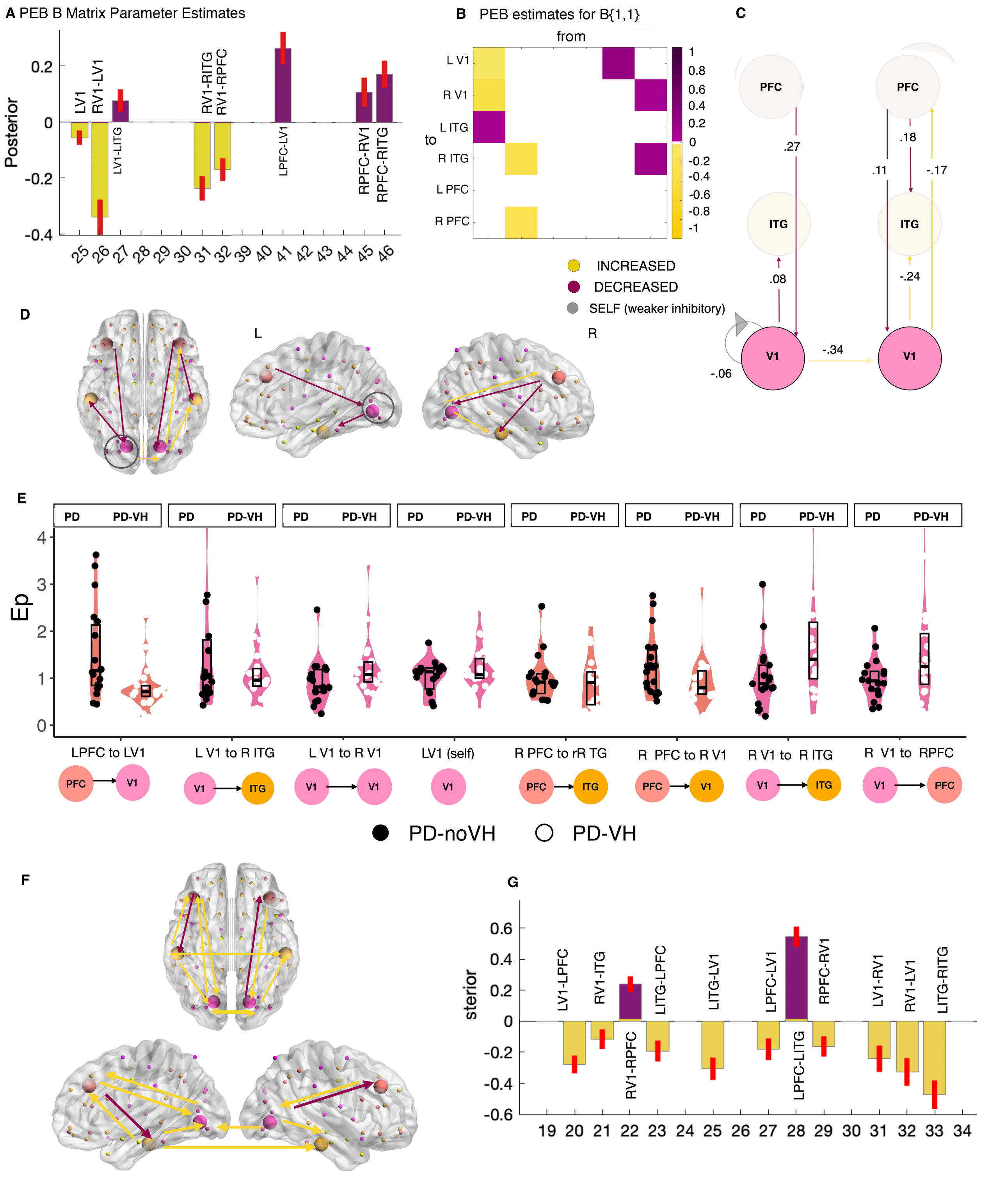

Supplementary Figure 8. PEB results on full model – with age as covariate. *Results for the VH effect are identical to those reported in the main text, except for an additional reduction in connectivity from left V1 to left ITG. When examining age effects, however, no connections showed model evidence for age effect. A-*

E. Condition specific effects (vMMN): group differences. A) bar plot of estimated connection strength (Ep) for each connection surviving the posterior probability pp>.99 (free energy) threshold. B) Connectivity matrix of results presented in (A). The matrix shows how top-down effective connectivity is reduced vs increased bottom up in VH in this task. C) Schematic of BMA results with PEB values. Yellow = increased strength in PD-VH; purple = decreased strength in PD-VH (negative values correspond to increased connectivity in PD-VH as the PD-VH group was classified with -1 in the design matrix, opposed to 1 for PD). The increased strength in the self-connection in left V1 indicates less self-inhibition. D) Graphical representation of the connections, colour coded as in a) and b). The brain and nodes have been generated with BrainNet viewer. E) box and whisker and point plots showing the distribution of Ep in PD with and without VH for each connection found differing in the model (the plots are colour coded with the colour of the node from which the connection starts; noVH patients = black dots; VH patients = white dots; the boxplots indicate the mean and the quantiles). F,G): Baseline A matrix: F, a graphical representation of the connections, colour coded as in A) and B). The brain and nodes have been generated with BrainNet viewer; G, connectivity differences in PD-VH and PD: bar plot of connection strength (Ep) for each connection surviving the pp>.99 (free energy) threshold. Yellow = increased connectivity in PD-VH; purple = decreased connectivity in PD-VH. Connectivity is overall increased in PD-VH in the connections modelled. The BMA for matrix A (forward, feedback and inter-hemispheric latent connectivity) shows that at baseline PD psychosis brain activity is positively correlated with connectivity from PFC to V1 bilaterally, from ITG to V1 bilaterally, between V1 (left to right and right to left), from left to right ITG, and from left V1 to left PFC. In addition, PD-VH activity negatively correlated with connectivity from left PFC to left ITG and from right V1 to right PFC (all with pp >.99).

Additional exploratory PEB analyses.

Primary inference was based on family-wise BMC (Free Energy). Although the G family did not outperform the winning family, some G parameters showed high posterior probability (pp > 0.99). This analysis was motivated by our simulation framework, which models excitatory–inhibitory balance via this parameter. Exploring potential group differences in intrinsic coupling therefore served as a robustness and mechanistic check. Briefly, we observed that the difference in PD with and without VH is associated with a change in cortical circuitry encoded in the intrinsic coupling parameter, finding a reduction in intrinsic connectivity from excitatory cells in right V1, left ITG and right PFC and from inhibitory cells (disinhibition) for all regions.

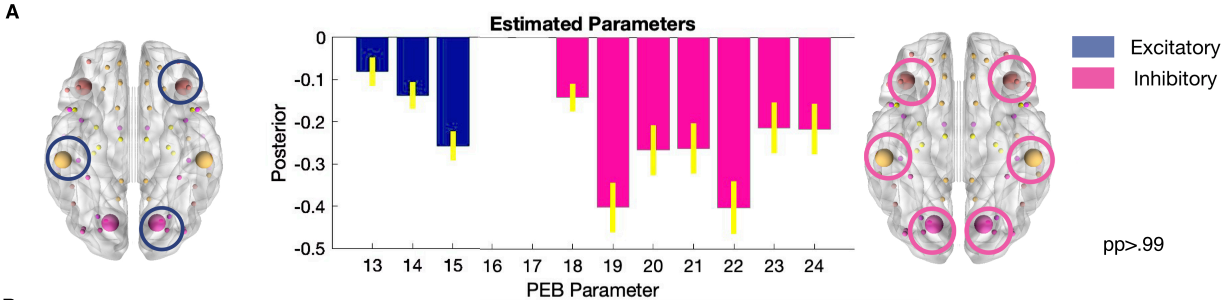

Supplementary Figure 9. Exploratory PEB on coupling parameter. The purpose of this analysis was to ensure there were reasons to pursue the simulations focussing on this parameter, reported in the main text.

Supplementary Information 7. Individual regression analyses: hallucination subtype severity and effective connectivity additional details and statistics.

All participants in the study experienced visual hallucinations. Specifically, 17 patients reported complex visual hallucinations (CVH), while the remaining three reported minor hallucinations (MH) in the form of passage (2), presence (1) and had moderate severity rating for their 'visual hallucinations' specifically at the SAPS-PD. Because some patients experienced more than one hallucination subtype, we analysed each subtype dimensionally rather than categorically: continuous severity scores for CVH, MH, or multimodality were entered as predictors in separate regression models. This approach allowed us to test whether the severity of each hallucination type correlated with specific connectivity patterns, minimising concerns about overlap between subtypes.

Matrix B (task). For the task matrix (Figure4B; SI7), decreased top-down connectivity (rPFC → r ITG) negatively correlates with the NEVHI score for complex visual hallucinations (CVH) [$F(1,18) = 4.63$, $t = -2.15$, $R^2 = .2$, CI (-4, -.04), $p = .04$, *p*FDR =.05 ; Pearson's $r = -.45$]. The severity of CVH is positively predicted by bottom-up connectivity (rV1 → rPFC) [$F(1,17)=10.27$, $t = 3.2$, $R^2 = .38$, CI(0.53, 2.57), $p = .005$, *pFDR* = .015; Pearson's $r = .61$] and rV1 → rITG [$F(1,17)=11.38$, $t = 3.37$, $R^2 = .4$, CI(0.44, 1.91), $p = .003$, *pFDR* = .015; Pearson's $r = .44$] (all analyses underwent outlier detection with Cook's distance).

MH severity correlates negatively with connectivity (decreased in PD-VH) from left PFC to left V1 [$F(1,17)=8.15$, $t = -2.85$, $R^2 = .32$, CI(-17.29, -2.57) $p=.011$, Pearson's $r = -.31$, *pFDR* <.1]; however despite outlier removal based on the visual inspection of the plot we decided to remove another participant, initially not flagged as outlier using Cook's distance but visually extreme and after this the model did not reach significance (p =.08).

To explore how our results generalise to other questionnaires, we ran regression analyses with SAPS-PD total score, finding a similar pattern for rV1 to rITG connectivity [F(1,15)=5.7, t = 2.4, $p=.03$, r2=.37; *p*FDR >.05] with higher severity scores associated to increased connection strenght. For the other connections we observed no significant results.

Matrix A (latent). When exploring this relationship for the baseline (A) matrix, we found that connectivity (increased in PD-VH) from to right V left V1 positively correlates with complex VH [F(1,16)= 6.525 , t = 2.55, p =.02, CI (0.089, 0.96), Pearson's r= .53, *p*FDR = .1].

No significant results are found for minor VH upon outlier removal.

We also computed an exploratory multimodal hallucination score using the data provided by the SAPS-PD questionnaire. Multimodality correlates with increased connectivity from left V1 to PFC [F(1,17)=10.22, t = 3.19, p =.005, *p*FDR <.05, CI (1.23, 4.11), $R^2 = .4$]. When visually inspecting hallucinations modality, we see that all patients have VH, and most of the patients who hallucinate in other sensory domains have auditory ones (only one patient, who had tactile hallucinations, also had olfactory) (Figure 4).

For all models outliers were removed using Cook's distance.

To explore minor VH subtypes (passage, presence and pareidolia), we ran the same type of models. For what concerns the MMN task (matrix B), we only found presence VH being predicted by LPFC to LV1 (decreased in PD-VH) [F(1,18)=5.04, $R^2 = .22$, p=.037, pFDR > .05] so the lower the connectivity the higher the severity scores, however upon outlier removal the model is no longer significant. For pareidolia we find a positive relationship with increased connectivity from RV1 to RITG [F(1,18)=5.57, $R^2 = .24$, p=.03, pFDR > .05], however after removing one outlier this is no longer the case. For the baseline matrix we find a positive relationship with increased cross-hemispheric connectivity from left to right V1

[F(1,18)=20.16, R^2 = .53, p=.0002, pFDR<.05] and pareidolia, but as in the previous case, upon outlier removal this is no longer true.

Supplementary Information 8. Details on ML Multiple regression models with LOOCV

Complex VH. For task connectivity (B), we found that complex VH were best predicted by connections rV1-rPFC (increased), rPFC-rITG (decreased), which were related to complex VH severity (Figure 6), plus increased rV1 to rITG connectivity [multiple R^2 = 0.58, adjusted R^2 0.49, F(3,15) = 6.86, *p*-value: 0.0039 RMSE =1.89, MAE = 1.77, R^2 =.36 ; note: one outlier was removed with Cook's distance in a multiple regression model before running the LOOCV based on the analysis presented in the previous section]. The BIC was 84.29, with a better fit than the model including all connections had BIC = 94.82. *MH* were best predicted by both lV1-rV1and left PFC-V1together [multiple R^2 = 0.40, adjusted R^2 = .32, F(2,16) = 5.28, *p*= 0.01, RMSE = 5.64, MAE = 4.3, R^2 =.11, BIC = 120.56][a].

For the baseline connectivity (A), we found that *complex VH* were best predicted by 2 (LPFC – LITG, RV1-LV1) of all the connections that were related to complex VH severity [R^2 = 0.33, adjusted R^2 = .26, F(2,17) = 4.27, *p*= 0.009, RMSE = 2.21, MAE = 1.70, BIC = 95.44].

Multimodal hallucinations had as most significant predictor the left V1-PFC connectivity [R^2 = 0.37, F(1,18) = 10.64, *p*= 0.004, RMSE = 2.55, MAE = 2.15], the connection that we found uniquely associated to this measure. However, when 4 out of the 10 connections (LV to LPFC, RV to RPFC, LITG to LV, LPFC to LIT) found differing between PD-VH and PD-noVH patients were entered as predictors of multimodality of hallucinations, the overall model was significant [R^2 = 0.6, adjusted r2=.5, F(4,14) = 4.72, *p* = 0.01, RMSE = 2.7, MAE = 2.01], BIC=98.144].

LOOCV models with multiple connections and MoCA as a covariate.

Task connectivity (B), *Complex VH.* We found that complex VH were best predicted by connections (rV1-rPFC (increased), rPFC-rITG (decreased), increased rV1 to rITG connectivity with MoCA as a covariate [R^2 = 0.6, adjusted R^2 = 0.48, F= (4,14) = 5.22, *p*-value: 0.009, RMSE =1.91, MAE = 1.65; MoCA coefficient =.42], confirming the results reported in the main text. *Minor VH* were best predicted by both lV1-rV1and left PFC-V1together with MoCA as a covariate [R^2 = 0.49, adjusted R^2 = .4, F(3,15) = 4.96, *p*= 0.014, RMSE = 6.47, MAE = 4.88; MoCA coefficient was =.1] confirming the results reported in the main text.

Minor VH, were best predicted by PFC-V1 and increased connectivity from left V1 to rV1 proved also fitting [F(3,15) =5.26, R^2 = 0.51, adjusted R^2 = .41, *p* =.01, RMSE = 5.19, MAE = 4.12]) confirming the results reported in the main text.

Multimodal score with MoCA showed similar results as those reported in the previous section [R^2 = 0.64, F(5,13) = 4.8, *p* = 0.01, RMSE = 2.7, MAE = 2.2]. Given the higher number of predictors included in the model (5 predictors, 11 individuals), we further explored the bottom-up and top-down ones separately. The bottom-up model (leftV1-leftPFC, rV1, rPFC) showed a similar pattern [R^2 = 0.47, F(3,16)

[a] A model LV1+ LV-RV+RV-RIT+ RV-RPFC +LPFC.LV+ RPFC-RV proved also fitting [R^2 = 0.572, adjusted R2 = .42, F(5,14) = 3.75, p =.023, RMSE = 5.37, MAE = 4.36] but less significant and with greater BIC = 128.26).

= 4.7, p = 0.015, RMSE = 2.8, MAE = 2.2], while the "top-down" model was not significant [R^2 = 0.15, F(3,16) = 0.9, p = 0.46, RMSE = 3.45, MAE = 2.79].

Including MoCA as a covariate modestly reduced explained variance only for complex hallucinations (MoCA $\beta \approx 0.4$), suggesting a partial overlap between cognitive decline and hallucinatory vulnerability. However, the predictive contribution of connectivity remained stable across all models, indicating that these effects primarily reflect network-specific alterations rather than general cognitive impairment. For what concerns multimodal hallucinations the overall effect remained the same, but when examining bottom-up and top-down contributions separately, global cognition seemed to be explaining most of the variance when top-down connectivity was involved.

LOOCV models with multiple connections and LEDD as a covariate.

Task connectivity (B), *Complex VH. E*e found that complex VH were best predicted by connections (rV1-rPFC (increased), rPFC-rITG (decreased), increased rV1 to rITG connectivity (not found in the individual correlations) also when adding LEDD as a covariate [R^2 = 0.58, adjusted R^2 = 0.46, F= (4,14) = .58, p-value: 0.012; LEDD coefficient =.93]. *Minor VH* were predicted by both lV1-rV1and left PFC-V1 as reported in the main text, which holds when adding LEDD as a covariate [R^2 = 0.42, adjusted R^2 = 0.3, F= (3,15) = 3.6, p-value: 0.039, RMSE =5.98, MAE = 4.66; LEDD coefficient was .47].

Baseline matrix (A). C*omplex VH* were best predicted by LPFC – LITG and RV1-LV1 connectivity also when adding LEDD as a covariate [R^2 = 0.42, adjusted R^2 = .3, F(3,15) = 3.58, p= 0.039, RMSE = 2.4, MAE = 1.7].

Also multimodal hallucinations results were confirmed with predictors LV to LPFC, RV to RPFC, LITG to LV, LPFC to LIT [R^2 = 0.57, F(5,13) = 4.8, p = 0.03, RMSE = 2.77, MAE = 2.12].

Supplementary Information 9. Additional details on neural sources analyses.

Difference in activity for rare (deviant) – standard trials in the different neural populations contributing to the DCM model, based on complex VH severity. The scope was to better understand the deficit in the PD-VH group for the difference between deviant and standard in relation to the hallucination severity. We used R packages dplyr and leave-one-out cross-validation (LOOCV) with linear regression implemented via the caret package in R (method = 'lm') to test if these model-derived estimates could significant predict VH severity. We specify VH scores as the dependent variable and activity in a specific neural population and region as an independent predictor. For each iteration, one participant was excluded from model training and used as the test case. We used LOOCV since we had a limited sample size and using the linear regression method implemented in caret allows to maximize data utilization and to minimise overfitting.
We computed the multiple regression models with total estimated activity for all 6 regions as predictors to check if results were consistent and we find the model with all regions not being significant [F(6,13) = 1.26, R^2 = .37, adjusted r^2 = .08, intercept = 0.0005, p = .3] but we observed a trend towards significance for the left ITG (t =2.006. p=.066). Thus, we proceeded to explore the neural population activity in this region, with the same model for estimated inhibitory interneurons, spiny and pyramidal activity using them as separate predictors. The first left ITG model, with the sum of all three populations was significant [F(3,16)=5.41, R^2 = .24, adjusted = 0.2, p= .03, RMSE = 2.22, MAE = 1.79]; however when using all three populations separately as predictors the model was not significant. In order to explore this result, we removed the parameters with the worst coefficients (pyramidal cells, inhibitory interneurons), finding that spiny excitatory cells estimated activity in left ITG for deviant-standard positively relates to complex VH

severity [F(1,18)= 5.863, r2 = .25, adjusted = 0.2, p=.026, RMSE = 2.20, MAE = 1.74, BIC = 94.9], meaning that the more positive the activity for deviant-standard, the more the VH, similarly to what we previously found in out ERP study (Vignando et al., 2024) the model using a standard linear regression (as this method cannot be used on LOOCV removing outliers based on Cook's distance, we find that spiny excitatory cells estimated activity positively relates to complex VH [F(1,17)=*9.088*, t = 5.43, p=.008, standa error =0.46, residual = *1.867* , intercept =2.52, R^2 = 0.35]. This result is found solely for complex VH. We tested the same model for minor VH and multimodal hallucinations score, finding no significant results. The result is in line with the finding from the recursive PEB analysis that ss for left ITG was reduced in PD-VH at task, if compared to PD.

Following the analysis of neural populations, we also explored whether there was a difference between PD and PDP for the left ITG based on the results of the regression models. When looking at the activity for each component with paired t-tests for standard vs deviant within each group, we find a difference for PD (p =.04) but not for PDP (p=.14) for inhibitory interneurons, suggesting that they might be involved in the vMMN detection, but no difference is observed for spiny activity.

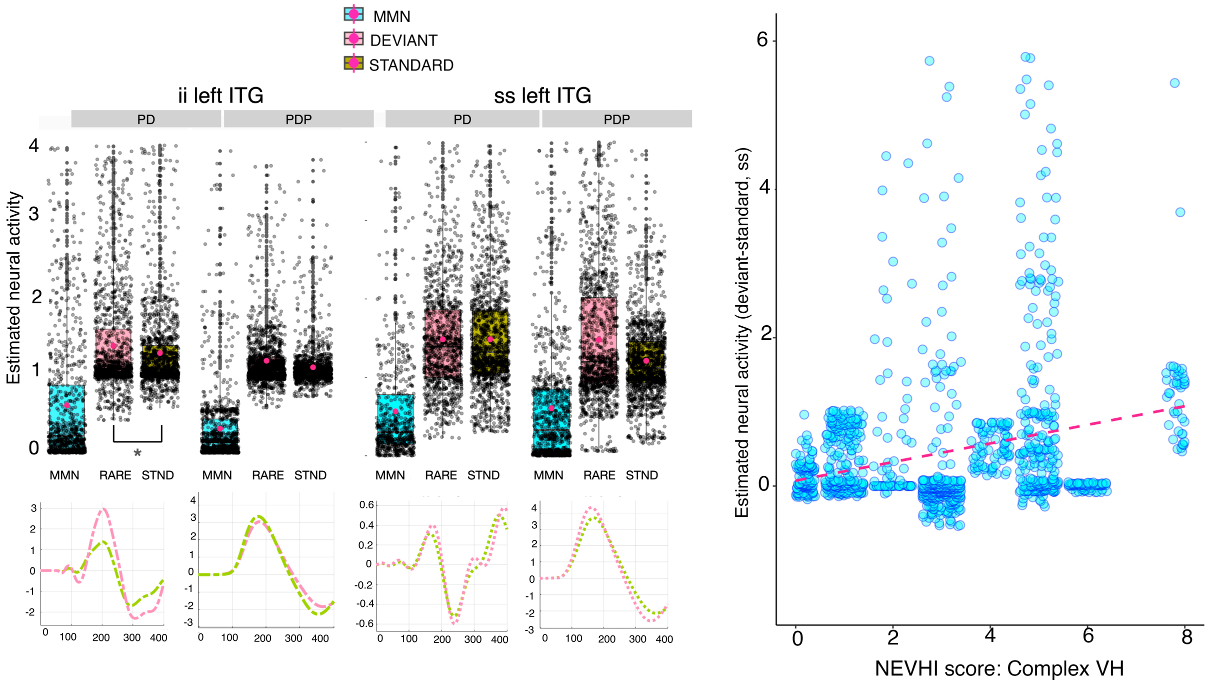

Supplementary Figure 10. Model-derived neural populations estimates and complex VH. *A) Top row: estimated neural activity for PD and PD-VH for inhibitory interneurons (left) and spiny stellate cells (right): cyan = difference (rare – standard), pink = rare, green = standard; bottom row: interneuron and spiny stellate cells activity estimated by the DCM (pink = rare, green = standard) (representation using the activity of a PD-VH and a PD patient). The data points are the values extracted from each individual DCM after model inversion for the 0-400ms for each estimated neural population and each region; the ones plotted and used for the analyses are for the 100-250ms interval, the latencies of interest for the vMMN. x axis = time ms; y axis = estimated neural activity (DCM-derived estimates). B) Scatter plot*

showing CVH severity score plotted as a function of left ITG ss cells estimated activity: based on results of a multiple regression model with total estimated activity for all 6 regions as predictors (SI9) we proceeded to explore the neural population activity in the left ITG we compared differences in activity for rare (deviant) – standard trials in the different neural populations contributing to the DCM model, based on CVH severity, finding that spiny excitatory cells estimated activity in left ITG for deviant-standard positively relates to severity, suggesting that the more positive the activity for deviant-standard, the more the VH (SI9). x axis = CVH severity score; y axis = estimated neural activity (DCM-derived estimates). We confirmed our result using a standard linear regression removing outliers based on Cook's distance; all details in full are reported in SI9, where we also describe additional exploratory analyses related differences in standard-deviant trials in PD-noVH and PD-VH in these regions where we explored neural predictors of hallucinations severity.

Supplementary Information 10. Simulations additional details.

As described in the Methods, we conducted this exploratory analysis to probe the network-level mechanisms and to understand more clearly the role of intrinsic coupling in the results pertaining to the previous sections and on the PEB analysis where specific parameter changes were found to be associated to the inhibitory to excitatory coupling. We started from the grand mean model and individual participants inversions and applied VH-related perturbations estimated from the PEB analysis. We extracted the VH contrast from the design matrix, and scaled the **G** parameter accordingly across 10 steps (0 to 2 and 0 to 0.5 ranges tested). We then used *spm_gen_erp* to generate predicted neural responses for each parameter configuration. For each simulation, we extracted excitatory and inhibitory time-series data from all regions and neural populations. We investigated both time-domain activity (using peristimulus firing-rate heatmaps) and state-space trajectories (excitatory vs. inhibitory activity over time), adapting procedures from Rosch et al.[43] and DCM perturbation work[46-47]. To quantify possible differences, we carried out paired *t*-tests ($\alpha = 0.05$) requiring at least 2 consecutive steps and computed peak *p*-values, including multiple-comparisons correction (all reported in S10). These values are descriptive of the fitted model's sensitivity to the PEB- model-derived inhibitory-gain perturbation and are not to be interpreted as independent confirmatory tests, as the purpose of these analyses is entirely illustrative and exploratory. To quantify the relative shift in inhibitory versus excitatory activity, we computed a relative disinhibition index= Delta II - 0.5(Delta PYR + Delta SS) where *Delta* represents the mean percentage change in simulated activity (VH − noVH). The 0.5 weighting averages across the two excitatory populations is applied to provide a reference for local excitation consistently with canonical neural mass models of excitatory/inhibitory balance[48-50]. We repeated the analysis both for the 100-250ms MMN latencies and for the entire trial duration to explore also later effects, compatible with the P300. In addition, the analysis was also repeated including age as a covariate. All details are reported tables in **S10**. These analyses were carried out in MATLAB 2023b.

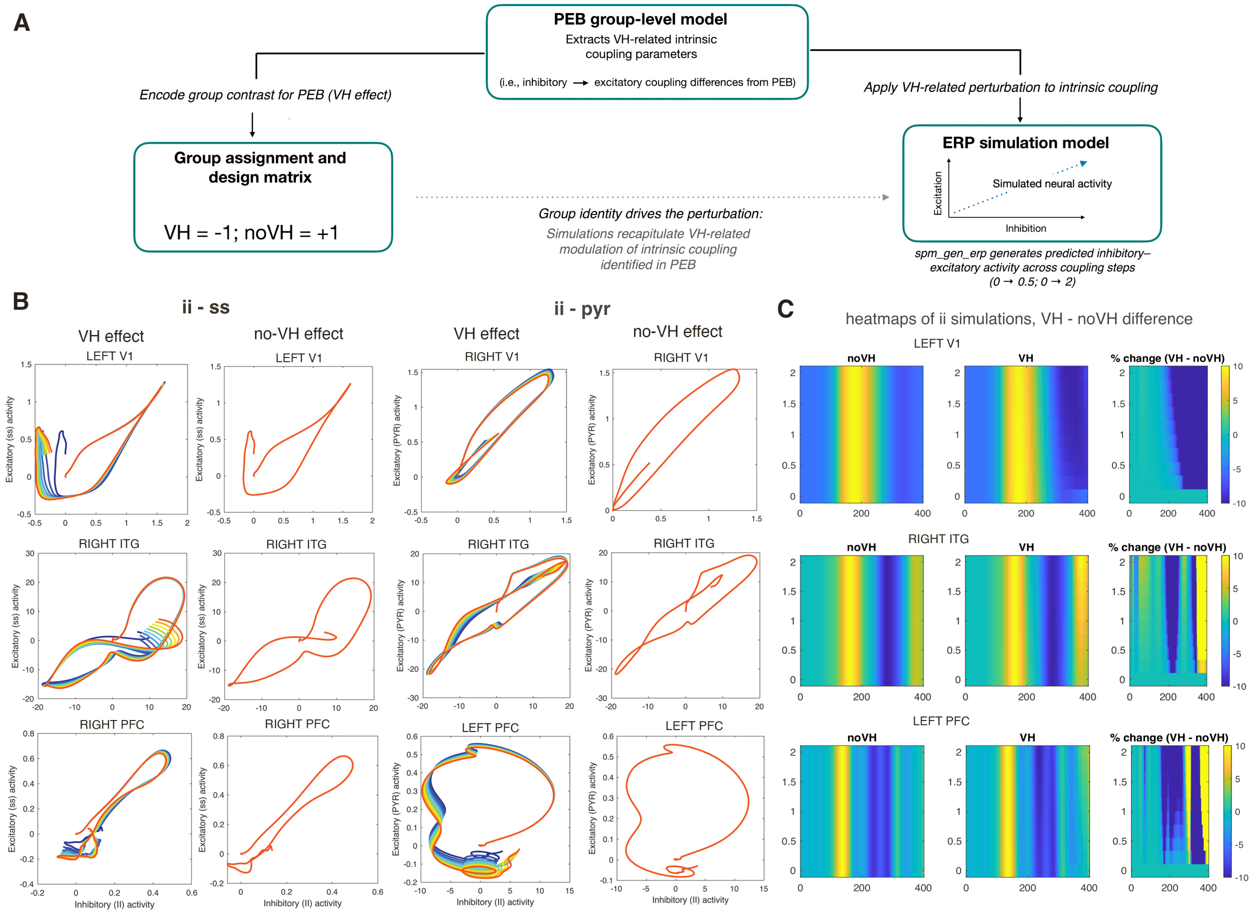

Supplementary figure 11. Intrinsic coupling modulation simulations for effect of VH results. ***A)*** *Schematic of the simulation workflow. Following the recursive PEB analysis (Methods), the VH effect was defined as the group contrast PD-VH vs. PD-noVH (second column of the PEB design matrix; PD-VH = −1, PD-noVH = +1). The corresponding group-level parameter estimate (Ep for the VH regressor) was used to scale a graded perturbation of intrinsic inhibitory gain within the ERP neural-mass model, yielding simulated changes in cortical population dynamics.* ***B)*** *State-space trajectories (excitatory vs. inhibitory activity) as G perturbation increases (blue = low perturbation, red = high perturbation). PD-VH trajectories are more irregular and multi-looped, indicating greater sensitivity and reduced stability of local circuits to small perturbations. This is exemplified by the fact that, in the no-VH condition, trajectories overlap across perturbation steps, reflecting stable network dynamics under baseline coupling. Plots are presented for the regions showing the strongest effects for each population pair (S10).* ***C)*** *Heatmaps of simulated inhibitory-population activity per region. x-axis: peri-stimulus time (ms); y-axis: inhibitory gain perturbation. Colour encodes relative activity amplitude (yellow = higher, blue = lower). Inhibitory activity in V1 and ITG shows early, sustained attenuation in PD-VH, particularly around 100–250 ms. Excitatory populations exhibit a similar but slightly delayed reduction, more visible in left V1, suggesting possible dampened cortical responsiveness. Note: Outputs are in arbitrary units (a.u.) of neural population model-derived hidden-state trajectories estimated within the DCM framework, reflecting relative estimated depolarisation changes rather than measured EEG amplitudes.*

In PD the trajectories change concurrently at higher values of the G parameter modulation, whereas PD-VH show a very complex trajectory with multiple loops. PD-VH state space plots show a higher variability (loops), suggesting that the system is more sensitive to changes.
When computing state space area in the two groups for interneurons- spiny stellate cells and interneurons and pyramidal cells the difference is statistically significant in left V1 and right ITG for ii-ss and the right ITG and the PFC bilaterally when examining ii-pyr. In this latest comparison, also V1 and the bilateral ITG shows higher sensitivity, but it does not reach formal significance.

For G 0-2: full oddball duration:
ii-pyr

Region	hemi	G at peak	peak dz eff size	sign	fir sig step	peak p value	pFDR
'ITG'	'L'	1.33	0.81	1	5	**0.000**	0.0001
'ITG'	'L+R'	1.56	0.76	1	4	**0.000**	0.0001
'ITG'	'R'	0.22	0.36	1	2	**0.032**	0.04
'PFC'	'L+R'	2.00	0.47	1	3	**0.007**	0.019
'PFC'	'L'	2.00	0.41	1	4	**0.015**	0.033
'PFC'	'R'	0.22	0.37	1	2	**0.027**	0.04
'V1'	'L+R'	2.00	0.40	1	6	**0.019**	0.034
'V1'	'R'	2.00	0.35	1	4	**0.038**	0.04
'V1'	'L'	2.00	0.33	1	NaN	0.051	0.05

ii-ss

**Region**	**hemi**	**G at peak**	**peak dz eff size**	**sign**	**fir sig step**	**peak p value**	**pFDR**
**'ITG'**	'L+R'	2.0	0.42	1	7	**0.014**	0.05
**'ITG'**	'R'	0.7	0.41	1	3	**0.015**	0.05
**'V1'**	'L'	0.2	0.40	1	2	**0.018**	0.05
**'PFC'**	'R'	2.0	0.35	1	7	**0.036**	0.08
'ITG'	'L'	2.0	0.30	1	NaN	0.077	0.13
'V1'	'L+R'	2.0	0.25	1	NaN	0.133	0.2
'V1'	'R'	2.0	0.18	1	NaN	0.267	0.34
'PFC'	'L'	2.0	-0.16	-1	NaN	0.329	0.35
'PFC'	'L+R'	2.0	-0.15	-1	NaN	0.347	0.35

For G 0-2, 100-250ms:
ii-pyr

Region	hemi	G at peak	peak dz eff size	sign	fir sig step	NS? (0 is sign)	peak p value	pFDR
'ITG'	'L'	2	0.605	1	9	0	**0.00064**	0.006
'V1'	'L'	0.22222222	0.539	1	2	0	**0.00201**	0.009
**'ITG'**	**'L+R'**	**2**	**0.461**	**1**	**9**	**0**	**0.00720**	**0.015**
**'PFC'**	**'L+R'**	**1.11111111**	**0.461**	**1**	**2**	**0**	**0.00723**	**0.015**
'PFC'	'L'	1.33333333	0.449	1	2	0	**0.00883**	0.15
**'V1'**	**'L+R'**	**0.22222222**	**0.406**	**1**	**2**	**0**	**0.01689**	**0.25**
'PFC'	'R'	2	0.211	1	NaN	1	0.20194	0.26
'ITG'	'R'	0.22222222	0.192	1	NaN	1	0.24393	0.27
'V1'	'R'	2	-0.038	-1	NaN	1	0.81829	**0.82**

ii-ss

Region	hemi	G at peak	peak dz eff size	NS? (0 is sign)	peak p value	pFDR
'V1'	'L'	2	0.244149871	1	0.140803276	ns
'V1'	'R'	2	0.143743797	1	0.381288748	ns
'V1'	'L+R'	2	0.20821049	1	0.207299071	ns
'ITG'	'L'	0.222222222	0.300198606	1	0.072231512	ns
'ITG'	'R'	2	0.301387337	1	0.071158038	ns
**'ITG'**	**'L+R'**	**0.222222222**	**0.320054771**	**1**	**0.056007388**	ns
'PFC'	'L'	0.222222222	0.169689004	1	0.30233146	ns
'PFC'	'R'	0.222222222	0.147796932	1	0.368150213	ns
'PFC'	'L+R'	0.222222222	0.172687091	1	0.293993581	ns

For G 0-2, with age as covariate in the PEB used:

ii-pyr

Region	hemi	G at peak	peak dz eff size	sign	fir sig step	NS? (0 is sign)	peak p value	pFDR
'V1'	'L'	2	0.41977672	1	6	0	**0.01372524**	<.05
'V1'	'R'	1.77777778	0.3583802	1	3	0	**0.03343094**	<.05
'V1'	'L+R'	2	0.48893457	1	4	0	**0.00463559**	<.05
'ITG'	'L'	0.66666667	0.80867676	1	3	0	**1.48E-05**	<.05
'ITG'	'R'	0.22222222	0.35930681	1	2	0	**0.03300348**	<.05
'ITG'	'L+R'	0.88888889	0.75466838	1	3	0	**4.11E-05**	<.05

'PFC'	'L'	1.33333333	0.42362365	1	3	0	**0.01294921**	<.05
'PFC'	'R'	0.22222222	0.35868972	1	2	0	**0.03328761**	<.05
'PFC'	'L+R'	1.33333333	0.4701534	1	2	0	**0.006274**	<.05

ii-ss

**Region**	**hemi**	**G at peak**	**peak dz eff size**	**sign**	**fir sig step**	**NS? (0 is sign)**	**peak p value**	pFDR
**'V1'**	'L'	0.22222222	0.39285787	1	2	0	**0.02046512**	0.05
'V1'	'R'	2	0.24892464	1	NaN	1	0.13342056	ns
'V1'	'L+R'	2	0.30448578	1	NaN	1	0.06842381	ns
**'ITG'**	'L'	1.55555556	0.45574999	1	7	0	**0.00788272**	<.05
**'ITG'**	'R'	0.44444444	0.41164233	1	2	0	**0.01550872**	<.05
**'ITG'**	'L+R'	2	0.52157856	1	4	0	**0.00270489**	<.05
'PFC'	'L'	1.11111111	-0.1601146	-1	NaN	1	0.3300472	ns
**'PFC'**	'R'	2	0.38969125	1	4	0	**0.02143011**	0.05
'PFC'	'L+R'	1.11111111	-0.1543803	-1	NaN	1	0.34744316	ns

For G 0-0.5:
ii-pyr

**Region**	**hemi**	**G at peak**	**peak dz (eff size)**	**first significant step change**	**peak p value**	**pFDR**
'V1'	'L'	0.5	0.23	NaN	0.16	>.05
'V1'	*'R'*	*0.5*	*0.33*	*NaN*	*0.05*	>.05
'V1'	'L+R'	0.5	0.29	NaN	0.08	>.05
'ITG'	'L'	0.5	0.21	NaN	0.20	>.05
'ITG'	'R'	0.5	0.37	2	**0.03**	<.05
'ITG'	*'L+R'*	*0.5*	*0.33*	*NaN*	*0.05*	>.05
'PFC'	*'L'*	*0.5*	*0.32*	*NaN*	*0.05*	>.05
'PFC'	'R'	0.1	0.38	2	**0.02**	<.05
'PFC'	'L+R'	0.5	0.37	7	**0.03**	<.05

ii-ss

Region	hemi	G at peak	peak dz (eff size)	first significant step change	peak p value	pFDR
**'V1'**	**'L'**	0.1	0.4	3	**0.02**	0.05
'V1'	'R'	0.5	0.2	NaN	0.30	0.30
'V1'	'L+R'	0.5	0.2	NaN	0.22	0.22
'ITG'	'L'	0.2	-0.2	NaN	0.26	0.26
**'ITG'**	**'R'**	0.5	0.4	8	**0.02**	0.05
'ITG'	'L+R'	0.1	0.2	NaN	0.31	0.31
'PFC'	'L'	0.1	-0.2	NaN	0.35	0.35
'PFC'	'R'	0.1	-0.1	NaN	0.44	0.44
'PFC'	'L+R'	0.1	-0.2	NaN	0.35	0.35

With age as covariate in the PEB used:
ii-pyr

Region	hemi	G at peak	peak dz eff size	sign	fir sig step	NS? (0 is sign)	peak p value
'V1'	'L'	0.5	0.26279413	1	NaN	1	0.11373157
'V1'	'R'	0.5	0.33578648	1	7	0	**0.04549386**
'V1'	'L+R'	0.5	0.32617087	1	NaN	1	**0.0516925**
'ITG'	'L'	0.5	0.64078903	1	8	0	**0.00033762**
'ITG'	'R'	0.05555556	0.36182288	1	2	0	**0.03186723**
'ITG'	'L+R'	0.5	0.6462625	1	6	0	**0.00030578**
'PFC'	'L'	0.5	0.36200011	1	7	0	**0.03178852**

ii-ss

Region	hemi	G at peak	peak dz eff size	sign	fir sig step	NS? (0 is sign)	peak p value
'V1'	'L'	0.11111111	0.40139521	1	3	0	**0.01805684**
'V1'	'R'	0.5	0.17756878	1	NaN	1	0.28076338
'V1'	'L+R'	0.5	0.2237546	1	NaN	1	0.17607793
'ITG'	'L'	0.11111111	-0.1835639	-1	NaN	1	0.26509868
'ITG'	'R'	0.05555556	0.4474948	1	5	0	**0.00897044**
'ITG'	'L+R'	0.5	0.22824208	1	NaN	1	0.16777863
'PFC'	'L'	0.5	-0.1277495	-1	NaN	1	0.43600477

Supplementary Table 6. All simulation results. Note: the statistics have a purely descriptive value as these are simulated and not real data.

RDI indexes for all regions for 100-250 ms (associated to heatmaps). **RDI = ΔII – 0.5·(ΔPYR + ΔSS)** (all as % change VH vs noVH within 100–250 ms): V1–L: RDI = 0.28848; V1–R: RDI = 0.12690; ITG–L: RDI = 1.95174; ITG–R: RDI = −9.09496; PFC–L: RDI = −6.27401; PFC–R: RDI = 2.15983

Supplementary Information 11. Dorsal pathway DCM.

We tested one dorsal model with the same visual and prefrontal dipoles, but with the IPL. This choice was determined by results in the source reconstruction analysis (Figure 2 in the main text; SI4) and by the task. The dorsal model has the same visual and prefrontal dipoles, but with IPL replacing the ITG and no direct V1 to PFC connections. This choice was determined by results in the source reconstruction analysis (Figure 2 and Supplementary Figure 2) and by the neural correlates typically associated to this task. For what concerns source reconstruction, results pointed more to the visual ventral pathway; nevertheless, we observed an inferior parietal cluster and given the fact that the MMN visual stimuli are bars with different orientation, we decided to explore the dorsal pathway as well. In addition, a study on Benton line judgement task neural correlates implicated these regions as reported in the introduction (Garcia-Diez et al., 2017).
The model setup was identical to that of the ventral model.

a) dipole locations

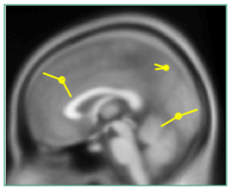

b) dipole coordinates

Region	x	y	z
L PFC	-38	33	35
R PFC	38	33	35
L IPL	-22	-70	47
R IPL	22	-70	47
L V1	10	-82	-0.5
R V1	10	-82	-0.5

c) microcircuit schematic

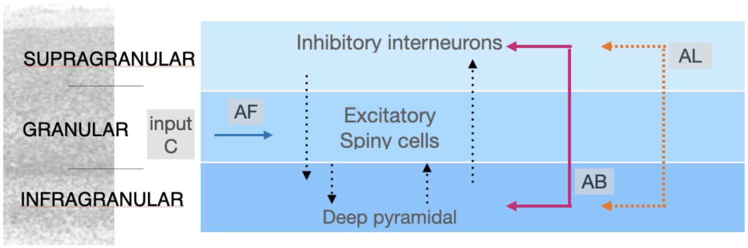

d) model connections schematic

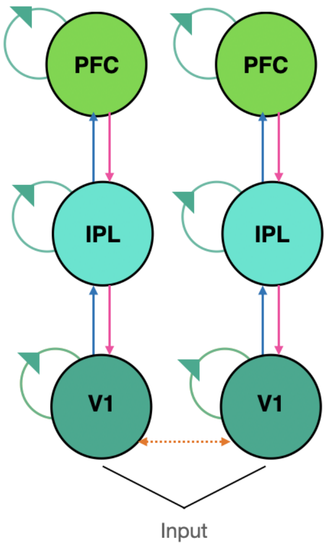

Supplementary Figure 12. ***Dorsal DCM specification.*** *a) Neural mass model and electromagnetic model specifications. b) B connectivity matrix. c) Visual representation of model v1. Left: matrix A: forward connections in blue, backward connections in fuchsia, inter-hemispheric connections in orange; input (matrix C) is depicted in orange (in L and R V1). Right: B matrix connectivity representation.*

Circles represent connectivity on the same region, testing for self-inhibition or self-excitation during task condition.

Results for the task connectivity matrix (B matrix) are reported for Free energy (with vs without) and pp > .99. The BMA results show that task effects (rare vs standard) for PDP were mostly associated to an altered connectivity between parietal and frontal nodes, with probability 100%. Examining the model parameters, psychosis was positively related to connectivity from left V1 to left IPL, and negatively correlated with connectivity from IPL to PFC, bilaterally.

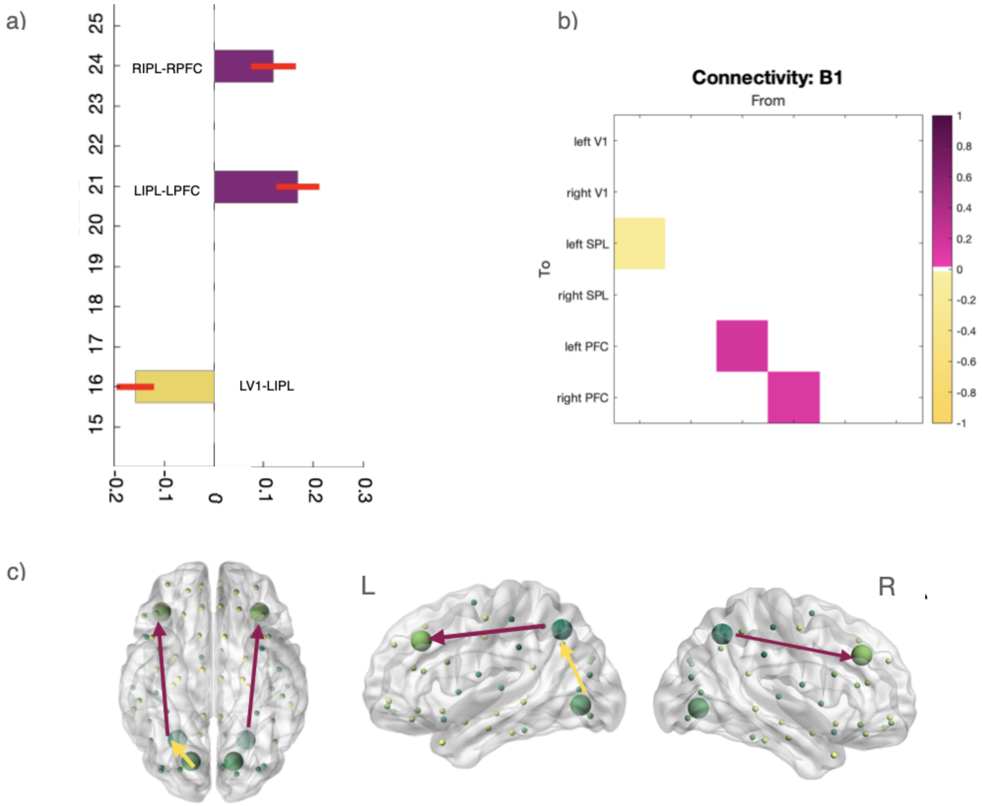

Supplementary Figure 13. ***Dorsal model PEB results.*** *a) Bar plots showing that connectivity is increased in PDP from letf V1 to IPL and decreased from IPL to PFC bilaterally. b) Matrix representation of the results shown in (a). C) Visual summary of the result. Brain and nodes are created with BrainNet viewer.*

When looking at the task effect in PD-noVH only, connectivity is only increased. Increased self-connectivity is present for V1 bilaterally, and there is an increase in connectivity from left PFC to left IPL, opposite to the decrease in connectivity from PFC to IPL found in PDP. The involvement of V1 is in line with what found in the same analysis for the ventral model strongly supporting a role of primary visual cortex connectivity in the task, consistent with the task design as well.

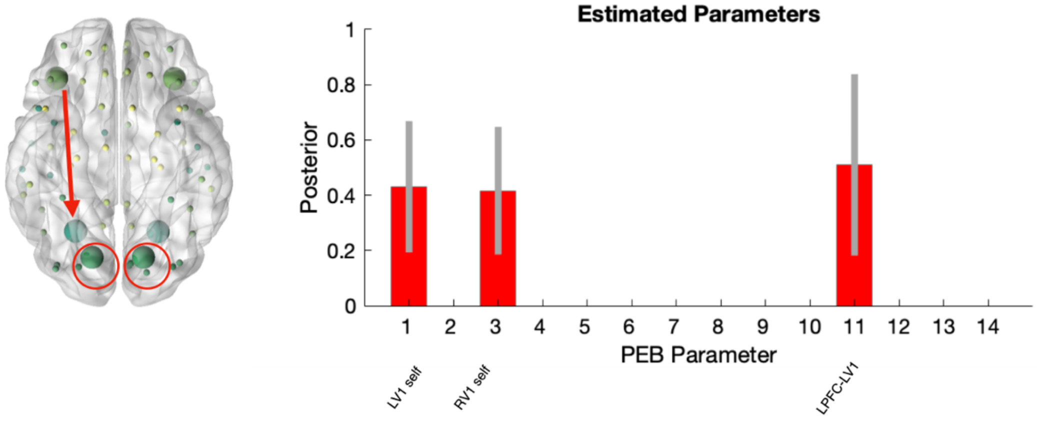

Supplementary figure 14. *Task effect (PD-noVH only) for the dorsal model. Pp > .99 (parameter prob; with free energy >.99 ns).*

Hallucination severity, subtype and multimodality analysis.

No significant correlations were found for complex VH or minor VH or multimodality for task or baseline connectivity for the dorsal model. For this reason, we did not further explore this with the leave one out logistic regressions as done for the ventral model.

***Supplementary Information 12. Exploratory receptor binding atlases and MMN source reconstructed signal analysis*: additional methods and results.**

We used source localisation data, the procedure for this has been described in the main text briefly and in detail in SI4. Then difference images were created by subtracting standard from rare activity for each participant, with each image then registered to the atlas using the *imcalc* function using the nearest neighbour interpolation method. Then we registered the Desikan-Killiany atlas (Desikan et al., 2006) to the same space as the EEG source-reconstructed signals (reslice). Image alignment was checked using checkreg in spm. We chose this atlas as the number of cortical regions resembles that of our electrodes; an atlas with higher dimensionality would introduce too much noise. The PET atlases were downloaded from https://github.com/juryxy/JuSpace/tree/JuSpace_v1.5/JuSpace_v1.5/PETatlas (Dukart et al., 2020) and neuromaps (Markello et al., 2022) https://github.com/netneurolab/neuromaps as described in the main text. A custom script was created to parcellate the source-reconstructed difference images and the PET maps of interest (5HT2-A, VAChT, M1, D2/D3, D1) in MNI template space. This allowed for the extraction of regional binding for each PET map and the corresponding reconstructed EEG signal within the regions defined by the atlas. Map and resliced atlas alignment was verified with *checkreg* and with additional code in the script comparing the image dimensions.

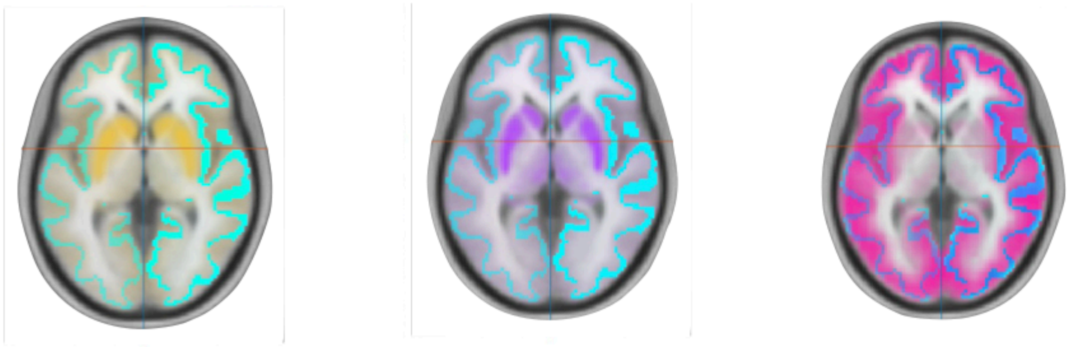

Supplementary Figure 15. ***Atlas-map alignment figures: examples from D1, VAChT, 5-HT2A.***

First, we ran individual regression models using regional receptor binding as a predictor and regional MMN signal difference (VH – noVH) as dependent variable. Before proceeding to do this, we removed all the cerebellar, WM and ventricular regions as none of these had any relationship with our EEG signal. Outliers were removed with Cook's distance and the outlier-free dataset was saved.

We found that 5-HT2_A binding significantly predicted MMN signal differences in VH – noVH patients [F(1,75]= 6.558, t =2.561 , p=.012], with a positive relationship. VAChT [F(1,69)=4.6, t= -2.145, p=.035] (results for the second vAChT map: [F(1,72)]=6.2, p=.015, t=-2.49] and D1 [F(1,75)=7.035, t=-2..65, p=.009] and D2/D3 [F(1,75)=4.622, t=-2.15, p=.035] showed a negative relationship. M1 showed no relationship (p =.3). P vales were corrected for multiple comparisons before proceeding to the next step – all values reported in the main text.

We proceeded to test whether these relationships were still true after accounting for spatial autocorrelation. Spatial autocorrelation needs to be taken into account when dealing with brain maps (or other spatial data) and it is a problem that arises from the fact that neighbouring locations tend to be more similar than locations that are more distant from one another (for a in depth discussion see Markello and Misic, 2021). Pearson's correlations were computed between each PET map and the difference in the MMN signal between participants with and without visual hallucinations (VH). To account for spatial autocorrelation, we utilized the BrainSMASH toolbox (Burt, J.B. et al., 2020). This toolbox simulates surrogate maps that match the spatial autocorrelation of the target brain map, allowing for accurate correlations between brain maps while minimizing spatial effects. Using BrainSMASH, we generated the MNI coordinates for our atlas and removed the regions we removed with the regression models, in order to run the correlational analyses on the exact same regions on which we ran the regressions. By creating centroids from the .nii atlas and running multiple permutations, we obtained a correlation coefficient that is proposed, by Burt and colleagues, to reflected the relationship between the two maps free from spatial autocorrelation. For the maps surviving the actual correlational analysis, we carried out linear mixed models using individual-level data, to test the

effect of age, MoCA score, LEDD and disease onset in this relationship. All results are reported below, together with tables with LMMs specific statistics.

Only the maps with *p*FDR <.05 were used for further analysis (corrected *p*s: D1 $p = 0.026$, 5-HT_{2A} $p = .026$, VAChT $p = .026$, D2/D3 $p = .04$; note: this was true also for the VAChT map with 4 healthy participants; M1 showed no relationship p=.3; models results are reported in SI13). Using BrainSMASH[56] we confirmed a positive correlation between 5-HT_{2A} binding and MMN signal (r=.284, p=.037) with higher connectivity in regions of higher binding. The opposite pattern was observed for D1 (r=-.29, p =.017) and VAChT (r=-2.73, p =.03). D2/D3 was no longer significant after correcting for spatial autocorrelation (p >.05).

When investigating these results using the full region-by-subject dataset using LMMs (*MMN_signal ~ _receptor * VH + age + LEDD + sex + disease onset +MoCA + (1 | Participant))*, we found for 5-HT_{2A} a main effect of 5-HT2A t =2.13,p =.034, of hallucination status (VH) t =2.52, p =.017 and a positive interaction between 5-HT_{2A} and VH t =2.06, p =.040. Among people without VH, increasing 5-HT2A by 1 SD was associated with a +0.0012 higher MMN, whereas in those with VH, 1 SD increase yielded +0.0028 higher MMN, consistent with an amplified effect of 5-HT2A in the VH group. The same model using D1 receptor availability found a significant group effect t = 2.54, p = .015, and a negative D1 *VH interaction t = –2.13, p =.033 whereby for people with VH, higher D1 values were associated with a steeper drop in MMN amplitude, overriding the otherwise higher MMN observed at average D1 levels.

For VAChT, using the map from N=18 older participants[52], we found a positive association with VAChT (t =1.75, p =.08) and a negative VAChT * VH interaction effect (t =-1.58, p =.1), though neither reached significance; VH status was significant t =2.46, p =.019. In a sensitivity analysis with the N=4 map[51], we observed the same direction of effects but with a larger effect size and formal significance (partial $r \approx –.037$ for the VAChT * VH interaction; see details in SI13). These convergent patterns suggest a negative relationship between VAChT and MMN in the VH group, though not as robust as D1.No effect of disease duration, LEDD and age was found for any of the models.

Dopamine D1

Term	Estimate	Std. Error	df	t value	Pr(>|t|)	Significance
**(Intercept)**	-0.0021	0.0020	30	-1.071	0.2929	
**d1_s**	-0.0004	0.0005	2810	-0.772	0.4399	
**VH1**	0.0045	0.0017	30	2.587	0.0148	*
**age_s**	0.0002	0.0009	30	0.223	0.8249	
**LEDD_s**	-0.0009	0.0010	30	-0.889	0.381	
**sexmale**	0.0048	0.0020	30	2.461	0.0198	*
**onset_s**	0.0007	0.0009	30	0.739	0.4655	
**MoCA_s**	0.0006	0.0009	30	0.695	0.4922	
**d1_s:VH1**	-0.0016	0.0007	2810	-2.132	0.0331	*

Acetilcholine vAChT n = 18 Alghourian et al 2017

Term	Estimate	Std. Error	df	t value	Pr(>|t|)	Significance
(Intercept)	-0.0019	0.0021	30	-0.936	0.357	
vacht_s	-0.0010	0.0006	2588	-1.745	0.0811	.
VH1	0.0045	0.0018	30	2.464	0.0197	*
age_s	0.0002	0.0009	30	0.205	0.8387	
LEDD_s	-0.0006	0.0011	30	-0.564	0.5773	
sexmale	0.0048	0.0021	30	2.301	0.0285	*
onset_s	0.0004	0.0009	30	0.442	0.6616	
MoCA_s	0.0005	0.0009	30	0.565	0.576	
vacht_s:VH1	-0.0012	0.0008	2588	-1.576	0.115	

Acetilcholine vAChT n = 5 sensitivity analysis

Term	Estimate	Std. Error	df	t value	Pr(>|t|)	Significance
(Intercept)	-0.0019	0.0020	30	-0.94	0.3549	
vacht_s	-0.0008	0.0005	2810	-1.385	0.1662	
VH1	0.0045	0.0018	30	2.52	0.0173	*
age_s	0.0001	0.0009	30	0.087	0.9312	
LEDD_s	-0.0006	0.0010	30	-0.537	0.5951	
sexmale	0.0046	0.0020	30	2.242	0.0325	*
onset_s	0.0003	0.0009	30	0.375	0.7099	
MoCA_s	0.0005	0.0009	30	0.561	0.5787	
vacht_s:VH1	-0.0015	0.0007	2810	-1.976	0.0482	*

Serotonine 5-HT2A

Term	Estimate	Std. Error	df	t value	Pr(>|t|)	Significance
(Intercept)	-0.0019	0.0020	30	-0.94	0.3549	
ht2a_s	0.0012	0.0005	2810	2.126	0.0336	*
VH	0.0045	0.0018	30	2.52	0.0173	*
age_s	0.0001	0.0009	30	0.087	0.9312	
LEDD_s	-0.0006	0.0010	30	-0.537	0.5951	
sexmale	0.0046	0.0020	30	2.242	0.0325	*
onset_s	0.0003	0.0009	30	0.375	0.7099	
MoCA_s	0.0005	0.0009	30	0.561	0.5787	
ht2a_s:VH	0.0015	0.0007	2810	2.058	0.0397	*

Supplementary Table 7. All LMMs results.

Supplementary References

Vignando, M., Ffytche, D., Mazibuko, N., Palma, G., Montagnese, M., Dave, S., ... & Mehta, M. A. (2024). Visual mismatch negativity in Parkinson's psychosis and potential for testing treatment mechanisms. *Brain Communications*, *6*(5), fcae291.

Verbaan, D., Van Rooden, S. M., Benit, C. P., van Zwet, E. W., Marinus, J., & van Hilten, J. J. (2011). SPES/SCOPA and MDS-UPDRS: formulas for converting scores of two motor scales in Parkinson’s disease. *Parkinsonism & related disorders*, *17*(8), 632-634.

Reading PJ, Luce AK, McKeith IG. Rivastigmine in the treatment of parkinsonian psychosis and cognitive impairment: preliminary findings from an open trial. Mov Disord. 2001;16(6):1171-1174

Sobow T. Parkinson's disease-related visual hallucinations unresponsive to atypical antipsychotics treated with cholinesterase inhibitors: a case series. Neurol Neurochir pol. 2007;41(3):276-279.

van Mierlo TJM, Foncke EMJ, Post B, et al. Rivastigmine for minor visual hallucinations in Parkinson's disease: a randomized controlled trial with 24 months follow-up. Brain Behav. 2021;11(8):e2257

National Institute for Health and Care Excellence (NICE).Parkinson's disease in adults. NICE Guideline NG71. 2017.

Reilly, Siobhan, et al. "The effects of rivastigmine on neuropsychiatric symptoms in the early stages of Parkinson's disease: A systematic review." *European journal of neurology* 31.2 (2024): e16142.

Cummings, J. L., Mega, M., Gray, K., Rosenberg-Thompson, S., Carusi, D. A., & Gornbein, J. (1994). The Neuropsychiatric Inventory: comprehensive assessment of psychopathology in dementia. *Neurology*, *44*(12), 2308-2308.

Voss, T., Bahr, D., Cummings, J., Mills, R., Ravina, B., & Williams, H. (2013). Performance of a shortened Scale for Assessment of Positive Symptoms for Parkinson's disease psychosis. *Parkinsonism & Related Disorders*, *19*(3), 295-299.

D’Antonio, F., Boccia, M., Di Vita, A., Suppa, A., Fabbrini, A., Canevelli, M., ... & Ffytche, D. (2022). Visual hallucinations in Lewy body disease: pathophysiological insights from phenomenology. *Journal of Neurology*, *269*(7), 3636-3652.

Vignando, M., Ffytche, D., Mazibuko, N., Palma, G., Montagnese, M., Dave, S., ... & Mehta, M. A. (2024). Visual mismatch negativity in Parkinson's psychosis and potential for testing treatment mechanisms. *Brain Communications*, fcae291.

Garrido, M. I., Kilner, J. M., Stephan, K. E., & Friston, K. J. (2009). The mismatch negativity: a review of underlying mechanisms. *Clinical neurophysiology*, *120*(3), 453-463.

Oostenveld, R., Fries, P., Maris, E., & Schoffelen, J. M. (2011). FieldTrip: open source software for advanced analysis of MEG, EEG, and invasive electrophysiological data. *Computational intelligence and neuroscience*, *2011*(1), 156869.

Delorme, A., & Makeig, S. (2004). EEGLAB: an open source toolbox for analysis of single-trial EEG dynamics including independent component analysis. *Journal of neuroscience methods*, *134*(1), 9-21.

Lopez-Calderon, J., & Luck, S. J. (2014). ERPLAB: An open-source toolbox for the analysis of event-related potentials. Frontiers in human neuroscience, 8, 21

Wickham H, François R, Henry L, Müller K, Vaughan D (2023). dplyr: A Grammar of Data Manipulation. R package version 1.1.4, https://github.com/tidyverse/dplyr, https://dplyr.tidyverse.org.

Kuhn, Max (2008). "Building Predictive Models in R Using the caret Package." Journal of Statistical Software, 28(5), 1–26. doi:10.18637/jss.v028.i05, https://www.jstatsoft.org/index.php/jss/article/view/v028i05.

Garcia-Diaz, A. I., Segura, B., Baggio, H. C., Marti, M. J., Valldeoriola, F., Compta, Y., ... & Junque, C. (2018). Structural brain correlations of visuospatial and visuoperceptual tests in Parkinson's disease. *Journal of the International Neuropsychological Society*, *24*(1), 33-44.

Desikan, R. S., Ségonne, F., Fischl, B., Quinn, B. T., Dickerson, B. C., Blacker, D., ... & Killiany, R. J. (2006). An automated labeling system for subdividing the human cerebral cortex on MRI scans into gyral based regions of interest. *Neuroimage*, *31*(3), 968-980.

Dukart J, Holiga S, Rullmann M, Lanzenberger R, Hawkins PCT, Mehta MA, Hesse S, Barthel H, Sabri O, Jech R, Eickhoff SB. JuSpace: A tool for spatial correlation analyses of magnetic resonance imaging data with nuclear imaging derived neurotransmitter maps. Hum Brain Mapp. 2021 Feb 15;42(3):555-566. doi: 10.1002/hbm.25244. Epub 2020 Oct 20.

Markello, R. D., & Misic, B. (2021). *Comparing spatial null models for brain maps. NeuroImage, 236, Article 118052*.

Markello, R. D., Hansen, J. Y., Liu, Z. Q., Bazinet, V., Shafiei, G., Suárez, L. E., ... & Misic, B. (2022). Neuromaps: structural and functional interpretation of brain maps. *Nature Methods*, *19*(11), 1472-1479.

Burt, J.B., Helmer, M., Shinn, M.W., Anticevic, A., Murray, J.D. Generative modeling of brain maps with spatial autocorrelation. Neuroimage, 220 (2020).
