# Supplementary material for: Deviations in effective connectivity explain different hallucination subtypes in Parkinson’s disease psychosis
Source: Nat Ment Health. 2026 Jun 10;4(6):994–1009. doi: 10.1038/s44220-026-00669-7 (PMC13259922; doi:10.1038/s44220-026-00669-7)
Supplement: Supplementary file 3 — Peer Review File [file 44220_2026_669_MOESM3_ESM.pdf]

nature portfolio

Peer Review File

Anomalies in effective connectivity explain different hallucination subtypes in Parkinson's disease psychosis

Corresponding Author: Dr Miriam Vignando

Parts of this Peer Review File have been redacted as indicated to maintain the confidentiality of unpublished data.

This file contains all reviewer reports in order by version, followed by all author rebuttals in order by version.

Version 0:

Reviewer comments:

Reviewer #1

(Remarks to the Author)
Vignando et al., investigate the neural alterations of visual processing in patients with Parkinson's disease and visual hallucinations (compared to patients without hallucinations). Leveraging on a visual MMN task the authors assess the temporal dynamics of EEG processing in those patients. The results show alterations in the MMN amplitude, DCM results show both reduced top-down and enhanced bottom-up connectivity in ventral visual and prefrontal regions. Furthermore, the authors identify a difference in neural alterations depending on whether the hallucinations are "minor" (left alterations) or complex (bilateral alterations). Finally, the authors also find an association with serotonergic receptor density distribution. Overall, the paper represents an impressive amount of work, is relatively novel, and is of relevance for the field.

The text might benefit from some clarity improvements:
- Are the differences in response time (during the MMN task), when pressing the button to detect the change in size of the fixation cross between groups? Similarly, what is the performance (correct, miss, etc.)?
- The data pre-processing needs more information, for instance, what is the reference used for the analyses? what is the duration of the pre-stimulus correction used? By "eye channels," you mean EOGs?
- The analyses (S13) done to define the time window of interest is not very clear and need to be more detailed. The methods used are uncommon and should be explained more and justified. And the authors should give additional explanations why not use the window found in their previous results 100-180ms, rather than performing this new analysis, and the 60-400ms windows sound particularly large.
- Is presence hallucination a visual hallucination? It's mostly described as occurring on the back of the patient or the side, mostly outside the visual field (e.g. Fénelon et al., 2011)
- Figure 1 is not very intuitive, and it's not clear whether the statistics are done for each latency separately or all together.
- The number of electrodes used seems relatively low for precise source reconstruction. How is this influencing the reliability of the results?
- Figure 4A, is visually overcomplicated for conveying a simple message, and the "no" hallucination group is additionally confusing the visualisation.
- Figure 4C, the effect seems driven by one patient. Was this regression also controlled for outliers?
- There seems that something wrong with the supplementary information - for instance I was not able to find S19, etc., making it difficult to follow and link it to the main text and validate what the authors claim.
- Do patients with MH have only MH or also complex VH? If both hallucinations are present on the same patient, how is this controlled in the results - especially for the regressions (Figure 4)

(Remarks on code availability)

(Remarks on figshare data availability)

Reviewer #2

(Remarks to the Author)
In this manuscript, the authors investigated neural dynamics underlying sensory processing deficits in PD patients with and without visual hallucinations. They showed that PD patients with visual hallucinations respond differently to the sensory environment and these differences correlate with their hallucinations types. There are a few concerns about this study:
1. The overall writing is hard to follow. There are too many unnecessary details, but lack of key information about the goal and concrete methods.
2. Many of content in statistical analysis section should move to method. Many of analyses are decided after peak of the data.
3. Analyses regarding the receptor density are poorly described. There is no description of PET data collection. (Page 8, line 230).
4. Key piece of information describing the vMMN task is lacking (only being referred to previous studies).
Minor issues:
1. ERP is not defined along with other abbreviations.
2. Figure 6 is not readable.

(Remarks on code availability)

(Remarks on figshare data availability)

Reviewer #3

(Remarks to the Author)
In hallucinations of Patients with Parkinson's disease, both "bottom-up" mechanisms arising from visual dysfunction and "top-down" processes involving attentional deficits have been implicated. This paper elucidates the relationship between these two mechanisms using a causality model and addresses types of hallucinations (minor, multimodal and complex hallucinations), representing a novel contribution of significant research value.

However, as the authors acknowledge, this study constitutes an exploratory investigation of computational models using task-based EEG data, and numerous factors related to hallucinations remain uncontrolled. This limitation warrants careful consideration in the discussion. Additionally, while the analytical methods are consistent and the statistical approaches are sound, the limited sample size necessitates modest discussion.

1.
This is a fundamental clarification, but should "IFG" in Figure 1D be considered an error for "ITG"?

2.
The image quality of Figure 6 is poor, so please replace it with a more readable figure, even if not high-resolution.

3.
In hallucinations or PwPD, two types can be considered: hallucinations and cognitive dysfunction related to cholinergic and 5-HT circuits associated with disease progression, and drug induced hallucinations associated with dopaminergic stimulation (Pagonabarraga et al. Nat Rev Neurol 2024).
This study appears to address hallucinations associated with disease progression, and the authors' logic, mainly focuses on 5-HT (and D1), is sound. However, it would be better to mention whether patients with suspected drug-induced hallucinations are included. Given that patients are taking LED 475.63±320.27mg (from SI. 1), some patients perhaps taking LED 800mg or more included. And, since participants' medication dosages are likely relevant, I have commented on this subsequently.

4.
In Figure 3D, while connectivity from lt.V1 to lt.ITG shows an increase, the right side demonstrates a decrease. How should this laterality be interpreted?

5.
From a clinical perspective, the specific medication content is more important than LED for hallucination effects. For instance, even with an LED of 900mg, hallucination risk would generally be considered different between a patient taking LD/CD 600mg + COMT-I versus a patient taking high-dose dopamine agonists. It would be desirable to specify the administered medications (presentation in supplementary materials would be enough).

It would be desirable to specify the presence/absence of cholinesterase inhibitors (presentation in supplementary materials would be acceptable).

6.
In the spirit of Open Science, facilitating comparison with other studies is important. Given that UPDRS is used as a standard in many studies and PD cohorts, if UPDRS scores are available (S11), they should be disclosed. Furthermore,

UPDRS part 1 item 2 serves as an extremely convenient assessment scale for visual hallucinations. If this item correlates with brain networks and proves to be a valid indicator, clinicians would find this highly reassuring. Conversely, if this item fails to reflect brain networks and proves ineffective, this would also constitute scientifically valuable knowledge.
This comment does not insist that UPDRS must be used (Movement Disorder Society permission procedures may also be involved). Rather, it is offered as a suggestion for potentially increasing common indicators with other research.

Minor Comment
7. Units should be added to the figures (e.g., vertical axis in Figure 1C (μV), legend on the right (ms), etc.).
8. While the figures appear to show 95% confidence intervals, these seem to go unmentioned. Explanation and numerical values should be provided.

(Remarks on code availability)

(Remarks on figshare data availability)

Version 1:

Reviewer comments:

Reviewer #1

(Remarks to the Author)
The authors responded to all my comments. I don't have additional comments

(Remarks on code availability)

(Remarks on figshare data availability)

Reviewer #2

(Remarks to the Author)
In this revision, the authors' rewriting make the manuscript much clear now.
There are still a few key concerns about how the MMN signal and PET receptor maps correlation was done and interpreted.
1. In Linear mixed model setup, only interaction term is included. This make the model hard to explain since the main and interaction effects are intertwined. Both receptor and VH effects should be listed in the model to isolate the interaction term.
2. "Individual level" was used to indicate LMM analysis. But it seems all subjects are included. It is very confusing. Shouldn't it be group level analysis for the fixed effects.
3. In figure 6B, it is clear that MMN signal and D1 binding driven by two cases at far right. I would suggest that the author run a sensitivity analysis without these two cases.
4. More fundamentally, it is not clear to me how the receptor map from a normal population should correlate with a diseased population and how these results can explain disease mechanisms. Please discuss it in the discussion section.

(Remarks on code availability)

(Remarks on figshare data availability)

Reviewer #3

(Remarks to the Author)
The authors have responded appropriately to my comments.Whilst the UPDRS was not assessed, I acknowledge that the authors have adequately examined disease severity. I am grateful for the provision of important information regarding patient demographics, including medication details.

I request minor additional revisions from the authors.

1.Antipsychotic medication
The authors have conducted an exceptionally elegant EEG analysis and obtained clear results. However, it should be noted that hallucinations in PD are characterised by their fluctuating nature. For instance, they may vary over brief periods due to medication effects, daily symptom fluctuations (wearing-off phenomenon), or even the patient's mood state during assessment (Watanabe H et al., PLoS One. 2018). Many conditions prove difficult to adjust for; however, careful patient

selection may permit control of certain factors.

One participant each was taking clozapine and olanzapine. The description "None of the participants were on quetiapine" (page 10) is therefore misleading; it should be revised to describe that "two participants were taking antipsychotic medication. At least, the authors should acknowledge that patients taking antipsychotic medications were not excluded from the study.

Cholinesterase inhibitors such as rivastigmine, which may have an comparable or greater impact on hallucinations than SSRIs, are employed as a treatment for hallucinations and can modulate brain networks (Burn D et al., Mov Disord. 2006; Pini L et al., Brain Commun. 2024). Whilst one could endlessly enumerate such minor possibilities, it is not necessary, of course, to document all of them. If SSRIs are to be described, rivastigmine warrants similar consideration.

2. Laterality
The authors' response regarding laterality was also satisfactory.
I request clarification on one additional point:
The Abstract states "Connectivity patterns differed with hallucination complexity: minor VH related to left hemisphere deficits, complex VH to altered top-down and bottom-up right-hemisphere connectivity." However, the Discussion notes "Minor hallucinations showed a similar but weaker trend predominantly involving left-hemisphere regions, though this did not survive multiple comparison correction" (page 16). In light of the Discussion, the language in the Abstract should be tempered accordingly.

(Remarks on code availability)

(Remarks on figshare data availability)

Version 2:

Reviewer comments:

Reviewer #2

(Remarks to the Author)
The authors have answered all my concerns.

(Remarks on code availability)

(Remarks on figshare data availability)
Not accessible.

Reviewer #3

(Remarks to the Author)
The authors have addressed all of my comments, and I have no further suggestions or comments.I am now fully satisfied with the revised version.

(Remarks on code availability)

(Remarks on figshare data availability)

Open Access This Peer Review File is licensed under a Creative Commons Attribution 4.0 International License, which permits use, sharing, adaptation, distribution and reproduction in any medium or format, as long as you give appropriate credit to the original author(s) and the source, provide a link to the Creative Commons license, and indicate if changes were made.
In cases where reviewers are anonymous, credit should be given to 'Anonymous Referee' and the source.
The images or other third party material in this Peer Review File are included in the article's Creative Commons license, unless indicated otherwise in a credit line to the material. If material is not included in the article's Creative Commons license and your intended use is not permitted by statutory regulation or exceeds the permitted use, you will need to obtain permission directly from the copyright holder.
To view a copy of this license, visit https://creativecommons.org/licenses/by/4.0/

Response to referees

We would like to thank all the reviewers for their careful comments and suggestions and for the insight provided, and for the time taken to review the manuscript. We have addressed all the comments raised and we provide a point-by-point response in blue below.

Miriam Vignando, on behalf of all authors

Reviewers' Comments:

Reviewer #1 (Remarks to the Author):

Vignando et al., investigate the neural alterations of visual processing in patients with Parkinson's disease and visual hallucinations (compared to patients without hallucinations). Leveraging on a visual MMN task the authors assess the temporal dynamics of EEG processing in those patients. The results show alterations in the MMN amplitude, DCM results show both reduced top-down and enhanced bottom-up connectivity in ventral visual and prefrontal regions. Furthermore, the authors identify a difference in neural alterations depending on whether the hallucinations are "minor" (left alterations) or complex (bilateral alterations). Finally, the authors also find an association with serotonergic receptor density distribution. Overall, the paper represents an impressive amount of work, is relatively novel, and is of relevance for the field.

The text might benefit from some clarity improvements:
- Are the differences in response time (during the MMN task), when pressing the button to detect the change in size of the fixation cross between groups? Similarly, what is the performance (correct, miss, etc.)?

Thanks for the feedback. First, we can confirm that for the ERPs, we have removed the 22 cross change detection trials to avoid movement artefacts. This is now specified in the methods.
For task performance, we have added to the supplementary information more details about this; task performance was analysed for the previous ERP article, but a more detailed information paragraph is now provided here. RTs were initially not analysed, because the cross change detection is only intended to keep participants engaged during the recording. We recognise the value of having this data available and have now added the following paragraph in S2.

"The overt task required participants to press a button when the cross at the centre of the screen became bigger and another button when the cross became smaller. We investigated task performance to make sure participants were paying attention to the screen. We carried out a one-way ANOVA finding that PD-VH and PD-noVH did not differ in the amount of misses [$F(1,34) = 2.86$, $p = .100$], supporting the notion that they were paying attention to the task. PD-VH were significantly less accurate than PD-noVH [$F(1,34) = 10.64$, $p = .003$]. PD-noVH performed the task accurately with 91.3% of trials successfully identifying the correct answer (mean =40.17 sd = 4.63) whereas for PD-VH the % accuracy was 70% (mean = 31.06 sd 10.90).

Overall, PD-noVH and PD-VH did not differ on RTs with PD-VH (mean 4893.64, median 5480.5, SD 2362.07, min 11, max 10149) and PD-noVH (mean 4832.23, median 5393.5, SD 1933.77, min 3, max

8670) based on a Mann-Whitney U (U = 3951.5, p = 0.435, cliff's delta = -.069). False alarms were not included."

- The data pre-processing needs more information, for instance, what is the reference used for the analyses? what is the duration of the pre-stimulus correction used? By "eye channels," you mean EOGs?

Additional details regarding pre-processing have been added in the Methods section. For your information, we copy the amended paragraph below:

We have added additional information in the methods. In particular:

1) *Task and EEG recording. We used a visual mismatch negativity task whereby the stimulus frequency variation was the orientation of peripheral bars (SI2).* ***Figure 1a*** *provides a visual summary of the vMMN task; the task design was inspired by vMMN paradigms proposed in Qian et al.*[36]*; details are provided in S2 and the task pilot and specific ERP analysis is described in detail in Vignando et al*[19]*. In brief, the explicit task required participants to press a button when the cross at the centre of the screen became bigger and another button when the cross became smaller. We investigated task performance which confirmed that participants of both were paying attention to the screen and executing the task (SI2). When performing the task, participants were seated at approximately 70cm from the CRT screen where the visual stimuli were being presented. Before every recording impedance was kept ≤ 11 kΩ.*

2) *Pre-processing: EEG data pre-processing. EEG data were pre-processed in SPM12 using the standard ERP pipeline for MMN data*[37] *(see SI2). Continuous data were converted and re-referenced using a custom average-reference montage that excluded horizontal and vertical electro-oculogram (HEO/VEG) channels. Data were high-pass filtered at 0.3 Hz (fifth-order Butterworth, two-pass), down-sampled to 500 Hz, and low-pass filtered at 30 Hz (fifth-order Butterworth, two-pass). Epochs were defined from −100 to 500 ms around stimulus onset for standard and rare deviant conditions (shift triggers = 0). Baseline correction was applied using the −100 to 0 ms pre-stimulus interval. Artefactual trials and channels were identified by visual inspection (FieldTrip) and automatically rejected using an 80 µV threshold. Remaining trials were averaged separately for standard and deviant conditions before source reconstruction (see S2).*

- The analyses (S13) done to define the time window of interest is not very clear and need to be more detailed. The methods used are uncommon and should be explained more and justified. And the authors should give additional explanations why not use the window found in their previous results 100-180ms, rather than performing this new analysis, and the 60-400ms windows sound particularly large.

We agree with the reviewer that the analysis windows need to be more clearly addressed. The main reason for this difference is the use of DCM, as DCM relies on biologically grounded models to investigate how EEG signals are generated rather than summarising and averaging evoked responses of images.

We decided to focus on a wider time window than the standard ERP analysis focussed on the MMN. Within the 100–180ms our visual MMN was maximal, but in addition we had also examined the entire duration of the task for within-group standard-deviant analyses, as we also wished to explore the P300 component. This gives us an extended time window for the response.

For the present DCM analyses, we decided to keep an extended window of 60–400 ms. The rationale was threefold: (i) to encompass both early MMN activity and later components such as the P300 (300–400ms), which are thought to reflect prediction updating beyond early error detection and which in our previous studies were observed in both groups (even if not surviving multiple comparisons correction in PD-VH); (ii) to provide sufficient data for source reconstruction to guide the specification of dipoles for the generative models; and (iii) following advice from Prof. Rosalyn Moran (co-developer of the neural mass models used in DCM), who advised us during the first steps of the individual DCMs setup and suggested that a broader interval would be more appropriate for capturing the hierarchical dynamics of interest in this task, including the earliest time at which deviant-related effects occurred.

We now state more explicitly our reason for choosing this time window in the revised text, including clarification that the 60ms lower bound reflects the earliest reliable latencies at which deviant-related effects were observed in our data, consistent with published vMMN studies. Importantly, our analyses converged on results consistent with the 100–180 ms MMN interval, increasing confidence that our broader window did not obscure the key effect.

The now amended section in the methods reads "*We focus on a wide interval (60-400ms) rather than on a narrower one (e.g. 100-180ms, 100-250ms) where MMN usually peaks, for a number of reasons. First, to avoid limiting the analysis to one level of the hierarchy only (Mancini and Nour, 2025) and to encompass both early MMN activity and later components such as the P300 (300–400 ms), which are thought to be modulated by probability as well at a higher level (involuntary attention) of the hierarchy and which in our ERP study were observed in both groups (even if not surviving multiple comparisons correction in PD-VH). Second, to provide sufficient data for source reconstruction to guide the specification of dipoles for the generative models; (iii) to leverage DCM's ability to provide an explanation of the complex neural dynamics generating the EEG signals of interest. As DCM relies on biologically grounded models to investigate how EEG signals are generated rather than summarising and averaging evoked responses of images (Kiebel et al 2008; Kiebel et al. 2006) choosing a wider interval was in the interest of capturing the earlier and later dynamics associated to our task. The 60 ms lower bound reflects previous finding that visual information is found in the primary visual cortex as early as ~60ms after stimulus onset (e.g. Luck, 2012).*"

- Is presence hallucination a visual hallucination? It's mostly described as occurring on the back of the patient or the side, mostly outside the visual field (e.g. Fénelon et al., 2011).

We agree that presence hallucinations are not strictly visual, as they are typically described as the vivid sensation of another person being nearby, often outside the visual field (e.g. Fénelon et al., 2011). However, presence hallucinations are widely considered part of the Parkinson's disease psychosis spectrum, alongside visual hallucinations, and are frequently included in clinical and research assessments of hallucinations in PD (see Bernasconi et al., 2016, *Nat Rev Neurol*). Although not purely visual, their phenomenology often involves a strong percept of another person (despite them not actually

seeing this), sometimes with a fleeting or implied visual component, which links them conceptually to the visual hallucination domain.

In our study, we therefore included presence hallucinations within the broader framework of hallucination subtypes, while acknowledging that they lie at the boundary between visual and non-visual phenomena. We now clarify this distinction in the revised manuscript, together with the additional information that 17 out of 20 participants had CVH and only 3 had MH, however they both had specified they had some form of visual hallucinations when administered the SAPS-PD. In addition, S7 we further clarify this and Figure 4 gives a more comprehensive overview of this.

- Figure 1 is not very intuitive, and it's not clear whether the statistics are done for each latency separately or all together. We have now replaced Figure 1 with a clearer version showing distribution of negative and positive amplitude at POZ for each latency separately.

- The number of electrodes used seems relatively low for precise source reconstruction. How is this influencing the reliability of the results?

We agree with the reviewer that high-density EEG would be ideal and have added this to the limitations. However, we think our analysis is reliable. First, the aim of the study was not to identify the functional architecture of the visual MMN response, which has been previously done (*Susac, Ana, et al. "Magnetic source localization of early visual mismatch response." Brain topography 27.5 (2014): 648-651,* showing their vMMN source in the calcarine, where we also found our main source), but rather to inform our choice of DCM nodes. Second, DCM for ERPs does not rely on fine-grained source localisation but rather on testing mechanistic models with a small number of macro-sources/equivalent current dipoles; these represent extended cortical patches. In this context, 64-channel montages with a template BEM head model are standard and have been used extensively in previous SPM/DCM ERP work (we have added details in Methods/supplement to support this statement, but for example Adams et al., 2022 using 64 channel EEG; Rosch et al., 2019 Biol Psychiatry using 20 channels low density EEG and perform source reconstruction for DCM and simulations as we did; both references are present in the main text).

The choice of this approach and of the use of a template was taken also after expert guidance (Prof. Rosalyn Moran) that individual MRIs are not essential here for source localisation because scalp EEG has inherently limited spatial precision and DCM's inference rests on the forward model fit rather than millimetre accuracy. Following Prof. Moran's advice, to minimise any impact of sensor density on localisation, we (i) constrained sources to large, well-separated regions (bilateral V1, ITG, PFC), each covered by multiple electrodes; (ii) we had a strong hypotheses based on previous literature about the dipoles but decided to guide priors by sensor-space and source-space analyses.

We also ran robustness checks: the winning model explained the data well (model fits shown in S5, including example of scalp topography which was examined for every participant), and results were concordant with our prior ERP findings in the canonical MMN latency range. Finally, for what concerns our exploratory PET–EEG analysis, we parcellated at atlas scale with a granularity as close as possible to the sensor density and corrected for spatial autocorrelation; we explicitly frame those results as hypothesis-generating rather than precise localisation.

To make this explicit, we have added clarifying text to Methods and S3–S5 outlining the rationale for (a) 64-channel montage with template head model, (b) choice of large cortical nodes, and (c) robustness steps.

- Figure 4A, is visually overcomplicated for conveying a simple message, and the “no” hallucination group is additionally confusing the visualisation. – We have changed the plot in 4A using an Upsit plot to allow to see overlaps across different hallucination subtypes. We hope that this is a clearer visualisation of the prevalence of hallucination subtypes in the study sample.

- Figure 4C, the effect seems driven by one patient. Was this regression also controlled for outliers?

We used Cook’s distance and removed all outliers identified as such; this individual had indeed very frequent minor hallucinations that increased their severity score. However, when using Cook’s distance this was not marked as an outlier and thus we had a principled reason to keep them in the model. Nonetheless, if we remove both the data point identified as an apparent outlier, and this specific individual the model no longer reaches significance. Because of this, the reviewer’s query and also because we had already reported that the analysis did not survive correction for multiple comparisons, we decided to remove this result and mention this in S7. This does not impact our main findings or the discussion.

Redacted

- There seems that something wrong with the supplementary information - for instance I was not able to find S19, etc., making it difficult to follow and link it to the main text and validate what the authors claim.

Apologies. this was meant to be supplementary information 9. We have now removed the I and refer to each supplementary information point with S1, S2 .. S9.

- Do patients with MH have only MH or also complex VH? If both hallucinations are present on the same patient, how is this controlled in the results - especially for the regressions (Figure 4) –

Thank you for this comment. We have clarified this point in the manuscript. In our sample, 17/20 patients experienced complex visual hallucinations (CVH)**,** while the remaining three reported minor hallucinations (MH) in the form of passage (2), presence (1) and provided a moderate severity rating for the visual hallucinations items specifically at the SAPS-PD. Most (n=17) of participants with CVH also presented MH. Thus, all participants included in the study had visual hallucinations. A table has now been added to the Supplementary Information S1 to detail the distribution of hallucination subtypes across individual participants (see S1) and we have added in S7 additional details before presenting all the statistics: "All participants in the study experienced visual hallucinations. Specifically, 17 patients reported complex visual hallucinations (CVH), while the remaining three reported minor hallucinations (MH) in the form of passage (2), presence (1) and had moderate severity rating for their 'visual hallucinations' specifically at the SAPS-PD. Because some patients experienced more than one hallucination subtype, we analysed each subtype dimensionally rather than categorically: continuous severity scores for CVH, MH, or multimodality were entered as predictors in separate regression models. This approach allowed us to test whether the severity of each hallucination type correlated with specific connectivity patterns, minimising concerns about overlap between subtypes." We are happy to move this to the Methods in the main text if necessary.

Regarding the regression analyses (Figure 4), the aim was not to compare groups defined by hallucination subtype, but to examine whether the severity of each hallucination subtype (entered as continuous scores) correlated with specific effective connectivity changes. This approach minimises concerns about overlap, as each regression tested within-subtype variability in severity rather than categorical group differences. In other words, even if patients experienced both CVH and MH, the models evaluated whether the intensity of a given symptom dimension was associated with connectivity patterns, consistent with dimensional approaches used in recent work (e.g. D'Antonio et al., 2022).

Reviewer #2 (Remarks to the Author):

In this manuscript, the authors investigated neural dynamics underlying sensory processing deficits in PD patients with and without visual hallucinations. They showed that PD patients with visual hallucinations respond differently to the sensory environment and these differences correlate with their hallucinations types. There are a few concerns about this study:

1. The overall writing is hard to follow. There are too many unnecessary details, but lack of key information about the goal and concrete methods.

Thank you for this helpful suggestion, we took the opportunity of this revision to add details in the methods section and clarify the aims of the study. As it is highlighted in the tracked version of the manuscript, we have reworked different section of the manuscript to improve readability.

2. Many of content in statistical analysis section should move to method. Many of analyses are decided after peak of the data.

We thank the reviewer for pointing this out. We have now moved details of EEG preprocessing, modelling (DCM, simulations) and exploratory receptor mapping into the Methods section, leaving the Statistical analysis section to describe only the inferential tests and correction procedures. This should improve clarity and distinguish modelling from statistical inference.

3. Analyses regarding the receptor density are poorly described. There is no description of PET data collection. (Page 8, line 230).

Thank you for raising this point. To avoid confusion, we now explicitly state that no PET data were acquired in this study. Instead, we used published normative receptor density atlases derived from independent PET datasets, as now further clarified in the revised Methods section and in the figure description as well.

4. Key piece of information describing the vMMN task is lacking (only being referred to previous studies).

Thanks for raising this point, we have added information in the amended fig1 and in the methods section; we have added information and details about the task in the methods in the "*Task and EEG recording*" paragraph in the main text and in Supplementary Information S2.

Minor issues:
1. ERP is not defined along with other abbreviations. – Thank you for spotting this missing abbreviation, we have now specified this.
2. Figure 6 is not readable. – Thank you for highlighting this issue, we have now reworked the figure to improve readability.

Reviewer #3 (Remarks to the Author):

In hallucinations of Patients with Parkinson's disease, both "bottom-up" mechanisms arising from visual dysfunction and "top-down" processes involving attentional deficits have been implicated. This paper elucidates the relationship between these two mechanisms using a causality model and addresses types of hallucinations (minor, multimodal and complex hallucinations), representing a novel contribution of significant research value.

However, as the authors acknowledge, this study constitutes an exploratory investigation of computational models using task-based EEG data, and numerous factors related to hallucinations remain uncontrolled. This limitation warrants careful consideration in the discussion. Additionally, while the analytical methods are consistent and the statistical approaches are sound, the limited sample size necessitates modest discussion.

1.
This is a fundamental clarification, but should "IFG" in Figure 1D be considered an error for "ITG"? –

Apologies for this, it is indeed a typo and thank you for spotting it, we have now corrected this.

2.
The image quality of Figure 6 is poor, so please replace it with a more readable figure, even if not high-resolution. –

Apologies for this, due to an error in uploading the readability was indeed low despite the image being vectorised. We have now uploaded the high-resolution vectorised figures separately and have worked on improving the readability of the figure.

3.
In hallucinations or PwPD, two types can be considered: hallucinations and cognitive dysfunction related to cholinergic and 5-HT circuits associated with disease progression, and drug induced hallucinations associated with dopaminergic stimulation (Pagonabarraga et al. Nat Rev Neurol 2024). This study appears to address hallucinations associated with disease progression, and the authors' logic, mainly focuses on 5-HT (and D1), is sound. However, it would be better to mention whether patients with suspected drug-induced hallucinations are included. Given that patients are taking LED 475.63±320.27mg (from SI. 1), some patients perhaps taking LED 800mg or more included. And, since participants' medication dosages are likely relevant, I have commented on this subsequently.

Thank you for specifying this concern, indeed this point should be directly addressed in the manuscript. all We have clarified this point in the revised manuscript. All participants were assessed by their treating consultant prior to inclusion, and those with suspected drug-induced hallucinations were excluded. In all cases, adjustments to dopaminergic medication had already been attempted clinically to mitigate hallucinations, and those who continued to experience visual hallucinations were considered to have disease-related (non–drug-induced) symptoms**.** This was carefully verified because several of these patients were also enrolled in a parallel pharmacological study (see Supplement; the pharmacological study was reported as proof of concept study in our ERP paper published last year).

We have now added Supplementary Table 3**,** detailing for each participant the type and dosage of antiparkinsonian medications. Although LEDD values were comparable between groups, we also re-ran the main regression models with leave one out validation including LEDD as a covariate; results were unchanged, supporting the robustness of our findings. We did not include LEDD as a covariate in the main text models to avoid overfitting given the sample size. This clarification has been added to the Methods.

We have also reference the very relevant review by Pagonabarraga and colleagues in the discussion.

4.
In Figure 3D, while connectivity from lt.V1 to lt.ITG shows an increase, the right side demonstrates a decrease. How should this laterality be interpreted? –

Thank you for this thoughtful question. We do not have a definitive answer for this, however we have a few possible interpretations. The observed laterality likely reflects a combination of factors. The first one is age: the model includes ‘age’ as a covariate. When removing age, this is the only connection in the task matrix that does no longer reach the pp>.99 threshold. In reviewing the manuscript, we had shifted to analyses not including *age* as none of the other models (e.g. multiple regressions) included it. However, we report in the main text results from both models, as we think it’s an interesting point that might suggest an ageing-related abnormality possibly associated with disease progression, however we cannot measure this. In the supplement additional details are provided. A second point concerns the association of right hemisphere anomalies with psychiatric symptoms. In our case the RH predominance is consistent with prior evidence of right-lateralised vulnerability in psychiatric and hallucinatory phenomena **(**e.g. Gainotti 2019; Corlett et al. 2019), and with our previous finding **of** greater cortical thinning in RH ventral visual regions in PD+VH **(**Vignando et al., 2022, *Nat Commun*). Moreover, the relationship between RH connectivity changes **and** complex visual hallucination severity aligns with findings from D’Antonio et al. (2022) cited in our article. The reduced LH V1→ITG connection may instead relate to minor hallucinations or reflect a more general sensory deficit.

We also note that additional contributions from attention or salience networks, not explicitly modelled here, could underlie the observed asymmetry.

5.
From a clinical perspective, the specific medication content is more important than LED for hallucination effects. For instance, even with an LED of 900mg, hallucination risk would generally be considered different between a patient taking LD/CD 600mg + COMT-I versus a patient taking high-dose dopamine agonists. It would be desirable to specify the administered medications (presentation in supplementary materials would be enough).

It would be desirable to specify the presence/absence of cholinesterase inhibitors (presentation in supplementary materials would be acceptable).

Thank you for raising this point; we have added additional information, for each individual participant, regarding the specific medications administered have been added and described in S2 as Supplementary Table 3 where we also report a chi-squared test to ensure that PD-VH and PD-noVH did not differ in terms of dopamine agonist treatment, finding that the two groups did not differ statistically (p =.2, X^2 = 2; missing information for 2 participants).

6.
In the spirit of Open Science, facilitating comparison with other studies is important. Given that UPDRS is used as a standard in many studies and PD cohorts, if UPDRS scores are available (S11), they should be disclosed. Furthermore, UPDRS part 1 item 2 serves as an extremely convenient assessment scale for visual hallucinations. If this item correlates with brain networks and proves to be a valid indicator,

clinicians would find this highly reassuring. Conversely, if this item fails to reflect brain networks and proves ineffective, this would also constitute scientifically valuable knowledge.
This comment does not insist that UPDRS must be used (Movement Disorder Society permission procedures may also be involved). Rather, it is offered as a suggestion for potentially increasing common indicators with other research.

We agree that this is a limitation of the study and that the UPDRS would enhance comparability across studies. Indeed, we looked at whether there were reliable methods to translate the scores of the SCOPA-motor and CISI-PD in UPDRS scores but there is no consensus yet on how to do this (whereas UPDRS scores can be more easily translated as SCOPA-motor scores).

At the time of data collection, the clinical battery built with consultants involved in the study oversight was standardised using alternative Parkinson's-specific scales (SCOPA-Motor, NPI, SAPS-PD). We now clarify this limitation and report UPDRS scores available within the study timeframe in the Supplementary Information. The SCOPA-Motor scale correlates highly with UPDRS Part III (Marinus et al., 2004; Rodríguez-Blázquez et al., 2013), but no validated bidirectional conversion exists. We therefore report raw SCOPA-Motor scores rather than attempting to translate them into UPDRS units.

To strengthen clinical relevance, we report SCOPA-Motor subscales in the supplement and have included additional regression analyses using SAPS-PD scores, reported in S7. SAPS-PD is a more general measure of Parkinson's psychosis if compared to the NEVHI, which is specific for MH and complex VH. We find a positive relationship for increased connectivity from right V1 to right ITG, as we found for complex VH (NEVHI score). We do not find any significant results for the other connections where we found a correlation with NEVHI complex VH. We suggest that SAPS-PD captures a global hallucination score but it does not measure the fine-grained differences that NEVHI finds; indeed, when exploring possible relationships with other hallucinations subtypes using the NEVHI, we do not find the same results as for CVH, suggesting that it is the specific severity of fully formed recurrent VH that we are observing. This is consistent with our MMN ERP study, where we found MMN amplitude at parieto-occipital level (POZ) correlated with NEVHI CVH score and for SAPS we found a relationship for the frontal electrode FZ only.

We note in the limitations that future studies incorporating UPDRS hallucinations item could directly assess the clinical utility of the identified network markers.

Minor Comment

7. Units should be added to the figures (e.g., vertical axis in Figure 1C (μV), legend on the right (ms), etc.). This has been done for all figures; for what concerns the simulation figures, we state that we are using arbitrary units rather than read amplitudes. For what concerns Figure 6, the signal is deviant-standard from source-localised data, so we have not used amplitude on the y axis.

8. While the figures appear to show 95% confidence intervals, these seem to go unmentioned. Explanation and numerical values should be provided. The details about confidence intervals were provided in full the supplement only to comply with word count; with the permission of the editor we would be happy to integrate these details in the main text. For cross-validated models (LOOCV),

performance was summarised using RMSE, MAE, and R^2, which better capture predictive accuracy across folds than confidence intervals on single model coefficients.

We wish to thank all the reviewers for the time and the attention provided to improve our manuscript. Below a point-by-point response is provided.

Reviewers' Comments:

Reviewer #1 (Remarks to the Author):

The authors responded to all my comments. I don't have additional comments

Many thanks to Reviewer #1 for their previous comments that have proven very insightful and helpful in improving the manuscript.

Reviewer #2 (Remarks to the Author):

Many thanks to Reviewer #2 for their additional comments, and in particular for the focus on the PET-maps analyses and highlighting that these need to be further clarified. Below we provide a point-by-point response to each comment.

In this revision, the authors' rewriting make the manuscript much clear now. There are still a few key concerns about how the MMN signal and PET receptor maps correlation was done and interpreted.

1. In Linear mixed model setup, only interaction term is included. This make the model hard to explain since the main and interaction effects are intertwined. Both receptor and VH effects should be listed in the model to isolate the interaction term.

We apologise for the lack of clarity. In our analyses we specified the following linear mixed-effects model:

*MMN_signal ~ receptor * VH + age + LEDD + sex + onset + MoCA + (1 | Participant)*

We used lme4 in R; in this notation, the term receptor * VH expands to both interaction and main effects, so to receptor + VH + receptor:VH, so the model includes both main effects (receptor, VH) and their interaction, as suggested by the reviewer. The random intercept for *Participant* accounts for repeated regional measurements within each participant. We have now explicitly clarified this in the Methods and have revised the text to clearly distinguish the interpretation of the main effects and the interaction terms. All three terms (receptor, VH, and receptor:VH) are reported in the *Results* tables provided in *Supplementary Information S12.* For clarity, we provide below an example of the linear mixed-effects model output for the 5-$HT2_A$ receptor, highlighting the main effects and the interaction term:

Serotonin 5-HT2A

Term	Estimate	Std. Error	df	t value	Pr(>|t|)	Significance
(Intercept)	-0.0019	0.0020	30	-0.94	0.3549	
ht2a_s	0.0012	0.0005	2810	2.126	0.0336	*
VH	0.0045	0.0018	30	2.52	0.0173	*
age_s	0.0001	0.0009	30	0.087	0.9312	
LEDD_s	-0.0006	0.0010	30	-0.537	0.5951	
sexmale	0.0046	0.0020	30	2.242	0.0325	*
onset_s	0.0003	0.0009	30	0.375	0.7099	
MoCA_s	0.0005	0.0009	30	0.561	0.5787	
5ht2a_s:VH	0.0015	0.0007	2810	2.058	0.0397	*

2. "Individual level" was used to indicate LMM analysis. But it seems all subjects are included. It is very confusing. Shouldn't it be group level analysis for the fixed effects.

We apologise for the confusion, "individual level" was used to address the fact that in the LMM we used a different approach from the one used for the first step of this exploratory section. We began by using a region-based approach to examine PET atlas to connectivity regressions and correlations ran with the spatial autocorrelation correction; this was motivated by the fact that spatial autocorrelation correction can only be applied to region-level data. However, we also wanted to further test the robustness of results using an approach that allows us to include individual variance and individual relevant covariates, such as demographics and cognition, and most importantly Parkinson's specific covariates (disease duration and LEDD). We now replaced this with *"using the full region-by-subject dataset"* as it better described the purpose of the analysis. We now explain this more clearly writing in the results section: *"To further investigate the role of covariates, we next fitted a linear mixed-effects model to the region-by-participant dataset, including the main effects of receptor density and VH status as well as their interaction (receptor * VH), with age, LEDD, sex, disease onset, and MoCA entered as covariates, and a random intercept for participant (MMN_signal ~ _receptor * VH + age + LEDD + sex + disease onset +MoCA + (1 | Participant))."*

And in the methods paragraph:

*"For the maps surviving this 'actual' correlational analysis, we explore the possible role of relevant demographics and clinical covariates fitting a linear mixed effect model to the dataset reshaped in a region-by-subject format. We used lme4[70] and lmerTest[71] to fit the LMM including the main effects of receptor density and VH status as well as their interaction (receptor * VH), and including age, LEDD, sex, disease onset, and MoCA as covariates, and a random intercept for participant [MMN_signal ~ _receptor * VH + age + LEDD + sex + disease onset +MoCA + (1 | Participant)]. The term receptor*

** VH expands to both interaction and main effects (receptor + VH + receptor*VH), so the model includes both main effects (receptor, VH) and their interaction, reported together with additional details on the methods and the models in **S12**."*

Because the LMM it is a post hoc analysis, we consider the main effects being already determined using the more robust region-based method, which is not possible with covariates. Since the receptor effects are already determined with the region-based method, our post hoc LMMs had the main purpose to test the interaction and how individual variables might affect this; we were also trying to "break" the result obtained with the region-based approach by introducing the covariates, testing if other factors were actually contributing or driving to the result. We are not trying to isolate the interaction term but understand the contribution of the other factors.

Thus, our initial and main approach, using the regional values, responds to a specific question: *Is the spatial pattern of the VH-related MMN effect aligned with dopamine, serotonin and acetylcholine receptor density?* This is a conceptually clean and standard approach that has been previously used in MRI studies (Hansen et al 2023; Lawn et al., 2024; Vignando et al., 2022). We followed this using the LMM as a post-hoc test, since brainSMASH works at the region level for group averaged data, and thus doesn't know about subjects and individual level covariates. We felt it was important to understand the potential contributors of age, LEDD and MoCA and VH group. To "stress-test" the observed relationships with a model that does know about individual participants using the same regional receptor values as predictors (fixed effect: receptor), adding VH and covariates to test whether the receptor–MMN slope different in VH vs noVH and account for repeated measures over regions within each subject via the random intercept. This model is not ideal for inference on the main correlation across regions as it is not corrected for spatial autocorrelation like the primary analysis. In summary, our primary analysis was a group-level map × map correlation, spatially corrected and our post-hoc/secondary a subject-wise LMM on region × subject data, to see if the effect survives clinical covariates and whether it differs by VH group.

3. In figure 6B, it is clear that MMN signal and D1 binding driven by two cases at far right. I would suggest that the author run a sensitivity analysis without these two cases.

We have used a formal approach (Cook's distance) for outliers' detection and removal and the results presented in the manuscript followed this procedure. Following this comment, we have further inspected the dataset, removed by hand these additional extreme values and reran the analyses. The result do not change, there is still a significant negative relationship with MMN signal and D1. Specifically, $t = -3.279$, $p = .002$, $F(1,66)=10.75$.

4. More fundamentally, it is not clear to me how the receptor map from a normal population should correlate with a diseased population and how these results can explain disease mechanisms. Please discuss it in the discussion section.

The reviewer is correct in that normative PET maps do not capture disease-related changes in *absolute* receptor availability. However, a substantial body of work has demonstrated that the large-scale spatial *gradients* (or relative regional hierarchy) of neurotransmitter receptors are highly conserved across individuals (Hansen et al., 2022) and remain broadly preserved even in neurological disorders (Hansen et al., 2023), and that these normative gradients can meaningfully account for inter-regional patterns of functional alteration across a wide range of diseases, including Parkinson's disease (Kasper et al., 2024;

Vignando et al., 2022), psychiatric, neurological and developmental disorders (Hansen et al., 2023; Lawn et al., 2024), and pharmacologically induced functional reorganisation (Luppi et al., 2023).

In the article, the value of using these normative receptor maps is in using these spatial gradients as an index of relative regional density, not as an estimate of absolute receptor levels in our cohort. The aim is to explore whether differences in activity associated with the task in PD with and without VH were related to the spatial distribution of neurotransmitter receptors that are considered to play a role in this phenomenon. This approach is conceptually similar to the widespread use of the Allen Human Brain Atlas (derived from six post-mortem brains) to relate regional gene expression patterns to structural and functional alterations in a wide range of disease, including Parkinson's disease (Rittman et al., 2016; Freeze et al., 2018; Freeze et al., 2019; Keo et al., 2020).

We also acknowledge that disease-specific PET maps would add valuable insights, but these are not available in our cohort and rarely available in the field. The availability of individual PET data, but it would be very invasive especially if multiple transmitter systems are being considered. Our exploratory analysis therefore leverages normative gradients as a biologically informed spatial prior, allowing us to test whether the pattern of MMN alterations aligns with known neurochemical distributions.

We have expanded on the limitations of this approach in the limitations section of the discussion summarising the information reported here. This part of the discussion now reads: "… In addition, our receptor-binding analyses are entirely exploratory, as we acknowledge that both source reconstruction of EEG signal and receptor density maps have a low spatial resolution. The vAChT map we used was developed on data from both healthy and Alzheimer's disease participants, thus it might partly reflect AD-related patterns. We validated our finding with a sensitivity analysis using another VAChT map (N=4 healthy controls), that by itself would have been a less realistic fit for our study. The value of using these normative receptor maps here lies in leveraging normative gradients as a biologically informed spatial prior, allowing us to test whether the pattern of MMN alterations aligns with known neurochemical distributions. We acknowledge that using individual PET data would be the ideal approach, however we did not collect PET data and collecting this type of data from multiple tracers per participant is a highly invasive procedure. We also acknowledge that disease-specific PET maps would add valuable insights, but these are not available in our cohort and rarely available in the field."

Articles referenced:

Kasper, Jan, et al. "Resting-state changes in aging and Parkinson's disease are shaped by underlying neurotransmission: A normative modeling study." *Biological Psychiatry: Cognitive Neuroscience and Neuroimaging* 9.10 (2024): 986-997.

Luppi, Andrea I., et al. "In vivo mapping of pharmacologically induced functional reorganization onto the human brain's neurotransmitter landscape." *Science advances* 9.24 (2023): eadf8332.

Hansen, J. Y., Shafiei, G., Voigt, K., Liang, E. X., Cox, S. M., Leyton, M., ... & Misic, B. (2023). Integrating multimodal and multiscale connectivity blueprints of the human cerebral cortex in health and disease. *PLoS biology*, *21*(9), e3002314.

Hansen, Justine Y., et al. "Mapping neurotransmitter systems to the structural and functional organization of the human neocortex." *Nature neuroscience* 25.11 (2022): 1569-1581.

Lawn, Timothy, et al. "Normative modelling of molecular-based functional circuits captures clinical heterogeneity transdiagnostically in psychiatric patients." *Communications Biology* 7.1 (2024): 689.

Rittman, T., Rubinov, M., Vértes, P. E., Patel, A. X., Ginestet, C. E., Ghosh, B. C., ... & Rowe, J. B. (2016). Regional expression of the MAPT gene is associated with loss of hubs in brain networks and cognitive impairment in Parkinson disease and progressive supranuclear palsy. *Neurobiology of Aging*, *48*, 153-160.

Freeze, Benjamin, et al. "Regional expression of genes mediating trans-synaptic alpha-synuclein transfer predicts regional atrophy in Parkinson disease." *NeuroImage: Clinical* 18 (2018): 456-466.

Freeze, Benjamin, et al. "Regional transcriptional architecture of Parkinson's disease pathogenesis and network spread." *Brain* 142.10 (2019): 3072-3085.

Keo, Arlin, et al. "Transcriptomic signatures of brain regional vulnerability to Parkinson's disease." *Communications biology* 3.1 (2020): 101.

Reviewer #3 (Remarks to the Author):

The authors have responded appropriately to my comments. Whilst the UPDRS was not assessed, I acknowledge that the authors have adequately examined disease severity. I am grateful for the provision of important information regarding patient demographics, including medication details.

I request minor additional revisions from the authors.

1.Antipsychotic medication
The authors have conducted an exceptionally elegant EEG analysis and obtained clear results. However, it should be noted that hallucinations in PD are characterised by their fluctuating nature. For instance, they may vary over brief periods due to medication effects, daily symptom fluctuations (wearing-off phenomenon), or even the patient's mood state during assessment (Watanabe H et al., PLoS One. 2018). Many conditions prove difficult to adjust for; however, careful patient selection may permit control of certain factors.

We thank Reviewer #3 for their comment, and we agree that our analysis does not allow us to account for fluctuations in many of the symptoms, and that this is difficult to capture. However, the NEVHI, which is the main questionnaire used in the study, asks for each hallucination subtype the date of the latest experience and how many times in the previous month and in the previous two weeks this experience happened; the severity of each hallucination subtype is computed based on how many days in that month participants experienced the hallucination, the duration of each episode, and how many times during the day this is experienced. Therefore, the NEVHI score used for complex and minor hallucination does partially reflect fluctuations, even if it the estimate of severity is an average over this time. We have now added a clarification in the methods and in the limitations.

Methods: (initial paragraph) *"Briefly, the NEVHI is a semi-structured interview that allows to compute severity scores for each hallucination subtype, focussing on the most recent experiences (i.e. asking the date when the latest experience occurred, how many times it occurred in the previous two weeks and*

how often this happens in a typical month). In our study, we computed a continuous severity score by multiplying the time (number of minutes) spent hallucinating by the number of hallucinatory experiences, for each subtype, in a month[20]*. While this approach does not allow to fully account for the individual fluctuations of the symptoms, by asking the number of a specific type experience in the past month, in the past two weeks and the relative duration of each experience and whether it happens repeatedly throughout the day, it allows to factor in individual differences more efficiently than with other questionnaires. "*

Limitations: " … *In addition, a longitudinal approach would allow to better chart the individual fluctuations, for example daily fluctuations related to medication (off and on phenomenon), or even mood, as it has been observed in Lewy Body Disease*[87]*.*"

One participant each was taking clozapine and olanzapine. The description "None of the participants were on quetiapine" (page 10) is therefore misleading; it should be revised to describe that "two participants were taking antipsychotic medication. At least, the authors should acknowledge that patients taking antipsychotic medications were not excluded from the study.

The reviewer is absolutely correct, two participants were taking antipsychotic medication: one PD-VH participant was taking clozapine and one PD-noVH participant was taking olanzapine (the latter prescribed for depressive symptoms rather than psychosis). We have revised the text accordingly and clarified that patients taking atypical antipsychotics were not excluded from the study. *"Participants did not differ on sex (χ^2 = .01, p =.91, 6 F in the PD-VH group, 7 F in the PD group, 13 M in each group), age, disease duration, motor symptoms, LEDD or MoCA score (**Supplementary Tables 1,2; Supplementary Figure 1**). Two participants were taking antipsychotic medication (one PD-VH was taking clozapine and one PD-noVH olanzapine, in this case prescribed for depressive symptoms). Two PD-VH and two PD patients were on citalopram and sertraline for depressive symptoms. Two PD-VH patients were taking rivastigmine. The number of PD-VH and PD-noVH taking dopamine agonists did not statistically differ (**S1; Supplementary Table 3**). A one-way ANOVA confirms that participants of both were paying attention to the screen and executing the task, as PD-VH and PD-noVH did not differ on misses; the groups did not differ reaction times. PD-VH were less accurate than PD-noVH in detecting the type of cross-change, but their performance was above chance level (all details **S2; Supplementary Table 3** for individual medication information). "*

Cholinesterase inhibitors such as rivastigmine, which may have an comparable or greater impact on hallucinations than SSRIs, are employed as a treatment for hallucinations and can modulate brain networks (Burn D et al., Mov Disord. 2006; Pini L et al., Brain Commun. 2024). Whilst one could endlessly enumerate such minor possibilities, it is not necessary, of course, to document all of them. If SSRIs are to be described, rivastigmine warrants similar consideration.

We thank the reviewer for this helpful suggestion. We now mention rivastigmine explicitly in the main text and we have added a note in the Supplementary Information accompanying Table 3 stating: *"Two of our PD-VH participants were taking rivastigmine. Rivastigmine has been reported to have potential benefits for psychotic and non-motor symptoms in some PD cohorts (Reading et al., 2001; Sobow,*

2007), other controlled studies show limited or no effect (van Mierlo et al., 2021; see Reilly et al., 2024). Its prescription in our cohort was determined by their consultant on a case-by-case basis (NICE, 2017).”

References mentioned:

Reading PJ, Luce AK, McKeith IG. Rivastigmine in the treatment of parkinsonian psychosis and cognitive impairment: preliminary findings from an open trial. Mov Disord. 2001;16(6):1171-1174

Sobow T. Parkinson's disease-related visual hallucinations unresponsive to atypical antipsychotics treated with cholinesterase inhibitors: a case series. Neurol Neurochir pol. 2007;41(3):276-279.

van Mierlo TJM, Foncke EMJ, Post B, et al. Rivastigmine for minor visual hallucinations in Parkinson's disease: a randomized controlled trial with 24 months follow-up. Brain Behav. 2021;11(8):e2257

National Institute for Health and Care Excellence (NICE). Parkinson's disease in adults. NICE Guideline NG71. 2017.

Reilly, Siobhan, et al. "The effects of rivastigmine on neuropsychiatric symptoms in the early stages of Parkinson's disease: A systematic review." *European journal of neurology* 31.2 (2024): e16142.

2. Laterality
The authors' response regarding laterality was also satisfactory.
I request clarification on one additional point:
The Abstract states "Connectivity patterns differed with hallucination complexity: minor VH related to left hemisphere deficits, complex VH to altered top-down and bottom-up right-hemisphere connectivity." However, the Discussion notes "Minor hallucinations showed a similar but weaker trend predominantly involving left-hemisphere regions, though this did not survive multiple comparison correction" (page 16). In light of the Discussion, the language in the Abstract should be tempered accordingly.

Yes, we absolutely agree and we have edited the abstract accordingly. Though we still mention this in the discussion as we think this relates nicely to other studies like D’antonio et al., 2022, the result cannot make the abstract after removing the additional outlier participant there are no grounds for that.

We wish to thank all the reviewers for the time and the attention provided to our manuscript and to helping us significantly improve its quality and readability. Below a point-by-point response is provided.

We thank Reviewer 2. We do not know why the data on figshare was not accessible. Nevertheless, in the interest of accessibility and to ensure a principled data sharing, we now have created a spreadsheet with clinical and demographcis information and connection strengths (Ep, exponentional) for matrix A and B for every connection in the model, both for the ventral and the dorsal model. This is available with the code used on Git https://github.com/VMiri/visual-MMN-DCM-. All EEG pre-processed data are available for anyone upon request with secure sharing.

We hope this answers your concern. Many thanks once again for the time taken to review the manuscript and the thoughtful comments and suggestions.

We thank Reviewer 3 for the time and the attention spent on our manuscript and for the insightful comments and suggestions.

Sincerely,

Miriam Vignando on behalf of all authors
